# Cross-reaction between Formosan termite (*Coptotermes formosanus*) proteins and cockroach allergens

**DOI:** 10.1371/journal.pone.0182260

**Published:** 2017-08-02

**Authors:** Christopher P. Mattison, Taruna Khurana, Matthew R. Tarver, Christopher B. Florane, Casey C. Grimm, Suman B. Pakala, Carrie B. Cottone, Claudia Riegel, Yvette Bren-Mattison, Jay E. Slater

**Affiliations:** 1 Southern Regional Research Center, Agricultural Research Service, United States Department of Agriculture, New Orleans, Louisiana, United States of America; 2 Division of Vaccines and Related Products Applications, United States Food and Drug Administration, Silver Spring, Maryland, United States of America; 3 J. Craig Venter Institute, Rockville, Maryland, United States of America; 4 New Orleans Mosquito, Termite and Rodent Control Board, New Orleans, Louisiana, United States of America; 5 BioMed Bridge, LLC., Denver, Colorado, United States of America; 6 Division of Bacterial, Parasitic and Allergenic Products, United States Food and Drug Administration, Silver Spring, Maryland, United States of America; South Texas Veterans Health Care System, UNITED STATES

## Abstract

Cockroach allergens can lead to serious allergy and asthma symptoms. Termites are evolutionarily related to cockroaches, cohabitate in human dwellings, and represent an increasing pest problem in the United States. The Formosan subterranean termite (*Coptotermes formosanus*) is one of the most common species in the southern United States. Several assays were used to determine if *C*. *formosanus* termite proteins cross-react with cockroach allergens. Expressed sequence tag and genomic sequencing results were searched for homology to cockroach allergens using BLAST 2.2.21 software. Whole termite extracts were analyzed by mass-spectrometry, immunoassay with IgG and scFv antibodies to cockroach allergens, and human IgE from serum samples of cockroach allergic patients. Expressed sequence tag and genomic sequencing results indicate greater than 60% similarity between predicted termite proteins and German and American cockroach allergens, including Bla g 2/Per a 2, Bla g 3/Per a 3, Bla g 5, Bla g 6/Per a 6, Bla g 7/Per a 7, Bla g 8, Per a 9, and Per a 10. Peptides from whole termite extract were matched to those of the tropomyosin (Bla g 7), arginine kinase (Per a 9), and myosin (Bla g 8) cockroach allergens by mass-spectrometry. Immunoblot and ELISA testing revealed cross-reaction between several proteins with IgG and IgE antibodies to cockroach allergens. Several termite proteins, including the hemocyanin and tropomyosin orthologs of Blag 3 and Bla g 7, were shown to crossreact with cockroach allergens. This work presents support for the hypothesis that termite proteins may act as allergens and the findings could be applied to future allergen characterization, epitope analysis, and clinical studies.

## Introduction

Asthma prevalence has increased over recent decades and is a common chronic disease with approximately 17 million adults and 7 million US children affected [1, 2]. Induction of asthma is caused by multiple environmental and genetic factors [3, 4]. Environmental factors that have been shown to contribute to asthma include smoke, air pollutants, and exposure to allergens. One major class of allergens associated with asthma derives from insects. Insects such as cockroaches and arachnids such as dust mites are present in homes and are recognized as major causes of asthma in urban environments [5–8]. Exposure to cockroach allergens has also been correlated with increased hospitalization rates among children [9].

Insect allergies are caused by secreted venom and feces as well as insect proteins and carcasses. There are approximately 70 species of cockroach in the US, but only a few of these inhabit human dwellings. Cockroaches are hemi-metabolous insects and some species, including the German cockroach (*Blattela germanica*), have evolved to co-exist with humans in urban environments. Several cockroach allergens and their related isoforms have been characterized [10, 11]. These allergens have been designated Bla g [1–9, and 11] for the German cockroach and Per a 1–3,6,7,9–12 for the American cockroach (*Periplaneta americana*) [12] (www.allergen.org). Cockroaches are ubiquitous in many areas of the US, and regional variation can have a significant impact on the type of indoor allergens [13]. Cockroaches can thrive in many types of environmental conditions, and humid climates such as the gulf-coast states provide a fertile environment. Cockroaches, even with the resurgence of bed bugs, remain one of the most highly targeted pests for urban pest control.

Along with cockroaches, termites live within and around housing in some areas of the country and pose a significant pest problem. There are four main species of termite living in the US: the Drywood (warmer climate states), Dampwood (Pacific coast and Southwest US), Subterranean (found in every state in the US except Alaska), and Formosan subterranean termites (Southern states). The invasive Formosan subterranean termite (*Coptotermes formosanus*) has spread to several gulf-coast states and each year it is estimated that these termites are responsible for billions of dollars in damage and control costs [14, 15]. Though native *Reticulitermes* spp. are ubiquitous along the southeastern United States, *C*. *formosanus* is known to outcompete native termites in New Orleans [16]. This is most likely due to their aggressive nature or large colony size, as there can be several million individual termites within a *C*. *formosanus* colony [17].

Cockroaches and termites are closely related insects within the order Blattaria [18]. There are no published reports documenting asthmatic or allergic reaction to termite proteins, but due to the evolutionary relationship between the two insects, it seems likely that termite proteins might cross-react with cockroach allergens. This hypothesis was tested by identifying termite sequences with homology to known cockroach allergens and testing whether Formosan subterranean termite proteins were recognized by antibodies to cockroach allergens.

## Materials and methods

### Materials

Cockroach allergic human serum pool S1Cr [19] and four other pools (P1-P4) were prepared from 61 cockroach allergic individuals using the Inner City Asthma Consortium (ICAC) patient pool collected from Baltimore (Johns Hopkins Institutional Review Board), Chicago (Children's Memorial Hospital Institutional Review Board), Denver (National Jewish Institutional Review Board), and Washington sites [7]. Subject volunteers provided informed written consent, following study review from the Institutional Review Boards of member sites; with supervision from an independent Data and Safety Monitoring Board of the Inner City Asthma Consortium at NIAID, and were collected under IND protocol 11319. The samples used in immunoassays were supported by skin prick testing [20]. Human plasma samples for the competitive ELISA to directly compare IgE binding between termite and cockroach extract were purchased from PlasmaLab International (Everett, WA, USA) and were supported by documented cockroach allergy and ImmunoCAP results (≥9 kU/l). Purified single chain variable fragment (scFv) antibodies generated against German cockroach whole body (GCr) extracts and anti-cockroach allergen specific antibodies are mentioned elsewhere [21–23]. The mouse monoclonal anti-Bla g 1 antibody (MA-10A6) was purchased from Indoor Biotechnologies (Charlottesville, VA, USA). IRDye 800CW labeled donkey anti-rabbit and donkey anti-mouse secondary antibodies were purchased from LI-COR (Lincoln, Nebraska, USA).

### Insects

Formosan subterranean termites were collected from rolls of cardboard placed in bucket traps buried near the base of trees located in New Orleans City Park (New Orleans, LA, USA). All Formosan termite samples were obtained from well-characterized Formosan termite colonies. The collection was brought back to the lab and the termites were removed from the collection material. Sequencing of 16s mitochondrial ribosomal DNA was used to verify their identities as *Coptotermes formosanus* [24]. Isolated termites were placed into plastic containers, and collected insects were frozen on dry ice and stored at -80°C. German cockroaches (*Blatella germanica*) were kindly provided by Ameya Gondhalekar (Dept. of Entomology, Purdue University, West Lafayette, IN, USA) from an established laboratory colony.

### Nucleotide sequences

Nucleotide sequences for German cockroach (*Blatella germanica)* and American cockroach (*Periplaneta americana)* allergens were obtained from GenBank. Cockroach allergen nucleotide sequences were compared to sequences from two sources of in-house *Coptotermes formosanus* sequence libraries. Partial gene sequences identified from a termite expressed sequence tag (EST) library [25, 26] and a draft whole termite genome were compared to cockroach allergen sequences using Blast-2.2.21 software (Blastn and tBlastx). *C*. *formosanus* gene sequences that matched those of cockroach allergens were identified S1 Fig and the top matching sequences were translated and aligned to the homologous cockroach allergen sequences. Specialized BLAST to align two sequences, available at NCBI’s web server (http://blast.ncbi.nlm.nih.gov/Blast.cgi), was used to align the homologous sequences using the translated BLAST program, tblastn. Total query coverage and total score of the alignments were recorded. The maximum score among all the High Scoring Pairs (HSPs) was noted for each alignment, and the corresponding e-value, identity percentage, and positives percentage were recorded.

### Protein extracts

A frozen mixture of soldier and worker termites (*C*. *formosanus*), or cockroaches (*B*. *germanica*) was pulverized with a chilled mortar and pestle, followed by defatting with petroleum ether using a BUCHI B-811 Standard extraction unit (BUCHI Labortechnik AG, Flawil, Switzerland). Residual petroleum ether was removed from the defatted samples by air-drying in a fume hood, and the material was re-ground to a fine powder with a chilled mortar and pestle. Powdered samples were extracted with PBS containing 1mM PMSF for 1 hr with mixing at 4°C. Samples were centrifuged at 4°C for 30 min at 16,000 RCF to remove insoluble material, protein concentration was determined with a NanoDrop (ThermoFisher, Pittsburgh, PA, USA) instrument, and sample aliquots were frozen and stored at −80°C.

### SDS-PAGE and immunoblotting

Termite and cockroach protein samples (25 μg/lane) were resolved on Novex 4–20% Tris-Glycine or 4–12% Bis-Tris gels (Life Technologies). NuPAGE LDS sample buffer (Life Technologies) was added in a 4:1 v/v ratio. Proteins were visualized using Safe Stain (Life Technologies, Carlsbad, CA, USA) and gel images were visualized using an Odyssey CLX infrared imaging system (LI-COR, Lincoln, NE, USA). Immunoblots using rabbit and mouse full-length antibodies were performed as described in Mattison et al., 2014 [27], except secondary antibodies were IRdye800 labeled donkey anti-rabbit and donkey anti-mouse antibodies (LI-COR). Immunoblots using IRdyes were visualized using an Odyssey CLX infrared imaging system (LI-COR, Lincoln, NE, USA). For immunoblots using scFv antibody fragments, proteins separated by SDS-PAGE were transferred onto PVDF membranes (0.2 μM, Life Technologies). After blocking with Starting Block (Life Technologies), the membrane strips were incubated with scFv (1 μg/mL diluted in Starting Block). The binding efficiency of scFvs was detected using HRP conjugated anti-HA antibody (1:2000 in PBS-T + 3% BSA). The HRP signal was quantified using a Densitometer G Box from Syngene (Frederick, MD).

### Tryptic digestion and mass-spectrometry

Termite protein samples were processed for LC-MS/MS as described in Mattison et al., 2014 [28]. Briefly, extracted protein samples from whole termites were reduced with DTT, alkylated with iodoacetamide, and digested with sequencing-grade modified trypsin (Promega, Madison, WI, USA) overnight at 37°C. Trypsin digestions of termite samples were analyzed using the Agilent 1260 LC system, Agilent Chip-cube interface and Agilent 6520 Q-TOF tandem mass spectrometer (Agilent Technologies, Santa Clara, CA). Chromatographic separation was accomplished using a chip consisting of a 40 nL enrichment column and a 43 mm analytical C18 column, 5 μm beads with 300 Å pores. One μl aliquot of the sample was transferred to the enrichment column via the Agilent 1200 capillary pump operating at a flow rate of 4 μl/min. The Agilent 1200 nano pump was operated at a flow rate of 400 nL/min. The column solutions were Solvent A (100% H_2_O, 0.1% formic acid) and Solvent B (90% acetonitrile (ACN), 10% H_2_O and 0.1% formic acid). The initial gradient of Solvent B was set a 3% and was changed to 10% at 2 min, 50% at 25 min, 100% at 30 min, and back to 3% at 35 min. A post run time of 10 min was employed for column equilibration.

The MS source was operated at 300°C with 5 L/min N_2_ flow and a fragmentor voltage of 175V. N_2_ was used as the collision gas with collision energy varied as a function of mass and charge using a slope of 3.7 V/100 Da and an offset of 2.5 V. Both quad and TOF were operated in positive ion mode. Reference compounds Hexamethoxyphosphazine (MW 322.048121 Da) and Hexakis (MW 1221.990637 Da) were continually leaked into the source for mass calibration. An initial MS scan was performed from m/z 300 to 1600 and up to 3 multiply charged ions were selected for MS/MS analysis. Following the initial run, a second injection was made excluding ions previously targeted for MS/MS analysis.

Data files were transferred to an Agilent workstation equipped with Spectrum Mill software (Agilent Technologies, Santa Clara, CA). The raw MS/MS data files were extracted, sequenced, and searched with SpectrumMill software against a cockroach allergen specific in-house database for identical peptide matches. Following the identity search, a second degenerate search was conducted allowing for the substitution of a single amino acid per peptide. Summed MS/MS search scores with values greater than 13 were considered to be excellent matches.

### Human serum IgE immunoblotting

Termite extract samples (25 μg/lane) were subjected to SDS-PAGE and transferred to PVDF membrane (Life Technologies). Blotted membranes were blocked at room temperature in Starting Block (Life Technologies). Serum pools were diluted 1:10 in Starting Block, added to blot strips, and incubated overnight at 4°C with gentle mixing. The strips were then incubated with biotinylated anti-human IgE followed by HRP conjugated streptavidin (1:2500 in PBS). The washes were repeated and signal was visualized and quantified using chemiluminescent substrate.

### ELISA

Recognition of termite proteins by rabbit antibodies, scFvs, or individual human serum samples from cockroach allergic human serum pool was evaluated by ELISA. Microtiter plates (NUNC-ImmunoTM MaxiSorpTM 96-MicroWell, ThermoScientific) were coated with 1 μg/well of termite or cockroach protein sample in sodium bicarbonate buffer (0.015 M Na_2_CO_3_, 0.035 M NaHCO_3_, pH 9.6) and allowed to incubate overnight at 4°C. Plates were blocked with 1% BSA dissolved in PBST for 1 hr at 37°C. Diluted rabbit anti-cockroach antibodies (1:1000 in PBST), scFvs (1:1000), or human serum samples from cockroach allergic patients (1:10 in PBST) were added to the wells and incubated overnight at 4°C. IRDye labeled donkey anti-rabbit (LI-COR) or IRDye labeled mouse anti-human IgE was added to the wells and plates were incubated at 37°C for 1 hr. Antibody binding was visualized and quantified by scanning with an Odyssey CLx (LI-COR) infrared imaging system.

For the cockroach allergic human serum pool study, the wells were coated with serially diluted extract starting with 200 μg/mL. Plates were blocked with 1% BSA dissolved in PBST for 1 hr at 37°C. Diluted human serum samples from cockroach allergic patients (1:10 in PBST) were added to the wells and incubated overnight at 4°C. Plates were washed throughout the assay between incubation steps. Antibody binding was visualized and quantified with TMB substrate. All assays were performed in triplicate with mean values reported and error bars represented with +/- standard deviation.

For the competitive ELISA to directly compare IgE binding between termite and cockroach extract, a pool of 6 cockroach allergic human plasma were evaluated for binding to microtiter plate wells coated with 1 μg of German cockroach extract overnight at 4°C. Wells were blocked with 1% BSA dissolved in PBST for 1 hr at room temperature. Plasma samples (25 μL) were incubated with 25 μL of serial half-log dilutions of either termite or cockroach extract at an initial concentration of 12.5 μg/μL. Plates were washed 4 times during the assay between incubation steps. IgE binding was visualized by the sequential addition of biotinylated mouse anti-human IgE (1:1000) and IRDye680-conjugated streptavidin (1:5,000). All assays were performed in triplicate with mean values reported and error bars represented with +/- standard deviation.

### Bead based multiplex assay

The bead based multiplex suspension assay was performed as described by Khurana et al., [22]. Briefly 18 different scFv antibody-coupled beads were mixed and dispensed in pre-wet wells of 96-well filter bottom plates (Multiscreen BV; Millipore, Billerica, MA) containing serially diluted termite extract. Antibody-coupled beads were incubated with termite extract for 1 hr in the dark at room temperature with gentle mixing. The binding of scFv and proteins was detected with a mixture of rabbit antisera containing anti-E6Cg, anti-Bla g 1, anti-Bla g 2, and anti-Bla g 4 (each diluted 1:500) [23]. Biotin-coupled anti-rabbit IgG antibody (KPL, Gaithersburg, MD) was diluted (1:1000) and added to each well for detection. The binding was detected using diluted streptavidin-R-Phycoerythrin (100 μg/mL) (Thermo Scientific). Finally, the beads were resuspended in sheath fluid (Bio-Rad Laboratories) and median fluorescence intensities (MFI) were measured in a BioPlex 200 reader (Bio-Rad Laboratories).

## Results

### Sequence conservation between termite proteins and cockroach allergens

Termite sequences were collected from a previously generated expressed sequence library [25, 26] and an unpublished draft termite genome sequence. Homology to cockroach allergens was identified using sequence from 16 cockroach allergens listed in the International Union of Immunological Societies (IUIS, www.allergen.org) website as queries to search the in-house databases, specific for *C formosanus*, using the BLAST program. Termite nucleotide sequences matching cockroach allergen sequences were identified and their translated protein sequences were compared for identity and similarity using pair-wise protein BLAST 2 Sequences (bl2seq) alignment. Several predicted termite proteins had greater than 60% identity along the length of the alignment to cockroach allergens Table 1 and S1 Fig. For example, the top *C*. *formosanus* tropomyosin homolog (4474.m000137) was greater than 80% similar to both the Bla g 7 and Per a 7 tropomyosin allergens from German and American cockroach along the full-length of the protein (100% coverage). No termite sequence matches to the Bla g 4 allergen gene were identified, and matches to the Bla g 1/Per a 1, and Bla g 2/Per a 2 allergens were found on contiguous sequences that were not completely annotated. The match to Per a 1 was found on assembly contig_55012 with sequence coverage of 98% and similarity of over 60%. The matches to Bla g 2/Per a 2 were found on contig_22029 and contig_22030 with over 60% similarity Table 1. Due to the fragmentary nature of the current draft genome annotation, it is possible additional sequence homologues of some cockroach allergens await discovery.

**Table 1:** Putative termite homologs of cockroach allergen genes.

Query Sequence Description	Termite Sequence ID	Termite Sequence Description	Total Query Coverage	Total Score	Max Score (among all HSPs)	E-value (of HSP with Max Score)	Identities (of HSP with Max Score)	Positives (of HSP with Max Score)
***Blatella germanica* (German cockroach)**								
**Bla g 1**	contig_55012	Assembly Contig	97%	423	129	9.00E-38	41%	56%
**Bla g 2**	contig_22030	Assembly Contig	11%	42.7	42.7	2.00E-07	46%	63%
**Bla g 2**	contig_22029	Assembly Contig	49%	197	58.9	3.00E-12	53%	62%
**Bla g 3**	1481.m000236	Allergen Cr-PI, putative	99%	764	764	0	56%	74%
**Bla g 3**	1481.m000238	Hexamerin subunit precursor	55%	392	392	9.00E-130	53%	72%
**Bla g 4**	NONE	No significant hits found	NA	NA	NA	NA	NA	NA
**Bla g 5**	4541.m000139	Glutathione S-transferase	99%	226	226	1.00E-78	58%	67%
**Bla g 6**	57.m000588	Allergen Bla g 6. 0301, putative	94%	209	209	2.00E-73	72%	88%
**Bla g 6**	14777.m000067	Troponin C type II a family protein	96%	172	172	1.00E-59	59%	76%
**Bla g 6**	4607.m000136	EF hand domain-containing protein	90%	213	167	7.00E-58	92%	95%
**Bla g 6**	809.m000404	Calmodulin putative	97%	180	118	3.00E-38	43%	69%
**Bla g 7**	4474.m000137	Tropomyosin 2 family protein	99%	317	317	4.00E-112	66%	81%
**Bla g 7**	1264.m000365	Tropomyosin Putative	86%	234	234	1.00E-79	58%	69%
**Bla g 8**	4958.m000213	Myosin light chain	85%	311	311	2.00E-112	90%	95%
**Bla g 8**	5766.m000242	Myosin II Regulatory Light Chain	65%	124	124	6.00E-40	49%	66%
**Bla g 9**	NONE	No significant hits found	NA	NA	NA	NA	NA	NA
**Bla g 11**	3431.m000139	1,4-alpha-D-glucan glucanohydrolase precursor	97%	739	739	0	69%	82%
**Bla g 11**	13638.m000105	Alpha-amylase	54%	460	460	1.00E-164	75%	86%
**Bla g 11**	27662.m000053	Alpha-amylase	39%	292	292	2.00E-100	67%	78%
***Periplanata americana* (American cockroach)**								
**Per a 1**	contig_55012	Assembly Contig	98%	277	161	2.00E-51	47%	62%
**Per a 2**	contig_22030	Assembly Contig	37%	159	89.7	1.00E-22	52%	67%
**Per a 2**	contig_22029	Assembly Contig	48%	242	63.9	7.00E-14	55%	73%
**Per a 3**	1481.m000236	Allergen Cr-PI, putative	100%	864	864	0	61%	75%
**Per a 3**	1481.m000238	Hexamerin subunit precursor	99%	706	706	0	51%	65%
**Per a 3**	350.m000777	Prophenoloxidase	86%	259	259	9.00E-80	30%	48%
**Per a 3**	11697.m000092	Prophenol oxidase subunit 2	47%	135	135	6.00E-39	31%	50%
**Per a 6**	57.m000588	Allergen Bla g 6. 0301, putative	94%	213	213	3.00E-75	74%	89%
**Per a 6**	14777.m000067	Troponin C type II a family protein	96%	178	178	5.00E-62	62%	76%
**Per a 6**	4607.m000136	EF hand domain-containing protein	90%	211	161	2.00E-55	91%	95%
**Per a 6**	809.m000404	Calmodulin putative	97%	183	120	6.00E-39	43%	70%
**Per a 7**	4474.m000137	Tropomyosin 2 family protein	100%	320	320	2.00E-113	67%	81%
**Per a 7**	1264.m000365	Tropomyosin Putative	86%	236	236	2.00E-80	59%	69%
**Per a 9**	1950.m000177	Arginine Kinase	84%	613	613	0	96%	99%
**Per a 9**	1322.m000276	ATP:guanido phosphotransferase	99%	118	118	1.00E-32	25%	44%
**Per a 9**	4552.m000111	Arginine Kinase	16%	103	103	4.00E-32	91%	98%
**Per a 10**	2550.m000162	MPA3 allergen, putative	92%	333	333	9.00E-120	68%	78%
**Per a 10**	26042.m000070	MPA3 allergen, putative	100%	296	296	6.00E-105	59%	69%
**Per a 10**	2550.m000161	MPA3 allergen, putative	91%	263	263	5.00E-92	56%	69%
**Per a 10**	28569.m000019	MPA3 allergen, putative	70%	223	223	1.00E-76	66%	77%
**Per a 10**	40061.m000014	MPA3 allergen, putative	70%	211	211	3.00E-72	66%	75%
**Per a 11**	NONE	No significant hits found	NA	NA	NA	NA	NA	NA
**Per a 12**	NONE	No significant hits found	NA	NA	NA	NA	NA	NA

Protein identity and positive scores between termite and cockroach homologs determined using pair-wise protein BLAST 2 Sequences (bl2seq) alignment.

### Protein sequence conservation between termite proteins and cockroach allergens

To find evidence of the cockroach allergen homologs within termite extracts, whole termite protein (Cf) extract samples were analyzed by mass-spectrometry. Several peptides exactly matching those of *B*. *germanica* and/or *P*. *americana* allergens were identified, including tropomyosin, arginine kinase, and myosin. There were 9 peptides from the putative *C*. *formosanus* arginine kinase homolog that were matched to the Per a 9 sequence Table 2. The 9 peptides observed in the Cf extract, provided 32% coverage of the Per a 9 sequence. A couple of these peptides, 96-TDKHPPKDWGDVDTLGNLDPAGEYIISTR-124 and 194-FLQAANACR-202, lie within previously mapped T-cell epitopes for the shrimp arginine kinase Pen m 1 [29]. There were six peptide matches within the Cf extracts resulting in 17% coverage of the Bla g 7 cockroach tropomyosin Table 2. Five of these peptides also matched those of the Per a 7 tropomyosin sequence from the American cockroach. Mapped IgE epitopes for cockroach tropomyosin are not known, but some of the tropomyosin peptides that were observed, including 113-LAEASQAADESER-125, 153-FMAEEADKK-161, and 190-IVELEEELR-199 in the Cf extract, correspond to previously mapped IgE epitopes from shrimp tropomyosin [30, 31]. The mass-spectrometric analysis also uncovered 3 peptide matches to the cockroach myosin light chain protein (Bla g 8) resulting in 16% coverage Table 2. One of these peptides, 87-ELDEMVNEAPGPINFTQLLTLFAGR-111, is similar in sequence to an IgE epitope from shrimp myosin light chain [32]. A degenerate search for termite peptides whose predicted sequence missed that of cockroach allergen peptides by a single amino acid mismatch was also performed. Using this less-stringent search four additional peptides were observed. Three of the peptides matched the Per a 3 allergen, and there was a single peptide match to Per a 2 Table 3.

**Table 2:** Termite peptide matches to cockroach allergen protein sequences.

Protein	Species	Acession Number	Allergen	Peptide Start Amino Acid	Peptide Sequence	m/z measure (Da)	z	MH+ matched (Da)	MH+ error (ppm)
**tropomyosin**	B. germanica	Q9NG56	Bla g 7	113	(K)LAEASQAADESER(A)	688.819	2	1376.63	1.1
**tropomyosin**	B. germanica	Q9NG56	Bla g 7	153	(R)FMAEEADKK(Y)	356.845	3	1068.5	16.6
**tropomyosin**	B. germanica	Q9NG56	Bla g 7	162	(K)YDEVAR(K)	376.684	2	752.357	4.2
**tropomyosin**	B. germanica	Q9NG56	Bla g 7	190	(K)IVELEEELR(V)	565.307	2	1129.61	-1.1
**tropomyosin**	B. germanica	Q9NG56	Bla g 7	206	(K)SLEVSEEK(A)	460.733	2	920.457	2.6
**tropomyosin**	B. germanica	Q9NG56	Bla g 7	239	(R)AEFAER(S)	361.68	2	722.347	6.8
**myosin**	B. germanica	ABD47458	Bla g 8	74	(R)ATFDQLGR(L)	376.182	2	751.362	1.2
**myosin**	B. germanica	ABD47458	Bla g 8	87	(K)ELDEMVNEAPGPINFTQLLTLFAGR(M)	694.601	4	2775.402	0.4
**myosin**	B. germanica	ABD47458	Bla g 8	169	(K)GFINTAK(L)	375.708	2	750.414	2.6
**arginine kinase**	P. americana	167782135	Per a 9	33	(K)EVFDNLK(T)	432.728	2	864.446	3.2
**arginine kinase**	P. americana	167782135	Per a 9	96	(K)TDKHPPKDWGDVDTLGNLDPAGEYIISTR(V)	803.383	4	3210.57	-18.6
**arginine kinase**	P. americana	167782135	Per a 9	181	(K)LIDDHFLFK(E)	383.209	3	1147.62	-1.1
**arginine kinase**	P. americana	167782135	Per a 9	194	(R)FLQAANAcR(F)	525.761	2	993.494	-0.2
**arginine kinase**	P. americana	167782135	Per a 9	230	(R)IISMQMGGDLGQVYR(R)	556.614	3	1667.82	2
**arginine kinase**	P. americana	167782135	Per a 9	245	(R)RLVTAVNDIEK(R)	629.361	2	1257.72	-0.6
**arginine kinase**	P. americana	167782135	Per a 9	256	(K)RIPFSHDDR(L)	381.531	3	1142.57	6.5
**arginine kinase**	P. americana	167782135	Per a 9	304	(K)YNLQVR(G)	396.721	2	792.436	-2.7
**arginine kinase**	P. americana	167782135	Per a 9	310	(R)GTRGEHTEAEGGVYDISNK(R)	505.741	4	2019.94	2.7

Termite peptides from whole termite extract matching cockroach allergen sequences were identified by LC-MS/MS.

**Table 3:** Termite peptide matches allowing for a single amino acid substitution to cockroach allergen protein sequences.

Protein	Species	Acession Number	Allergen	Peptide Start Amino Acid	Variable Site	Peptide Sequence	m/z measure (Da)	z	MH+ matched (Da)	MH+ Mass Shift (Da)	MH+ error (ppm)
**aspartatic protease-like**	P. americana	E7BQV5	Per a 2	255	V265D	(K)INDRLGCTNKDIGSR(T)	573.6289	3	1702.902	15.9704	6.9
**hemocyanin**	P. americana	Q25640	Per a 3	252	R261G	(K)LEDVLKANIG(A)	536.3041	2	1170.684	-99.0832	-3.3
**hemocyanin**	P. americana	Q25640	Per a 3	454	V456E	(K)DVEIFHKK(Y)	508.2897	2	985.583	29.9892	14.8
**hemocyanin**	P. americana	Q25641	Per a 3	669	F673Y	(K)DVIIYHKK(Y)	508.294	2	999.599	15.9821	-12.6

Termite peptides allowing for a single amino acid mismatch from whole termite extract matching cockroach allergen sequences were identified by LC-MS/MS.

### Cross-reaction between termite proteins and cockroach allergens

**Fig 1:**
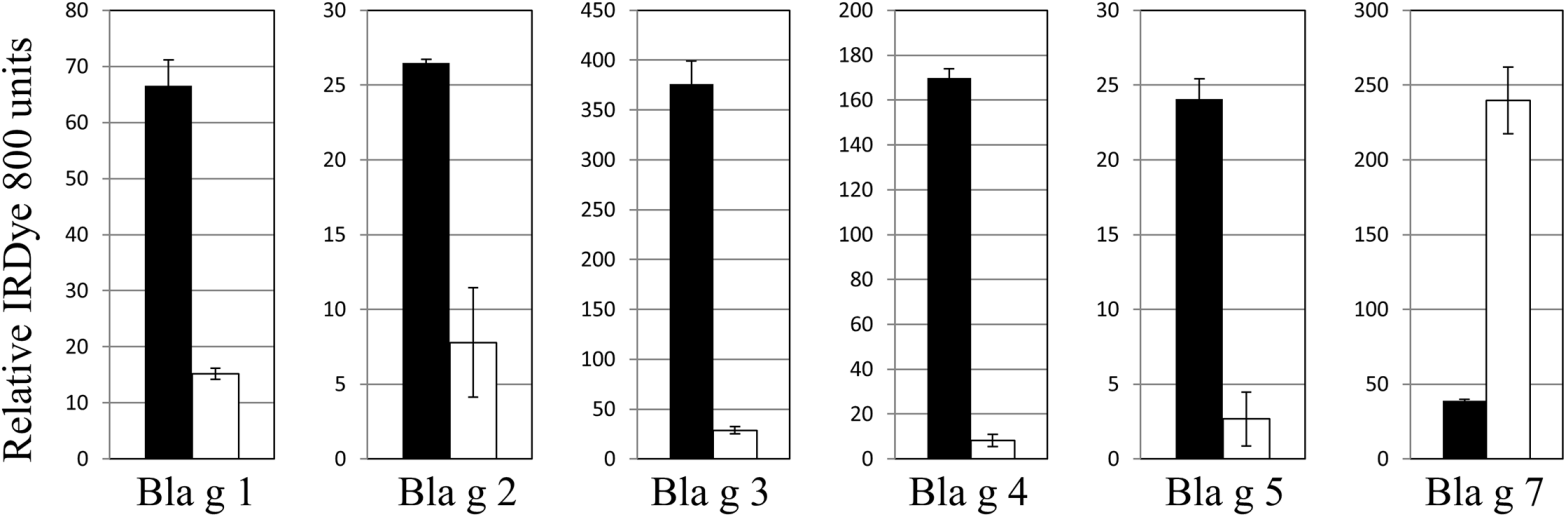
Termite proteins cross-react with anti-cockroach allergen antibodies. Cf termite extract (1 μg) or German cockroach extract (GCr, 1 μg) was added to the wells of a microtiter plate for ELISA and probed with rabbit anti-cockroach allergen antibodies (1:500) followed by IRdye 800 labeled anti-rabbit antibody (1:10000). Black bars represent GCr extract and white bars represent Cf termite extract signals. Samples were tested 4 times and mean values are shown with standard deviation included as error bars. Relative IRdye800 signal is shown on the y-axis and anti-cockroach allergen antibody designation is shown on the x-axis.

To confirm and extend the sequence homology and mass-spectrometry results, Cf extracts were probed with antibodies to several cockroach allergens. As can be seen in Fig 1, ELISA with rabbit antibodies to several cockroach allergens including Bla g 3, 4, and 5 [22, 23, 33] resulted in a relatively weak signal from the termite extract for these targets compared to the signal from the cockroach extract. The Bla g 3 signal from the termite extract was 8% that of the cockroach extract signal, and the Bla g 5 antibody in the termite extract was 11%. The Bla g 4 signal was only 5% that of the cockroach extract. The Bla g 1 and Bla g 2 signals from the termite extract were relatively higher at 23% and 29% of the cockroach extract, respectively. In contrast, the Bla g 7 antibody produced a noticeably more intense signal in the termite extract and was greater than 6-fold that of the cockroach extract even though equivalent amounts of protein were used in the ELISA. There is evidence that muscle proteins such as myosin are highly expressed in soldier termites [34], and it is likely that there is an abundant amount of tropomyosin in the termite extracts since a mix of soldier and worker termites was used to make them. The large mandibles used for feeding in workers and defense in soldiers, would presumably contain large amounts of tropomyosin and other muscle proteins. There was no observable signal above background in the termite extract samples in the absence of primary antibodies.

**Fig 2:**
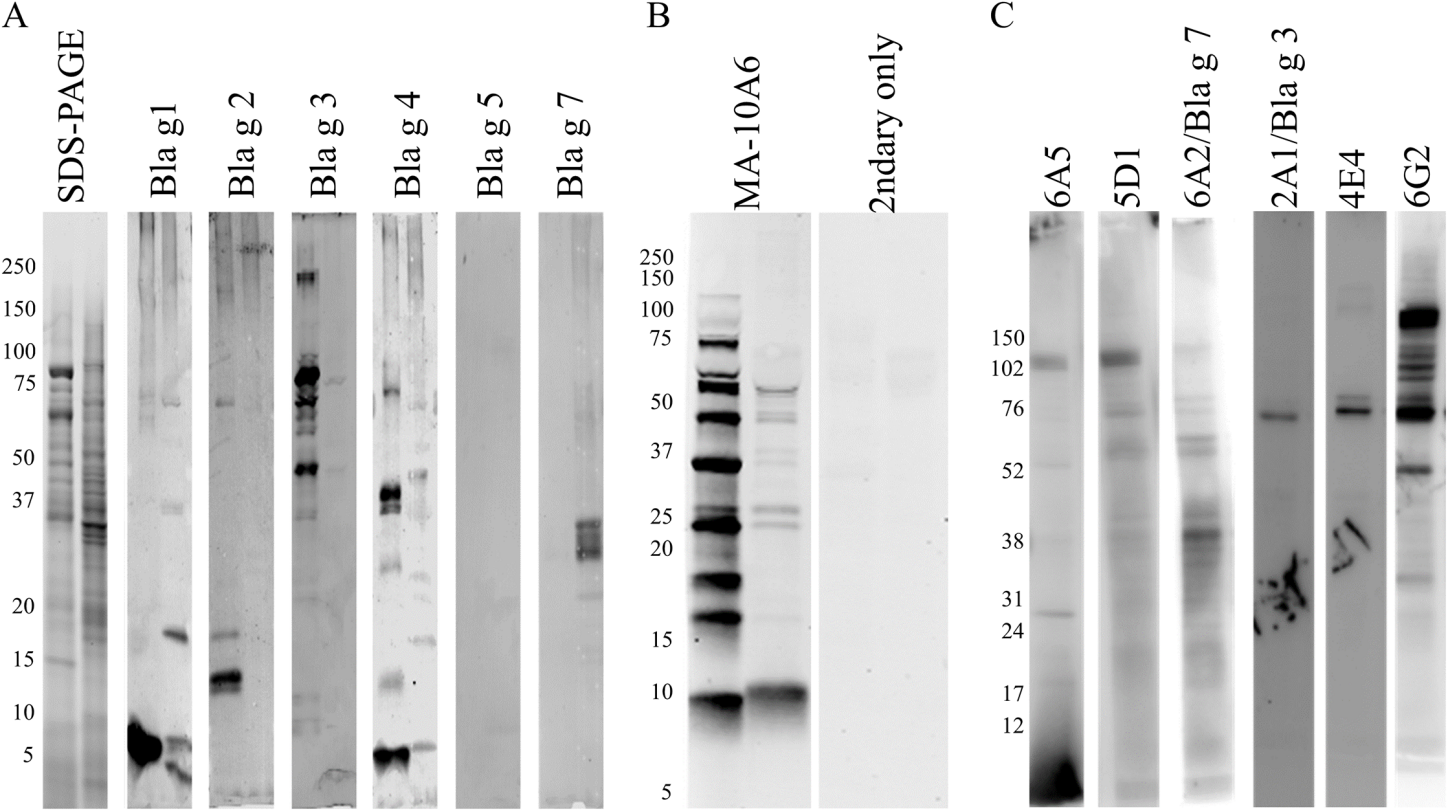
Immunoblots of termite extract with anti-cockroach allergen antibodies. (A) GCr (left lane of each panel) and Cf termite extract (right lane of each panel) (1 μg) were resolved by SDS-PAGE and stained to visualize protein or transferred to PVDF and probed with rabbit anti-cockroach allergen antibodies (1:500) followed by IRdye 800 labeled anti-rabbit secondary antibody (1:10000). Molecular weight markers are shown to the left of the SDS-PAGE gel. (B) The GCr (left lane of each panel) and Cf extracts (right lane of each panel) were probed with a monoclonal anti-Bla g 1 antibody and an IRdye800 labeled goat anti-mouse secondary antibody. Molecular weight markers are shown to the left of the PVDF membrane. (C) Western blots of Cf termite whole body extract. Purified scFvs were used as primary antibodies (1.0 μg/mL) and horseradish peroxidase-conjugated anti-hemagglutinin (1:1000) was used for detection using a chemiluminescent substrate.

To better characterize the proteins recognized by the rabbit antibodies, western blots with the termite extracts were performed. The Bla g 1 antibody recognized 2 dominant bands around 10 and 20 kDa and 2 fainter bands at 37 and 75 kDa in the termite extract (Fig 2A and S2 Fig), and a dominant band at 10 kDa in the cockroach extract. When this analysis was repeated with a mouse monoclonal anti-Bla g 1 antibody (MA-10A6), a similar banding pattern was observed. The MA-10A6 antibody recognized a dominant band around 10 kDa, and 4 fainter bands near 25 (doublet), 37, and 50 kDa (Fig 2B and S3 Fig) in the termite extract. These bands were fainter, but they mirrored the migration of bands of similar size in the cockroach extract (Fig 2B). In contrast to the ELISA signal, there was no discernible signal from the Bla g 2 or Bla g 5 antibodies in the termite extract immunoblot (Fig 2A), however this could be due to loss of conformational epitope(s) in extracts probed following the denaturing conditions of the blotting procedure for the Bla g 5 antibody because no reactivity was observed in the cockroach extract lane. The Bla g 2 antibody recognized a dominant band at 15 kDa and a fainter band at 20 kDa in the cockroach extract lane. There was a faint cross-reactive signal in the termite extract migrating near the 75 kDa marker (Fig 2A), consistent with the expected size of Bla g 3 [35], and there was an obvious band near 75 kDa in the cockroach extract lane. The Bla g 4 antibody recognized several bands of varying intensity in the termite extract, and one of these bands migrated near the 20 kDa marker, consistent with the expected size of Bla g 4 [36], but in the cockroach extract lane there were two dominant bands migrating around 45 and 8 kDa. The Bla g 7 antibody produced a readily observable signal in the termite extract composed of several bands migrating just under the 37 kDa marker (Fig 2A) consistent with the expected size of termite tropomyosin, but no signal was detected in the cockroach extract lane.

**Fig 3:**
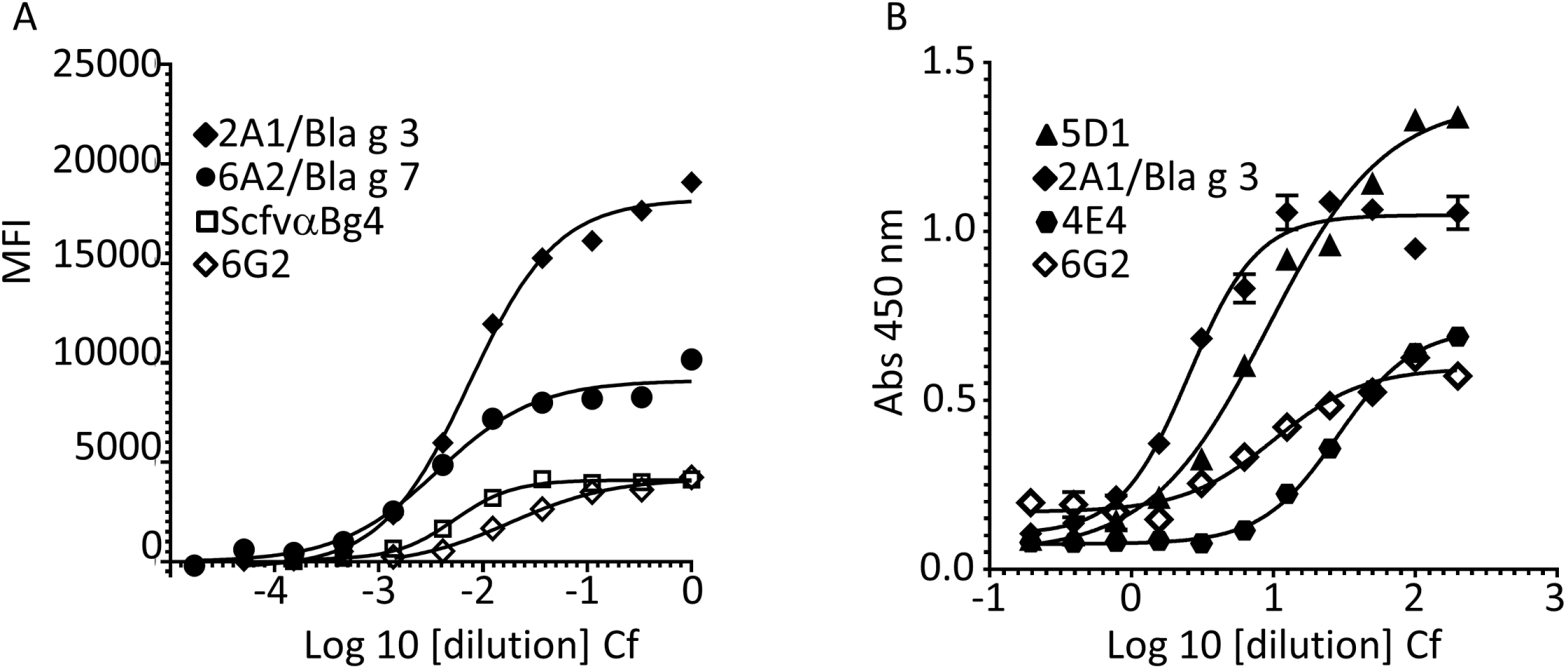
Termite proteins cross-react with anti-cockroach allergen scFvs in ELISA assays. (A) This assay was performed in sandwich format using two separate collections of detection agents. Serially diluted Cf termite extract was first added to a mixture of anti-cockroach scFvs either generated non-specifically using whole body GCr extract or specifically targeted against recombinant Bla g 1, 2, or 4, and one scFv from a naïve human library screened against whole GCr extract that were coupled to unique bead sets as capture agents in 96-well filter bottom plates. A mixture of rabbit anti-E6Cg cockroach extract polyclonal antibodies, anti Bla g 1, anti Bla g 2, and anti Bla g 4 IgGs (1:500) were used as detection antibodies, followed by biotinylated anti-rabbit (1:1000) and streptavidin-RPE (1:500). MFI detected for selected scFvs is on the y-axis. (B) Ninety six well plates were coated with serially diluted Cf termite extract overnight at 4°C. ScFvs (1.0 ug/mL) were added to each well after blocking. The binding was determined after addition of HRP-conjugated anti-HA antibodies. Samples were tested 2 times and mean absorbance values are reported. Serially diluted extract (log scale) is on the x-axis.

To expand upon the antibody results thus far, several scFv antibodies developed for cockroach allergens [21, 23, 35, 37] were screened using immunoblot and multiplex assays for cross-reactivity to termite extracts. During immunoblot screening of the 18 unique scFvs, distinct signals at a molecular weight of 70 kDa were observed with scFv 2A1 directed towards Bla g 3/hemocyanin and scFv 4E4 (target unknown) (Fig 2C and S4 Fig). scFvs 6A5 (target unknown), 5D1 (target unknown), 6A2 (Bla g 7/tropomyosin), and 6G2 (target unknown) recognized multiple proteins ranging from 17 to 102 kDa (Fig 2C). The rest of the scFvs were not able to recognize termite proteins (not shown). All 18 scFv antibodies were tested in a multiplexed bead-based suspension assay and it was determined that only 4 showed a complete sigmoidal response to the termite extract. The 2A1/Bla g 3, 6A2/Bla g 7, scFv 6G2 (target unknown), and the Bla g 4 scFvα-Bg 4 antibody fragments bound to termite proteins in this assay (Fig 3A). The MFI values of Bla g 3 scFv (2A1) and Bla g 7 scFV (6A2) were relatively higher than those of scFv 6G2 and α-Bg 4 scFv (Fig 3A). The scFv antibodies were also tested in direct ELISA with wells coated with serially diluted termite extract, and, again, only 4 antibodies showed sigmoidal responses. Consistent with the multiplex assay results the 2A1/Bla g 3 scFv responded in both assays (Fig 3B). Though scFv 5D1 did not provide a discernible signal in the multiplex assay, a more robust response was observed in the direct ELISA against the termite extract (Fig 3B).

### Cross-reaction between termite proteins and sera from cockroach allergic patients

**Fig 4:**
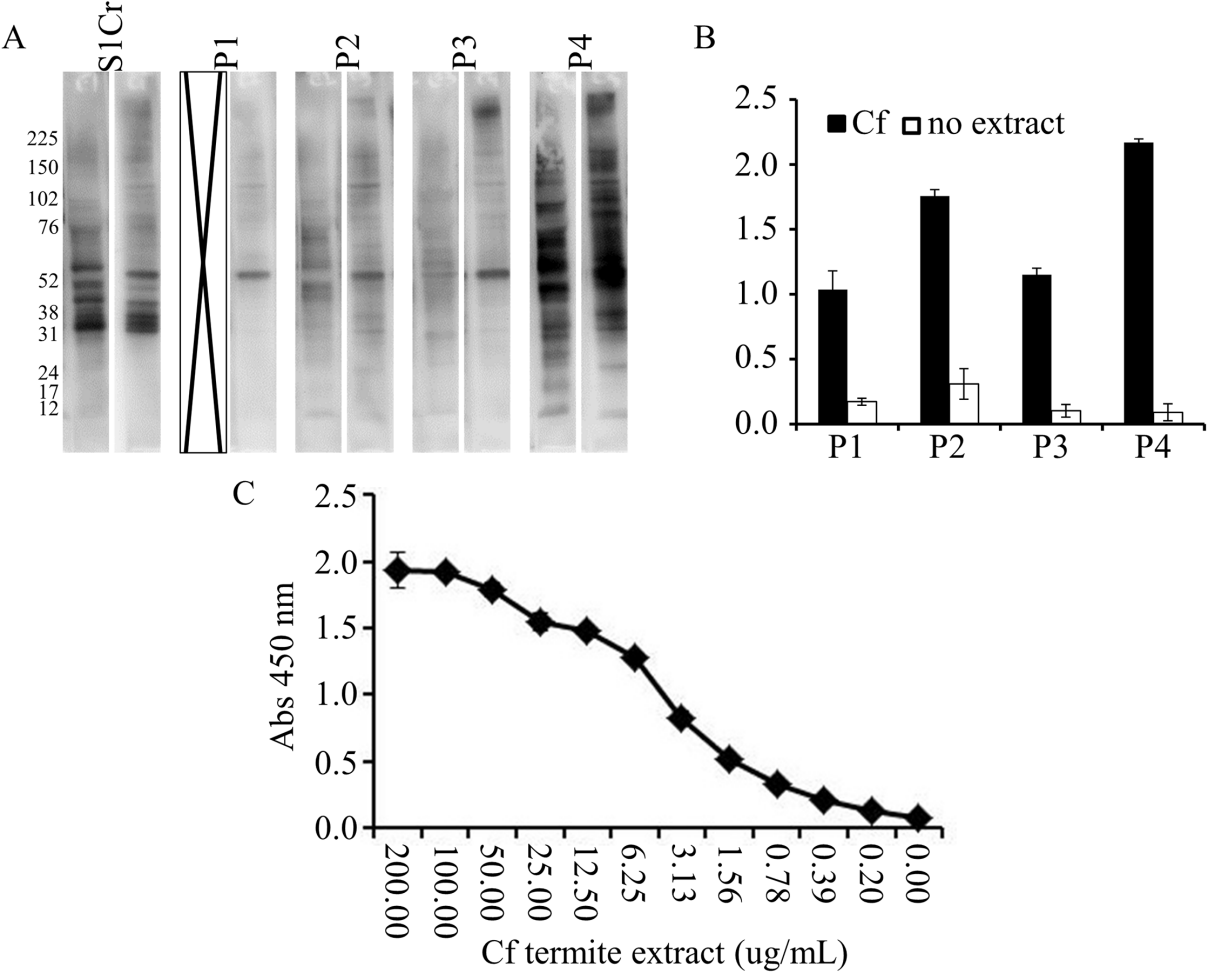
Termite proteins cross-react with IgE from human cockroach allergic sera. (A) Western blot of GCr (left lane of each panel) and Cf termite (right lane of each panel) extracts. The proteins were separated on SDS-PAGE gel followed by transfer onto PVDF membrane. After blocking for 2 hr the blot was incubated with four GCr allergic serum pools S1Cr and P1-4 (1:10). Specific IgE binding was determined following sequential addition of biotinylated goat anti-human IgE (1:1000), and HRP-conjugated streptavidin (1:10,000). (B). 96-well plates were coated with termite extract (100 μg/mL) overnight at 4°C. After blocking for 2 hr, GCr allergic serum pools (1:10) were added to each well. Specific IgE binding was determined following sequential addition of biotinylated goat anti-human IgE (1:1000), HRP-conjugated streptavidin (1:10,000), and TMB substrate. (C) Dose response binding of human IgE in human serum pool (S1Cr) with the indicated amount of Cf termite extract.

To determine if the cross-reaction between termite and cockroach proteins could be clinically significant, human serum IgE from cockroach allergic patients was evaluated for recognition of termite proteins. In each of the 5 serum pools that were tested, there was recognition of termite proteins, and there was excellent overlap with the recognition of cockroach proteins using the same sera, especially for the S1Cr and P4 pools (Fig 4A and S5 Fig). A protein migrating just above the 52 kDa marker was observed in all 5 of the serum pools by immunoblot, and this signal was not present in five control samples from patients with allergy to sources other than cockroach (data not shown). Gel slices in the region overlapping with the 52 kDa reacting band were excised and the bands were subjected to trypsin digest and mass-spectrometric analysis to identify the cross-reacting protein. From this region of the gel peptides matching those predicted from *C*. *formosanus* actin (gb AGM32180.1, 30% protein coverage), three peptides from the *B*. *germanica* tropomyosin protein (UniProt Q9NG56, 13% protein coverage), and two peptides from the putative *C*. *formosanus* beta-tubulin protein (gb AGM32580.1, 9% protein coverage) were observed. In 3 of the pools (S1Cr, P2, and P4) there was a band migrating between the 31 and 38 kDa markers, suggestive of tropomyosin or possibly arginine kinase. This signal was not detected in absence of extract in ELISA (Fig 4B). A dose response of the termite extract with the S1Cr serum pool indicated that the half maximal detection limit was around 3μg/ml (Fig 4C).

**Fig 5:**
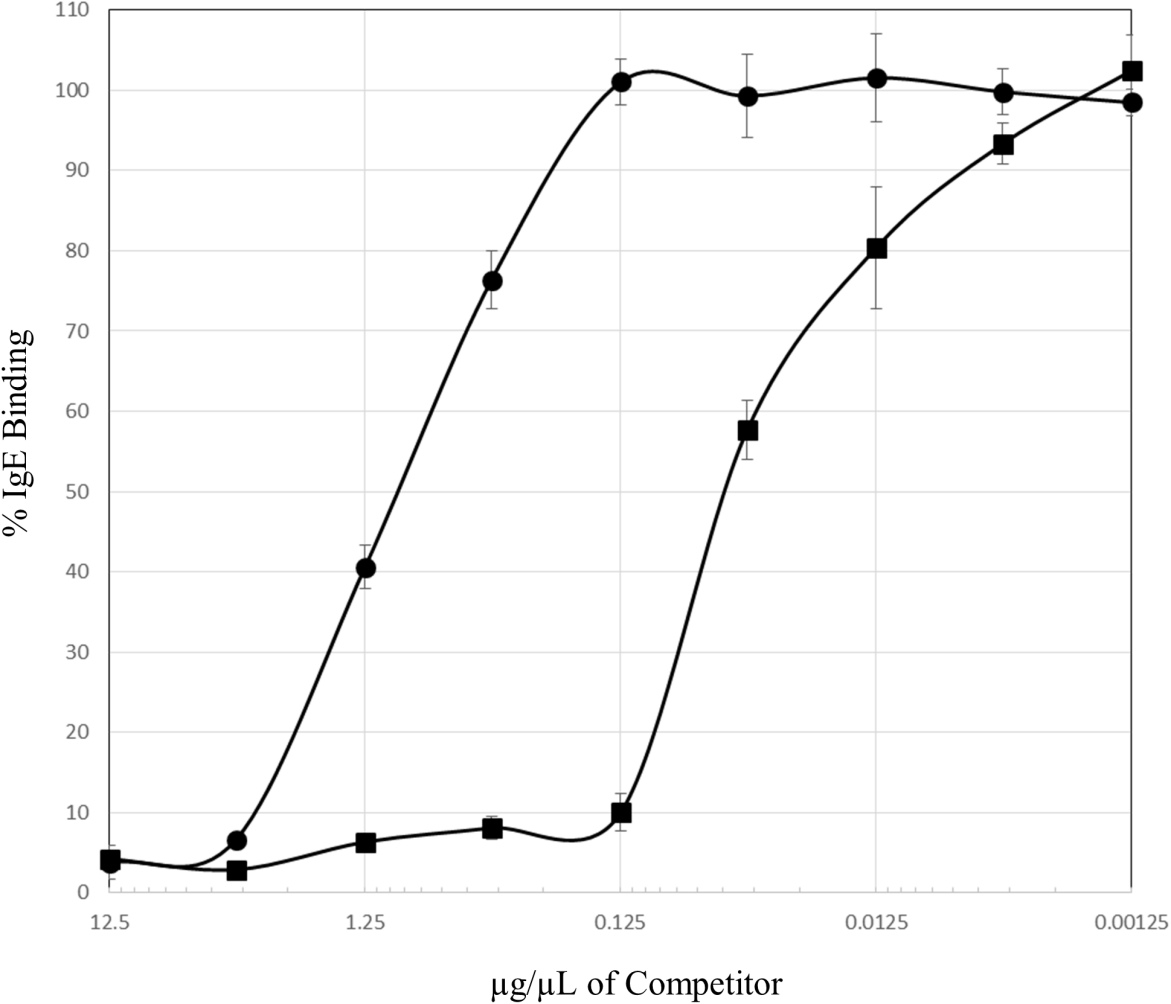
Termite proteins compete for IgE binding to cockroach allergens. Microtiter plate wells were coated with GCr extract (1 μg) probed with cockroach allergic patient sera (25 μL) that had been pre-incubated for 1 hour with 25 μL of serial half-log dilutions of either GCr or Cf extract in a competitive ELISA. Squares represent GCr extract and circles represent Cf termite extract. IgE binding was visualized by the sequential addition of biotinylated mouse anti-human IgE (1:1000) and IRDye680-conjugated streptavidin (1:5,000). Data points represent the mean of 3 tests and are shown with standard deviation included as error bars. The percentage of IgE binding is indicated on the y-axis and the concentration of GCr or Cf competitor is shown on the x-axis.

A competitive ELISA was used to test directly if Cf proteins could compete for IgE binding to cockroach allergens. At relatively higher concentrations the Cf extract was able to compete for IgE binding from a pool of cockroach allergic patient serum samples (Fig 5). The concentration of Cf extract required to inhibit 50% of the IgE binding was approximately 0.88 μg/μL whereas the GCr extract inhibited 50% of the IgE binding at an approximate concentration of 0.048 μg/μL. These data indicate the GCr extract is about 18 times more effective at competing for IgE binding in this assay.

## Discussion

Protein sequence homology and antibody cross-reactivity between some cockroach allergens and termite proteins was identified, and termite proteins could compete for IgE binding to cockroach allergens in a competitive ELISA. This research could have important consequences to the diagnostic and therapeutic allergy fields. Although the proteins from whole termite extracts did not compete as well as cockroach allergens for IgE binding, identifying and characterizing the cross-reacting termite homologues of important cockroach allergens could be very informative. The findings presented here do not allow firm conclusions on which specific termite proteins cross-react with cockroach allergens in a clinically relevant manner. However, the combined data using human IgE, anti-cockroach IgG antibodies and scFv fragments suggests that Cf termite protein homologs to Bla g 3 (hemocyanin), Bla g 4 (calycin), Bla g 7 (tropomyosin) and possibly others provides support for the continued analysis of purified native and recombinant termite proteins with IgE from cockroach allergic patients to directly establish clinical cross-reactivity. In support of this, phylogenetic analysis of the available Arthropda tropomyosin protein allergens indicates that at least one Cf tropomyosin protein (1264.m000365) is closely related to the German and American cockroach proteins and would be predicted to cross-react as it is more closely related than other cross-reacting tropomyosin proteins from shrimp (Lit v 1) and dust mite (Der f 10 and Der p 10) (S6 Fig). Similarly, both predicted Cf hemocyanin proteins (1481.m000238 and 1481.m000236) are more closely related to the Bla g 3 and Per a 3 proteins than the shrimp Lit v 2 protein (S7 Fig). Further, this type of analysis would predict that at least one of the Cf arginine kinase homologs (4552.m000111) would likely cross-react with the Bla g 9 and Per a 9 allergens as it is more closely related than the shrimp Lit v 2/Pen m 2 and dust mite Der f 20/Der p 20 proteins (S8 Fig). Continued research on potentially cross-reacting termite proteins could allow the development of hypoallergens, advances in epitope analysis, and the development of molecular chimeras with unique and informative epitopes.

This analysis has demonstrated that termite allergens cross-react with cockroach allergens, and as the analysis of the draft termite genome sequences improves, additional cross-reactive allergens that warrant laboratory and clinical investigation may be identified. There are no commercially available termite-specific allergen diagnostics tests. Whether the available cockroach (or dust mite) allergen diagnostic tests would accurately detect potential termite allergens is an important question that needs to be addressed. Termite-specific antibodies and other types of diagnostic tests would ensure accurate detection of termite proteins in infested areas.

The correlation between cockroach/dust mite allergens and increased asthma in inner city children demonstrates the medical importance of insect allergens. Treatment of asthma or insect allergies (with well-characterized allergens) is essential for effective treatment. For example, if treatment with cockroach extracts in asthma or other allergic disease has been unsuccessful, it may be that the presence of a related insect (such as termites) may also be a contributing factor to the disease. Termite extracts could generate a therapeutic avenue for individuals that do not respond to treatment with cockroach extracts because their allergy may, in fact, be due to sensitization to termite-specific allergens.

The potential exposure of humans to termite allergens is arguably different than that of cockroaches. Termites live within or close to their food source compared to the foraging activity of cockroaches. However, the potential of exposure remains, and termite colonies are made up of millions of insects, far more than most cockroach infestations. Dwellings with active dry wood termite infestations accumulate frass (termite droppings) which likely contain potential allergens. Mud tubes, used for travel inside termite-infested homes, may also provide a potential route of human exposure. Another potential exposure route is the alate swarming season when 10s of millions of reproductive forms of termites swarm for mating. Termite alates are attracted to light sources and most humans in swarming areas come in close contact with termites during this time period.

These findings may also have implications in the food allergy field. Alternate sources of protein could be useful in the future to help feed the increasing population. Entomophagy is not popular in the United States, but is common in other parts of the world and is an increasingly prevalent topic of discussion [38]. Research is needed to ensure the safety of additional food sources such as termites and other insects. For example, studies characterizing the processing and cross-reactivity of mealworm proteins indicate they can cross-react with dust mite and crustacean allergens and may therefore pose a risk to dust mite and crustacean sensitive individuals [39, 40]. Similarly, proteins from the field cricket (*Gryllus bimaculatus*) have been shown to cross-react with IgE from prawn allergic patients [41]. Termites are an excellent source of nutrition [42–44], and in some areas of the world they constitute a portion of the primate and human diet [45–48]. In the current study, some limited support for potential cross-reaction between cockroach and termite tropomyosin and arginine kinase proteins was provided, but continued research with purified proteins and recognition by IgE from additional clinically relevant samples is needed to clearly established cross-reactivity. Tropomyosin and arginine kinase are major shellfish allergens [49], and there is evidence that the cockroach proteins cross-react with shellfish proteins [50–54]. Thus, the results suggest that consumption of termites as food might also pose a risk for some food allergic individuals.

Unlike cockroaches, *C*. *formosanus* is mainly found underground, and this may limit exposure to termite proteins when they co-habitate with humans. While the results presented here clearly show termite proteins can cross-react with cockroach allergens, the behavioral differences between these insects makes it unclear what, if any, significance this cross-reactivity may have to allergic diseases such as asthma or other allergies. Previously, termites had not been considered as part of the household milieu that may sensitize individuals, but the results indicate that in areas with high termite populations these insects should also be considered as a potential source for allergen exposure. These results may have several implications for termite infested areas, such as the Gulf Coast states where *C*. *formosanus* has become a significant pest. Continued research to more clearly define cross-reaction between cockroach allergens and termite proteins will be useful, and future research and testing is needed to determine the clinical significance of these findings.

## Supporting information

S1 Fig Predicted *Coptotermes formosanus* termite proteins homologous to cockroach allergens. *Coptotermes formosanus* (Cf) termite protein and nucleotide sequences of predicted cockroach allergen homologs.
(DOC)

S2 Fig Uncropped immunoblots used in Fig 2A. (JPG)

S3 Fig Uncropped immunoblots used in Fig 2B. (JPG)

S4 Fig Uncropped immunoblots used in Fig 2C. (JPG)

S5 Fig Uncropped immunoblots used in Fig 4A. (JPG)

S6 Fig Phylogenetic tree of tropomyosin proteins. German and American cockroach tropomyosin proteins (Bla g 7 and Per a 7) along with 22 other allergenic tropomyosin proteins included in the IUIS website listed under Animalia Arthropoda were compared with 2 putative *C*. *formosanus* tropomyosin proteins (1264.m000365 and 4474.m000137) using the multiple alignment search with the BLASTP suite at ncbi.nlm.nih.gov. The data are represented using the phylogenetic distance tree option and the bar represents the number of amino acid changes per residue.
(PDF)

S7 Fig Phylogenetic tree of hexamerin and hemocyanin proteins. Hexamerin and hemocyanin proteins from 8 species, including the German cockroach Bla g 3 and the American cockroach Per a 3 allergens, were compared with 2 putative *C*. *formosanus* hemocyanin proteins (1481.m000238 and 1481.m000236) using the multiple alignment search with the BLASTP suite at ncbi.nlm.nih.gov. The data are represented using the phylogenetic distance tree option and the bar represents the number of amino acid changes per residue.
(PDF)

S8 Fig Phylogenetic tree of arginine kinase proteins. German and American cockroach arginine kinase proteins (Bla g 9 and Per a 9) along with 7 other allergenic arginine kinase proteins included in the IUIS website listed under Animalia Arthropoda were compared with 2 putative *C*. *formosanus* tropomyosin proteins (4552.m000111 and 1950.m000177) using the multiple alignment search with the BLASTP suite at ncbi.nlm.nih.gov. The data are represented using the phylogenetic distance tree option and the bar represents the number of amino acid changes per residue.
(PDF)
