# Supplementary material for: Cross-reaction between Formosan termite (Coptotermes formosanus) proteins and cockroach allergens
Source: PLoS One. 2017 Aug 2;12(8):e0182260. doi: 10.1371/journal.pone.0182260 (PMC5540505; doi:10.1371/journal.pone.0182260)
Supplement: S1 Fig — Coptotermes formosanus (Cf) termite protein and nucleotide sequences of predicted cockroach allergen homologs. (DOC) [file pone.0182260.s001.doc]

**S1 Fig. Predicted *Coptotermes formosanus* termite proteins homologous to cockroach allergens.** *Coptotermes formosanus* (Cf) termite protein and nucleotide sequences of predicted cockroach allergen homologs.

>1481.m000236 allergen Cr-PI, putative

MNTALVCATVVALFVCGAFSDHHIEKKMADKTLLMKQMNVLRLVHRLHQENIFKEQVDVG

NTYDIEAQISSYKNPKVVKDFISYYKKGMVKRWEPFSVYYKEHLKQAICLFDLFYFANDY

DTFYKTACWARDRVNPLMFWYSFTIAVLHREDTRDVIMPPPYEVYPYFFVDNEVIQKGYE

YWMMNVRNTPQHTHIIPMNYTMKNMENMLWYFTEDMGLNTYHMYYRNLYPSWFNVTEYGH

TFDRRGEMFLYVQHQVLARYCIERWANGMACVEPFVYNKALKTAYNPKLMYHSGQVMPPR

PSGMMVTNFDTFTIDDIKNYERRVSDAIDFGYVKDERLKVHSIYEDKKGIEYLGQMMEGT

YNSPHYYYYGSLFHLYRMMVGHMMDPLHKHGLAPSALEQPPTALRDPVYYQLSKRMYLLV

KKYKERLPRYTHEELLLEGVTVENVEVGKLYTYMENFEVSLGSTICVAKEEDMNDVNMHV

RMQRLNHKPFTYKIEVSSDKAVDAYVRVFLAPKHNHLGEEMDMNDRRHLFVEMDRFPYHV

QVGKTVIERNSHDSSIVAPTPDSYRTFWKKVTDAVEGKTQYYIDRSHNYCGFPKNLLLPK

GQKGGMAFTFYVIITPYVKQDEHDFEPYDYKSFSYCGVGHGRKYPDDKPLGFPFDRQIRS

KDFNTPNMFFKDVYIFHKKLDEVSTTTH

>1481.m000238 hexamerin subunit precursor (Bla g 3???)

MRTALLLVFLATGALAYPGTSPSQETRIIADKQFLQRQNDFLRLLVRIEQPNYYEDQVTL

GNSYDIEGNIKNYKHPQVVKQFLSAYKKGFLPRGVPYSPYYTTQSYETKLLFDLFYYAND

YDTFYKTAVWARDRINAGQFLYAFSVATFLREDLNDIVLPPPYEIYPYLFVDADVIQKAY

ETRMWDQKLTSQKTYVFPANYTVHTPEQVLTYFTEDVGLSTYYLYHYYNYPTFFNSTEYG

VHFDRRGEQFYYKIQQLFARYVLERLSYDLPEVQPFHYDKPFQTAYNPNLRYANGQEVPF

RPYEYSKRNLYNYNGQGYYYGNYYGGSNEYYTGNYYIGNYKPTYYYGYTNNYDYYYPEDI

KSYEGRIRDGIDFGFFFSEAGQVKYPLYDDYLKGINYLGDIIEGNGDTVNRRFYGALYPA

YRQLAGQSADPYNNYGLAPSALQNIFTALRDPANYQILKRVNYLFQRYKNYLPQYTYQEL

AYPGLKIENVEVGKLITYHDYFDIDLDNVVNVKVPEDGQYIDYRARQTRLNHKPFTYSID

VISDKATEAYVRVFLGPKYDYLGREYNINDRRHYFVEIDRFPYTIEAGKTTIQRNSRDSS

VVTPDYPSYRTLLRKVNDALDGKDQFYIDRTERYCGYPERLLLPRGKKGGQAYTFYVILT

PYVKQSEHDFEPYNYKSFSYCGVGFNNKYPDDKPLGFPFDRPIYGGDFYTPNSYFKDVVI

FHKKEEEVNTAITQ

>4541.m000139 glutathione S-transferase

MAPAYKLTYFNVKSLGEPIRFLLSYGGVEFEDCRFEDEEWPQVKPSTPFGKTPVLEVDGK

QTHQSAAICRYLAKQFGLNGSSDWEALEIDAIVDTFTDFRQLAFNERLQKHSLNVLTYKI

DQPLTWADIYFVGILDYLSVMAKEDLLEKYPKLKALKEHVLALPAIKAWVEKHPETQKFS

DVNKTTLYRVGRLCCKWAILESVHILRYIA

>57.m000588 allergen Bla g 6.0301, putative Troponin C (Coptotermes Formosanus AGM32406.1)

MSEKLPNVYGDAFVFPRFFTSFFAAIKIAGHGRRNQRKQLIQMLKKAFDAFDREKSGSIS

TNMVEEILRLMGQPFNRNTLEELIDEVDADKSGRLEFDEFITLAAKFIIEEDAEAMEKEL

REAFRLYDKEGNGYIPTSCLREILRELDDQLTSDELDMMIEEIDSDGSGTVDFDEFMEMM

TG

>14777.m000067 troponin C type IIa family protein

MDDDDQKMQIMRKAFQMFDTTKCGFIETLKISTILNTMGQLFDDGELQNLIEETDPEGTG

KVNFDGFCRIAAHFLEEEDSEAMQEELKEAFRLYDREGNGYITTKTLREILQALDDKLGP

EDLDGIIAEIDTDGSGTVDFDEFMEMMTGE

>4607.m000136 EF hand domain-containing protein

MHAHNRVGNPKIILFSVLKKAFDAFDREKNGCISTEMVGTILEMLGHRLDDDMLAEIIAE

VDADGSGELEFEEFVTLAARFLVEEDAEAMQQELREAFRLYDKEDFERSYCLRKVEFYQP

MTHGNIARTKASTTEIWKPQTRS

>809.m000404 calmodulin, putative

MADQLTEEQIAEFKEAFSLFDKDGDGTITTKELGTVMRSLGQNPTEAELQDMINEVDADG

NGTIDFPEFLTMMARKMKDTDSEEEIREAFRVFDKDGNGFISAAELRHVMTNLGEKLTDE

EVDEMIREADIDGDGQVNYEGRALWQPWACPTISHSYXGDLGLQNSS

>4474.m000137 tropomyosin 2 family protein

MDAIKKKMQAMKMEKDSAMDKADSCEAQAKDANLKADKILEDVRDLEKRRLQLEQDLETT

KKNLEQANKDLEAKEKALQAAESEMAALNRKVQLIEEDLERSEERSGTALTKLHEASEAA

DEASRMCKVFENRSLQDEERMDQLTNQLKEARLLAEDADGKSDEVTQKLANVEDELEVAE

DRVKTGESKIMELEEELKVVGNSLKSLEVSEEKANQRVEEYKRQIKSLTVKLKEAEARAE

FAEKTVKKLQKEVDRLEDELGINKDRYKSLADEMDSTFAELAGY

>1264.m000365 tropomyosin, putative

MIGEIAESEVAALNRRIQLLEEDLERSEERLATATAKLAEASQAADESERIRKALENRTN

MEDDRVAILEAQLSQAKLIAEEADKKYEEHACAHTDTHITVRSLVSHSKIVQPHRQSNGS

CWHQLWLHRARKILESKGLADEERMDALENQLKEARFMAEEADKKYDEVARKLVLMEQDL

ERAEERAEQSDSKIVELEEELRVVGNNLKSLEVSEEKANLREEEYKQQIKTLTTRLKEAE

ARAEFAERSVQKLQKEVDRLEDDLVAEKEKNKLLQEEMEATLHDIQNM

>4958.m000213 myosin light chain

MADXXXXXXXXXKDDAPAADAAPAAAETPEPASAAAEPNRQSSRGSKKVRRTGSNVFSMF

SQRQVAEFKEAFQLMDQDKDGIISKNDLRATFDQLGRLPSDKELDEMVNEAPGPINFTQL

LTLFAGRMSGGSDDDDVVIAAFKSFDDEGKIDSERLRHALMTWGDKFTGGEVDDAYDNFE

IDDKGFINTAKLIQLLTASPEEEEEEGA

>5766.m000242 myosin II regulatory light chain

MASRKTTSRRTTTKKRAQRATSNVFAMFDQAQIQEFKEAFNMIDQNRDGFVDKEDLHDML

ASLGKNPADDYLEGMMNEAPGPINFTMFLTLFGERLQGTDPEDVIKNAFGCFDEENKGVI

NEERLRELLTSMGDRFTDEEVDEMYREAPIKHGMFDYIEFTRILKHGAKDKDDQ

>3431.m000139 1,4-alpha-D-glucan glucanohydrolase precursor

MVLLHLIWXLFSVANIQAQKDPNTAEGRSAIVHLFEWKFSDIADECERFLGXKGFAGVQV

SPVHENLVIPSRPWYERYQVISYNIISRSGDEAAFRDMVNRCNAVGVRIYVDVVFNHMTG

DQPNAHGVNGTTANPSEKLYPGVPYDTNEFHWSCSINNYQDAGNVRNCELVGLHDLNQGL

ESVRQKIVEFLNXLIDAGIAGXRVDAAKHMWPADLENIYNQLHNLNTTHGFAPGSRPFIY

QEVIDLGGEAISSSEYTAFGRVTEFKYGAELGNAFRGNNAIKWLINFGQPWGFVPDGDAL

VFVDNHDNQRGDGGTGGNILTYKTSKLYKMAVAFMLAHPYGYPRIMSSFFFNDTNQGPPH

DDSDNIXSPTINXDGTCGXGWVCEHRWRQIYNMVAFRNVVAGTXVNDWWDNGNQLIAFCR

GXKGFIAFNDQYDTDFKVTLQTCLLPGNYCDIISGSKINGTCTGKTVNVASDGXAYIQIL

HXEEDGVLAIHELVRVYSDFII

>13638.m000105 alpha-amylase

MWPADLENIYSRLDNLNTEHGFAPGSRPFIYQEVIDLGGEAISSSEYTAFGRVTEFKYGA

ELGNAFRGNNAIKWLVNFGQPWGFVPDGDAFVFVDNHDNQRGHGAGGESILTYKTPKEYK

MAVAFMLSYPFGYPRIMSSFFFDDTNQGPPHDDNENILSPTINSDGTCGXGWVCEHRWRQ

IYNMVAFSNVVAGTGVNDWWDNGNQLIAFCRGGKGFIAFNDQYDTDFKVTLQTCLPAGDY

CDXISGSKIDGTCTGKTVNVGSDGTAYXEILGDEEDGVLAIHAE

>27662.m000053 alpha-amylase

MVLLHLIWFLFSVANIQAQKDPNTAEGRSAIVHLFEWKFSDIADECERFLGXKGFAGXQV

SPVHENLVIPSRPWYERYQVVSYKIISRSGDEAAFREMVNRCNAVGVRIYVDVVFNHMTG

DQEDAHGVGGTAAYPSQKLYPGVPYESSDFHSTCSVENYQDAENVRNCELVGLHDLNQGS

DSVRQKIVEFLNRLIDAGIAGIR

>350.m000777 prophenoloxidase

MLKSATRFEPSFTSLRSSESQKFQFTMSRPAQPQDILLMFDXPTEPIFMPKGDNNNVXFD

VPASLLDNRINADAGLVNRLGAGEADDHVQVKPIALPDLTIPQSLSRDQNFSLFIPLHRK

AAGRLIEILMGMRNIDDLVSMSAACRERVNPYLFNYALSVXLLHRPDTRNLRIPPLFEAF

PDKFVDGSIFNKARQESEIFLSGSRQPLEIPIDYTASDLDIEHRVAYFREDLGINLHHWH

WHLVYPFTGPRNLVDKDRRGELFYYMHQQIISRYNFERLSNKLARVTRFLNWREPMPEGY

FPKLDSLVSSRVWPPRHANALIRDVNREVDQLKVDIADCERWRDRFYDAIHQGTVLMPNG

NRELLTEEKGIDVLGNLMEASILSVNMNVYGDLHNMGHVLISLCHDPDGSNLETFSTIGD

PATAMRDPMFYRLHSFVDDIFQEHKSTLPRYDVNRLGYNGITIRSVDVETSGAPKNEFRT

FWKKDDVDLSRGIDFTPRGNVFARFTHLQHTDFIYKIQADNSTNSPKVGTVRIFLAPKFD

ERGVNMLFRDQRLMFIELDKFTVTLKPKSNTIQRKSTDSSLTIPFERTFRDLQSNRPTAG

AALEQFNFCGCGWPQHMLIPKGTPEGMPCELFVMISSYDDDKINQNIEGACADAASFCGI

RDKLYPDKRSMGYPFDRMPRDGVNTLTDFLTPNMFVQDVSIKFTDRTVGVQGGDEPNRLT

>11697.m000092 pro-phenol oxidase subunit 2

MRNINDLVSMSAACRERMNPYLFNYALSVALLHLPHTRDLQIPQLVKCFPAKFVDASILI

KARQESEIFLSGSRQPLEIPVDYTASDLDIEHRVAYFREDLGINLHHWHWHLVYPYTGPH

ELVDKDCRGELFYYMHQQIIARYNFERLSNKLKRVTRFLNWREPMPHGYFPKLDCRLYPP

RHDDVVLRDLNRDKQKVDIVDCERWTDRLYDAIHQGSVLMPNGNCELLTEEKGIDVLGNL

MEASILSVNMKVYGSLHNMGHDIISFCHDPDGSHLEEXGVIGDTATAMRDPMFYRLHAFV

DDIFQEYKSTLPRYDVNRLGYSGITIRSVDVETPGAPKNEFRTFWKKDVVDVSKGTDFTP

RGKVHVCFTHLQHTDFIYKIQ

>1950.m000177 arginine kinase

VLIKFHIWSCLCCAGLENHDSGVGIYAPDAEAYSVFADLFDPIIEDYHGGFKKTDKHPPK

DWGDVDTLGNLDPAGEYIISTRVRCGRSLEGYPFNPCLTEAQYKEMEEKVSSTLSGLEGE

LKGQFYPLTGMTKEVQQKLIDDHFLFKEGDRFLQAANACRFWPTGRGIYHNDAKTFLVWC

NEEDHLRIISMQMGGDLGQVYRRLVTAVNDIEKRIPFSHDDRLGFLTFCPTNLGTTVRAS

VHIKVPKLAADRAKLEEVAGKYNLQVRGTRGEHTEAEGGVYDISNKRRMGLTEYEAVKEM

HDGIAELIKLESSL

>1322.m000276 ATP:guanido phosphotransferase, C-terminal catalytic domain-containing protein

MLHEAVSRGQLGDMETLLQEEKSKRMALCKDSAGVPLLQKAMYYNHLDIVKWLVETYPIT

VSTKDREGRTALFYCCVLKDPKETWELLEAAGADPTVTDSQSRIAAYYIDHPNEIELPDS

RDTSGSFRRFTSGKDGLVVTRANIRIWIHDRDLGRLQQMLWEGHGGKLRVETSNNPRVKR

FLDAVPYIMGVIKDVHGAVVNNDEETFQKRTADPVPNQIFMSKDQNGLNPLHKAAGLGRE

GMVESILQRNPNAATXTDNEGRLPLHYAAAVRDGGHIYNLLVEAGAEENALDNRGKPPSY

YRTKPAELDLKNLQVIPDAPRTATIFPPAWDWRLLNNMTYDFGPIPIDNDLKYDGDNLPE

RMKAMPAAVAVGISHPNQRTPTPISVNKEAERPDDEDEDEGVGEEGDHDEEWERQDNDME

AAGEDREEDAGMGEDEEEETVPEREENGEVTGEDTNAAPAEDDEEVRQLVEAGNMEQLAN

LVLNGEGHRLIRQKSDDPELQGFLNNVPAYMAKIRAVHEAAQDGRLRDLQAALDRRKFAI

ARDGSNAMGTTPLHVATLFGHTAIIRYLGGRFSETLQARDNSDRTPLHYAATMADNGHYY

NLLLNLGADPALKDNLGNTAEYYLKNLGILTHQDLLEEYGASQQVLNGMLEDKVPNDTVS

ARRDIDDPEILETLERCYELVREETSTNGQLMSGTLLQRYLKRPVFEKLKKRLTRMDHNL

LDVIWPGAKEVPEDNAGDIDEELEEVIQRNGGVIAPDYESYTVFAELLLPLIKDLHGLLV

SYDLNPQPKSLFFLAEEDEDENDEDEIKKKKGHIGEIIIDHSGKHVKSGRIECCRNVGEY

QLPSGLSFAHLEALERDLVNSLEDCAGEETTDNNGDEEDKSYYPMAEVLEEKSKIXEELE

ALNLMILLTDGHENEDNLLHGQHWPHGRGVFITKDKNMAIWINVQEHLRIVSSTPKEKPS

SLGEAYDXXAHLMMDLETMFEFKRDPVLGYLTACPXALGNSLHIYLTAHLPKLGQEEDEL

RYLCSVRGLNIKRKPEGQDKFNIWNQQSLSITEHQTLNVFSTAASNIIQLESNHSKKPKK

>4552.m000111 arginine kinase

MGGCASKDKAKTEATGANGSNTPAPATNEDDVKSEDTMVDQAVLDKLEAGYAKLAASDSK

SLLKKFLTKEVFDNLKTKKTPSFGSSLLDVIQSGKLKC

>2550.m000162 MPA3 allergen, putative

AATLSRRPRLDGRIVGGEDANIXDYPYQVSFEYENRHICGGSIISAXWVVTAAHCVDGLP

ASKVQFRAGSSNRETGGTLHPASNITAHPHYDYYTVDFDVAVVRVSTAFNIGTGVQPIPL

TTSEPQPGQIAVVTGWGTLASGSHTLPRRLQLVNVPIVSREECSXXYEXYGGITENMICX

AXPGGGKDACQGDSGGPLAVDGTLAGIVSWGAGCAXPSXXGVYSNVAVLRSFIXEQTGVN

>2550.m000161 MPA3 allergen, putative

ANLPLRRPRLDGRIIGGSAVGIEDFPYQVSIEYAGSHMCGGAIIGPVWVVTAAHCVDGVS

ASNLRVRAGSIIWGTGGTLHLVSQIIVNPHYDYYTFDYDVALIAVSVPFFYGISVQPIPL

AVQKPGPGVTGVVSGWRNSSTGSSPRLQAISVIIASDSLCNAAYAQHGVITANTICASAN

GGGTGACQGNSGSPLAVGGELAGLVSWGIGCAGFNYLDVFSNIARVRSFIISNTGII

>26042.m000070 MPA3 allergen, putative

MLRCLVLASLIACSLGAATLSRRPRLDGRIADGELAIIEDYPHQISLEYKNRHICGGAII

SASWVVTAGNCVDRVPPSQVGIRAGSSYRETGGTLHPASYIVLNPQYDYYTLDFDVAVIR

VSPAFNMGREVQPIPLTTSELEAGEVGRVSGWGTLSKDNETLPLQLHYAIGVTVSREECN

KAYEKFGGITENMICAAFLNMGPCFGDSGDALSVDHILIGIVSWSFECGESTHPTVYSNV

AVLRSFITEETGVN

>28569.m000019 MPA3 allergen, putative

MLWAYYQPPCEPSAYAQIYIETYCTATSFRTNRASTSSVRFRAGSSTRGTGGTLHPAAQL

VLNQQYDYYTVDYDIAVARVSVSFTYGTGVQPISLTSSEPAAGALAVVSGWGTLSSGSSS

LPXQLQAVEVNIISSTSCNNXYSEYGGITARMICAGVSGGGKDSCQGDSGGPLVVSGSLV

GIVSWGAGCAEADYPGVYSNVAVLRSFVTDETGVA

>40061.m000014 MPA3 allergen, putative

MPPYEPSAYAQIYIETYCTATSFRTNRASTSYVRFRAGSSLRSTGGTLHPAAQLVLNPDY

DYYTIDYDIAVARVSVPFTYGTEVQPIGLARTEPPAGTLAIVSGWGTLSSGSNSLPSQLQ

AVEVDIISRSVCNDAYAIYDGITARMICAGVVGGGKDACQGDSGGPLVAEGSLVGIVSWG

EGCGEADYPGVYSNVANLRSFVTNATGVA

>4240.m000122 MPA3 allergen family protein

MPQNLQVVMVPFITYDTCRDIYSNYTEGSIEPGMNCAGFLQGGKDACGGDSGGPLVCGGL

LTGVVSWGEGCALPNFPGVYADVAYYKEWIERQLLESKGEFFLRN

>31122.m000014 MPA3 allergen, putative

VSFAFTYGTRVQPLKLPPYEDPEFSVLDVSGWGTLSSGRCLLPSQLQAVTLLHSPRSECN

SVYAAYGGITENMICPCWPGEVKGACQGDSGGRLGGAEFLIGIVSWGAGCAEPNYPGVGS

NVAKLKDFISEQTGVN

>contig_55012

GCACATTCCGGTTCAGTTCCATCAAACAGACTGGCTGTACCAAACATCTTGCCTCTTGCCTTAAATATGCTTTGGACAAG

ATAAATCTTCTTCTCTCACAGTTTTAATTTTGTSCTGTTCRTAGTCGTGATGGATGTTACTACCTGCAAGCTGGTGTTGT

GTCATACGCTGAACATTTCACAAGTTGAATTACTGTGTGCCTCATATGCATGTGTGTCTATACTGGTATTTTATTTTATC

TTTTTAAATTATCCATTGAATATTGTAAAATACTGCCAAAATCTTTGCATTTATGCCCATGCAACGATTAGCAAATATCT

TCCCGTTTAACAAGGACTGAACAAATTTTATATTATACATACATTCCGTGCACTGTGCTTGCAATTATTTATACATAAAT

ACATATTTATGTTTATATATAAATGACTGTAAGAATTGGACGACTTTACCTCTCATTACTTCCTTGGGTGGCTTGCCATA

GTTGACATTCCAAGGGTAATCCTTCTACTTGTCTAGTTTAATCCATCAGCCTGATGGGTGGTTTTGCATTTTGTGTGGCG

CAATTACCAGTCCCTTACACAATTCCCAACCTGGAGGACCCGAGGCTTAATTTAGGGTTACCTTGCCCTAGGTGAAATGC

TTCATCTGTCAGGGCTTATTCCACTAGATGAATTTCCCAGTTTCAAGGCACATAATGGCAGTGTATTCCAAAATATATCA

ATAGTATTGCTAAACACAGTTTTCTGTAAGTTGGAAGAATGGTTTGGTTTGCCCCTGTGTTGGGCGTTGACCAAAACTGA

AAGATAGGTTAAAATGTGATAACTTCATTATGTTAACATTAGGCCCCCGATTTTTAGAGATTCATTTTCGTCTTTTCCCG

CCAGAATATTTTAATGTATATGAATTTAAATTCTGAATACAATTTGTAGGACACATTTTTCCCTGCCCACGATTTTTTTG

TTGTGTTATGTGGTCAGCATTTTAGATAACTGTTTTTTAAAAGCCTTGAGAAGTCCACTTTTAGATACACAATTTTAACA

CTTCTGGTGCAATTGCTGTATCCTGCAGATCTGAGTGTTCAGCATTTTGCTACTACAAACACCAGCGCAGTATCATAGAT

GTGATTATTGTAATTGTAAATATCATGTTTTAATATCAGTGAATTCTTTTACATTGATAAATTGCGTATACTGTTTTCAA

CAAACTGTATATAACTGATGTATGGACCTGAAGTTAGAACCACACAGCATGTTGAGTTAGCATCACTCTGTATGATACGT

AACTTTACACACTGCAATTAGCAGCTGCAAGAGAAGTTAAGTAAGTAAGGTTTAAGGTACATATCTTAATCAGTAAAACT

GTAACACTGGTGTTTGTTTTGCTATGAACCTCAGTGAGGTATACTGTAGACTAATTTTCAGATCTGAAAGATTAGGAATG

GATGAAGTGTTACTTGTCCATCCACTGTTCATAAATTAACTGTTTACTCAAAATGGATTAAAAATATGTGACTTACTGGC

AGCACAGTCACTTAGCCTCAGTATGATAAAGATCTGTCATGGGCTTATAAAAGTACCTGCTTTGTCTTTCATTTTTACCC

TATTGGATCGGTACTGTAGATGGTGCTGCCGAGTTTGTGGTCTTTCGGCAAGTTTTGGTATTTGATGATTGATTACAATC

GAATGTTTCTGGCAAATAACAGTCTATTAAAGTTATAAACTACCTTGTTACTTCATTTTCAGCTTGCTGACACTAATGAG

GAAAGTGATGCTATTGTATTGTCATGGCTACGCATGTGGCCCATGCAAACTTGTGCTATGGCAGCAGTGGTATCGGAGGT

GATAAAATGCATTCTATGAATTAAAAAAAAAATAAAAAAAAAGTCATGAAAGCTGACAACCCTCTGCCTCTTCAAAAGCA

TGGTAACTGATCAAAACATTCAAATGCACGTACATATAGTATAGTTTTTCCTTCAAGTGTAGTAATTTTGGGACAGGTGG

AAACCTTTCAGGATCTTGAATTTTGTGGCTTGTGATTTTATGAAACAGATATAAATGAATGGAGTGCTGTACTGAAATTG

ATTCTACCTATTTTACTGTGCACTTCTCCACATGTAGTTTCCCTTGATCATATATCGTATCTACTGCTGACAAGAAATCT

TACCATAGATATAATGCTACCTGCAGCCAGCAAATTATTTTGTGTATAGTTACTCATCCCATTGAAAGACATTAGAGTTG

TAGATTTTAATGAGATGTACATTACTGTTGTTTTGGAAGCCAGGACAGCATAGTTGGTATACTAAGCCAGGGCTGCACAA

ATCCCAGGTGCCAGTTATCATGGCGACTAAATTCTGTGTGGTGGTACCTGATATATGTAGGTCCTCAGTACGAAACTTGC

TTCATGTCACGCTTCTGCCAACTACAATTTTGAGTTTGCTCCTAGATTTTTGGAAAATCTGTGAGCCCCTGGAATAGGCT

ATAGGCTGGATGCCCAGGGGATCACGGTCTAGTACCTCGAAGTGGCAAGAGATTTATCTCTACTCCAAAGCATTCAGACC

AGCTCTGGGAATCACTCAGCCTCTTATTCAATGGGAAATGACGGACCTTCCTCAGAGGTAGAGCAGCCAGGGCATGAAGC

TGGGAATTCACGGCCATCTCTGCCAAGGCCAAGAATAAATGCAGCTAATACACCTCCAGGTCATGTGTAGTGCCTATGCA

CAGTCACAAGTACACGGTCCCATTATAGCAATTTATTAATTTTCCCTGCCCCTCCATCCCCCATTTTTGTTTTTTGTTTT

TTTTTTAGAAGTTCAATATTTCATGTTCTTCACTTTGGAAATTAAATGTAAACAGGATATAATGACAGCATTAATGCAAA

TGCCAATAGTGTACCATACAATTAGGTATACAGAACAAGTAAAGGAGTTATGGTAACTACGAGAAAGATATCTAAAATAG

TAATCACGTACATTATGCACCTAAAATACAAACACACACTTTATAGAAACCAAGCACTGGAATGGTTGATCATCTGTGGT

GTGCACCAGTGATGGTGAGTAGGGTTTGCACTAGCACTGTGCAGACTGAGACTTCTCAGGCACTAGTCTTATAAGAATGC

TGTTCAAAGACTGTCAAAAGAATGTATTCACAAACCACTTGTGTTGTCACCTGACTGGTGAGTTATGCACTGTGCCAGTC

TTTCAACTCACAGAAAATTAACAGCAATAATGAAATGGTTAAACATATTCATGTTTCAGCTGTCTGCACAAGTTTCACAT

TCTCAGTTTTCCAGTACTGCAATGCACACTCACACAAATGGTTGTTTTTGTTAACACGGGGTTTTCACTGAGATGTGTGT

AACAATAATAAATGACAGTGCCATCTACCGGCATTACACAGGGCTCAATATTGTGTTGCTGATACTTGTACATGTTTTAT

GGATGTATGAAAGTGCATAAATGTTTGAGAAGACTTCTTGTTTCTGCCTAGGCAATTTTGTTTTTTCCCTGGTTCAAGAC

ACCAGGACTGCACTGCATAAACTAAAAAGTGGAAAAATGCCTGCCTACCACAATTGATGGAGCTTGGCTAACATAGTTGA

CAGAGTGACTAGGCTAAGGACTAGATGTCTCGGGAACTGTTCCGCTTCCTGGCAAGGGCATGTGATTTATCACTTCACTG

AAGTGCGCAGCATGGCCTGGGGGCCCACACAGTCTCCTTCCTAGGGGTACAGCATTGGGGTGTATGAGTATTAGTTATCA

GCATTCCAAGGGAGTGTAGTGCCTTCATCCTGACATCCTGACTCTTTGGAAAGATGGAGAATATTTATGTGAAGGTTACA

AATCCTGTCTTCTGAGAAAATCACGTTGTGTGTGCGCGTGCATGCGTGTGGTGCAGCAGACACTAGAACATAGCCTGATC

CAGTTTCCACTTTGAAAATTTAGCAGTTCCATTCTCTACTTATGGCTTTCACATAACCCCTCCATTAATGCTACCTTACA

TAGGAACTGGCTTCTAAAACACATTATTGAAGAAAAGATAGAGGGAATGGTAGAAGTGACAGGAAGACAAGCAATAAGAT

GTAAGCAGCTACAGGATGACCTTAAGGAAATGACAGGACACTGGAAATTGAAAGAGGAAGCACCAGAGCACACTCTATGG

AGAACTTGCTATGGAAGAGGATATAGACTTTTGTAAGACTGACAACAGAATGAATGTAGTTTGACTGAAGCACAGTTACC

CCACCTCAGTCACTGATTTCTACCATCTTTAGGCTCTATGTATAAATACTGCTGACATTTAGTGTTAACAGTTATTGTGT

ATGGATGGATGGATGGATGATGATAACAGAGTTTGTAAAGTGCAATAACTCCGGGTGGGGGTAGTGCATCATATTTTTAT

TTCATATATTATATTTTATCTTTATTTTACAAGTAAAAACACATACAATCTTACTTTCCTGCAGATGGGCATCTTCTTTC

AAAGTTCCAACGTTACTTTGAGAGTAACGGCTTTAATCTCTTTTTCTAATGCGGTGACAGGCCAAAGAGTGCCCTAAGGA

GTTTGATGATGCGACCCACATCAATGCCCTTTTCTCGTAGGCTCTTGAGCAGTCTCTGGTATTCTGGCAAGGCTCGCAGC

GTCTCAACAATGTTCTGTGAGACAGAGTAATGCATTCATCACAGGAATTCACACAGAACTGTACACACTGTACACAGGGG

ACATAAAAAAATTTGAAACAATCTTTACAGCAAATTCAGGGGACTGAATGGCTTTAGCCACAGCCTGGAAGTCTTCGCTG

GTTTCCAGTTTTCAGTCAAAGAGGGCTGTCAATTCTTCGAGAGGAAGAACGGCAACCACATCATCGATGAGTCCGTTAAT

ACCAACACTCCTGCGAGTGTTCTTCTTGGGTTCGAATGGGGGCAAGCCAAGGATGTCATGAATCTGACTGATGATCGCAT

AAACATCAACACTGCCGTCACTGATGAATCTCACAAACTAGAAAAGAGCAATTCAGCGACCCATTGAAACTGAACAAACA

TTTTCTCTGTCAGAATGAGACTTAAAGAAAGTAAACATGAAGACACTCATGTCTTTAAATTCCCTTAGGTCTTGAACAGT

TCTAAGAATCTTGAGGAATTCTTCAGACTGGATGTAAATAACAAATTCCTGCACTTCTTTGTCATTGGATAGGTAGTCCA

CGGCAATTTCGAGGACCCTGTCAGTTGGGACAAGTGCCAGGAAGTCATTCAGATCATCCTGGAGGCTGCATGTTCGTGTG

TCAGAGATATCACACTTCTCCAACTAAGTTTATTTCTTKCATTCTGGGATTATGACAGTTGTAGTAAATTGACAGCTGCA

GATGAAACTCAGACCTTTTATAGGCACTGTTACTTTAAAGACCACCTCTATGTTGTATTTAGGATACTTACTGTGCACAA

ATATGTCAGTTTCTAAAATTATTCTCAGTCAATCTGGCCACATTTTTCATATTGTCTCAACACATATCCCAAAATTGTAA

TCTCTTGGTGGAAGACCAGATGTCACTGTTGACTACTTATCACTCAAGCTTAGTATTCAGGAGGTCCTGGGTTCAGATCT

CGGCCCAGAGCCTGGCCATCTTGACTGAAAGTTACTGGTATAGTACTCCAAGTTAAGCCCCTACTGGTTCCTTCCACATG

ATAAGGTATGACTGCTTGGCACTTGGAATAATTTATCTATGTGTTATTTATAATTGCTTAAGTTGACTCTGGTTATGAAT

TCCAAAGAAGGCAGAGTCACACTTTCCTCCCTGTATGAAAGAACTGAAACACAGTTGAGGGCTTTAGAAACGCTGGGAGT

GGCTACTGACAAGTACGCAGCCATGTTGTTCCCACTGGTAGAGTCCTGCCTACCAGAAGAGGTATTGAGAGCCTGGCAGA

GGCATAGTAATGGCAGTTTAGTTCAACAGAATGGCCAGACTGGACTTGATAGTCTTACGAGCTTCTTGAAGAATGAAGTA

GAGAGCGAGGAGAGGATTACTATGGCAATCCAGGGTTTTAGTTTTGGAAACAATGTTAGGGGCATGAAACAGCAAAAGAT

GGAATCCAGCAGTTCTAACAAAAACGTTGTTCCCACCACAGCTGACCTTGGTAATTGTAAACCCAGTAAAATGGCAAAAA

TGGCATGTGTGTTCTGCAAGGGGTCACATTCCAGTGATGCCTGCTTTAAGGCAGACAAAATGAGCACTGAGGAGAAACGG

GACATCACAAACAGAAAAAAATGTTGCTTTGCATGTCTTAAAACTGGACACATTAGGAGAAGTTGTAGAGCCGCCCTGAA

ATGTACATTTTGTGAGCGAAAGCATGTACCAGTGATGTGTCCTAAGGTTGAAAAGGCATCTGAAGCAAAGGTAGAGCCAG

TGGTAGAGAGTTCCTTGTCCAACATAAACTATAGTCAGGTTTTCTTGCAAACTATTATGGTAAAACTAAGAGGGGCACAA

GAAAGGAAGATAAGGGTACTGCTCGATACTGGATCTCTGCGTTCGTACATTAAGAGAGACGTGGCTCAATGCATGCAGTA

TAAGCCTACGGGCGAAGAGGAGTTGATTCACGGATTATTCTCTGTCAGAATGAGACTTAAAGAAAGTAAACATGAAGACA

CTCATGTCTTTAAATTCCCTTAGGTCTTCAACAGTTCTAAGAATCTTGAGGAATTCTTCAGACTGGATATTTTGAAGTAG

GTAATGCCAGAAAGACAGGCATGTGAACTTGTGAGGCCTGAACAACACTTGCATGCTTGTCAATAATAATCCTTGTGGCA

ACTGGACTGCACTGTTCTGTATCACTCAGAAATTCATTGCACTTTGAATATTCCTATGTGTTTCAGCACACTCTGTGGTA

CACCCAATTATATGGCTCCAGAAATCCTAAAGAGGCAGGGACATAGCTTTTTTAGTAGACATCTGGGCAATCGGCTGTAT

TTTGTACGTACAATAATGTTAAGTATATTGCATGTACTTGATTTTAGAATTCTGAATTTGTGTTCACTTCATAGGCCTAA

ACCTAGGCTGCAGAATGTGGCACTGCAATTAATACAAACATAGAGCAATAAACCGCTCATTTTTGGAGTTATTTGCTAAC

ATCAGTGTATAAGGACACACACAGCAAAAAAATATGCCAAATATATACTGTATTATCTTCATAAATGTTTACAATACTTA

AGAATTTTTTTTAATGTTAAACCTGCTGGTAAGTAAAGTTACCGCTAGGCTTTAAAAGGTTAAGATGCAACTTCTCTTTT

TGGTGCGCTTCAGTACAAAATTGTGAAATGATTCCCTTTTTTAGGTACACATAGTTGATTGGAAAGCCGCCATTCCAGGC

TAATTCAATAGAGAAAACATATTCTCGGATAAAGAAGTGTAAATATAAGATACAACCATCTTCACAGATATCACATTCTG

CAATTAAGATCATCCAAGCAACACTCCAGCCAGATCCCAAATGCAGGCCAAAGGTAGAAGAACTTCTGAACAGCGAATTC

TTAACATCTGGTATGTATTTGTGCAATTATTTATACTTGTAAGGAAAATGTGACTTCTATAAATGGACACAAGTGTCTTG

TGGTGAGTCATGTGGATGTTTAGAACCTGAGTACAGTCAACCCTCACTATCTGCTGTCTCACTTTGTGATTTTTCTCTAT

AGCAACTCACAGATATTAATACTCAAAATTTGTTACCTGCCTCAAATCCCCACTATTGTGACTCTGCATCTGAAAGAATT

TCATTATTTTTCCCATTTACAATAATAGGAAATACAGTCCTGGTATCTGATTTTTTGCTATCAGACCCGTTTTCAGGGAC

AGATTAGAGATGGAAAATGAGGGTTAAATGTGCTTATAAATCAAACAAAAATTGCATGGCACATTGCATATACCAGCCTT

ATGAAACTAACATTAAAAGAGTCAAACCTTATTGAACAACAGGCTGCTTGTACGTACATTTACTCCTGCTGTACTACTGG

TGCTAGGGCCACCCAGCCTCCTGTCCAAGTACAGTAAAGTCTGAAGGTTTGGAATTTTGACCATTTTTATCACTATTGTT

TTGATATTTTGTGACTGGTCCCAACAAATGAGACTGAGGCCACATGGGAGGCAAGTGAGCTTCCCCTACTCTCCTGCTAC

TGTGCATGTCACCCTCAGCTCAGTCAACCCCCATCTCATTAAGTGAGGTTTAGAGCTTTCGTGCTTTAGGGAGTGAGTCC

CTCCTTTAGTGATTTAGTGTTGTAGAACAATAGTGAAATAATTTAGTGTGTTTTATTCTTCCAAAATGTGTTTGCATTTC

ATTAAAAATTAAATTTCACACTATTGCAAGTTGTTTTATATTTGCATTTATGGTATTTCCCAATTTCTTGGCACCAAATG

GCATTTTATATTGAAAATGAATTCCAGTGTTCACAAGTGTCTGATCAGGTCTAGTCAAGCACAGTGATACTGTACTGTCC

TGCTTTTGGTAAAGTGGCTGGGGTATGACACTCCATCTAGTGTCAAGGTTAAGAATGTGTGGAGCTAGATCTCAACCCCT

CCAACATCATCCTGGCAAGGAGCTTACACACAGGGATAAATTTTCCCTTTTTTAGAACCTGTTTCTAAAATATTATTTAA

TTTAGAAGCATTCAAATTACCTTTATGGCACATGTGTAAGGTTCACAGATTCAGCCACATATGATTACAACAGAAACCTG

GCAGCAGTCTGCTATGTAAACAGAATGAAGTCACAAAGGATTGCATTTTTTGCAGATTACATTCCATCCCAGTTGCCAGT

GTCATGTTTGACAGTGGCTCCAAGGGCAGATAAACTGGAGAAGGCTGTGGGCAGGAAGCCCCTTCTTGAAGTGAATGTCA

TTGGTGAGTTTGACATTAGTGAAGACATTACAGATAGCTTCAAAATTAAATCATTGTGACTCAGTTTGTAACGCAGTCCT

AGGCTTTGAACGTTTTGTACATCCATCATCTTATTTCTTTATATTCATGAATCAAAATGTTCTGTACATAGGTTTTATTT

CTGTCTTTAGGTGAGATGCAAGAAAGAGATGTCTTCAGTAATCCCAGGTTTTGGTTTTATAGGTGAAGGTGGCGGTGGTT

CAGGTTCGTGGGAAAAGAGTGGATCCAGTCATCCAAATGGCAGTCCTGTTCCCACCAGTACTGCTCAACACAGTAGCCCA

GCAACAGATTCTCGGCACCATCTGATAACCCCACGAGATCAGGTGCATTCAGTGCTGAGATCAAACCCTGGCCGGGAAAA

TGTAGTGTTTGGAGGTAAAAGAAGATTGGGTACATTAACAGACCAGCTTGTAGGTAGTGTATGTCTGCAGTGGGTTCATG

CTAGTTTTCTCCATTAATTAACAGACTGATCTTAGATGTCATTGAGTGTTTCTCTGTGAATGGTGATACATGACAATATG

CATACATAACAGACATAAAGAAAGAAGAGCTTTCCACAGGAATGGCTCCTGGCATAACACAAGAGGGGGAAAGATATTTT

CTTTTTAAAAATTGGATTTTGTCAGCATGCAATGGATAGTGTGATATCCAATGTATCATGAAGGCAAAACTGTGTCCCTG

TGCATGCCACAAGGGCATATAAGGAGAGTAGAGGTACAGCTTCACTCATACATAGTAGATGAAAGTGAGAGGTCAGGTTC

ATGTCTCAGCCACTTGACCCAAGGGAAAGAACCCTAGGACCCACTGATTAGGAGGCTGGGTGGGACCCCAAGATGTTCTG

GAGAAGACGGAAAATAAATCTCTTCCCCGTGCCAGGAATTGAATCCTTTAGCATCCAGCCCATAGGCTACTGGCTATACT

GGCTATGCTCTGCAAGCGCCAGTCGAGTGTATCGTAAAATGAAGAACTGTTATGATGAATGTGAGTATGGAATCAAGATT

ACAATCTTTGTGTTCGTGATAGAAGACTGAGACAGCCAGTTGCTGTGAATTGTCAGTACCTGACTACCAAACTGTCTCCC

AGAAGGCTAAACTCTGAGAGAACTTCATCACACAGACGCGTCAGATGTGGCACTTTCCTCTATAGCACCAAGTCATGTTT

GAAGCAGTTCTTCTGGGATGTGGCGCTGTGATGTTGGGTGAGTAACACCTGAAGTTTCAAAGCATTGCAGTGCCCTGGAA

ACGTCAGGAACTACTTACCCAATATCATGACATCGCATTGCAGAAGACTTGCATTTTCAGCAACACAGCTGTGAGGATCT

CAAATCGCACAACTCACCTTTGACTTGGTTGTGCTGGTCACTTGAAGTACTGGTACTCCTTCCATTTTTATTACACCAAG

GACATAGAGTGACATTGCTTGAGGTTGCCATATGCTACCCAGCTACTACCAATACTCCAGTGAATGCAATGATTAGATGA

CTGGAGAGGAATACAGTGAAGTACACTGAAGGGGAAGTGTTAACAGCTTGAAAGGTGGGGTGGGAGACAGTTCATGGGAT

GTCTTTCCATGGTCATGGTACAGTCACTAGTAATATACTCATATCTACCTTTCCTGTACGTTACGTCAGTTCCTACATCA

TAAATAATCTCAGGAATATTCTGTTCATAATCATCTCTCGATGTCATTATTTTCAGTTAAGATACCAAACGAGAAATGAG

CGAGCAGTGTTGATTTCTTGTGCTCACTTTCTACACTACACCAAAGCGTTTAAATCCACGTCCGACTTGGAACAGGTGAA

GGGATGTAAATCATTTTCACCAATCAGGACCCTTGTCCAGAGACTACCTGAATTTCCATTGTTTATTCGACATGATGTCA

AAGAAAGCCTCGTGACCCCGTCCATAAATAGAAATATTATATCTTAACCATCATGAGCAAACATAGGTAGTTTCTCGAAT

TAAAAAATAATTCAAATAAAATAATGTGCCACTAAAATATTATGACGCGTCAACAAATTCTTCGGTTGAATGAGAGTGAA

AGATGAAATTGCGGCACACGTGAATTACATATTGTCATACACATCAGGAATGAGTGACCCATGCTAACGGATCACAAGTG

GCGCTGATGTTGGCGATGGGAAATGTCCATTTCTGGTGTTGTGGACTTGAGTGGATTGTTGTATATTATTTTATTTATTA

ACACAAAAGATTGTGGGACAGTAGTCTTATATAGAAGCATTGTATTTATGATTTCGAAACTTGTATGTAGTGAAAACCCT

GTACCAGTCAATAAAAAACCCAGAAAGTGAACAACACTCTTGTGATGTGTATAAACAAAGCTTTGTAAATTCGGTTTTCC

CATGATGTGTAACACTAGTAAACGAGATACGATTTCACATACATCAGAAGATAGAGGTGAAGTTCGCCACCTCAAGAGAA

CAGGGAAAGTTACGGGTCAGTTGAAGAAACCCGAGCCTAAGAAAGAGGTGCCAAACAACTTGGACCTACTTTCGATTATA

AATATTCTGGAACATTCCATATGTCACGCGACCAGGGAGTATGTTCGTATATTGTCGTAAATTGCCTTTGTGTTCACCTT

GGGCTACGCTGACTGTCCACTATTAGAGTGAATGCTCGAGTGCTGTTTCAAACCCATGTGGTTGTCACCCCAGATAGTAT

GCAGTTGTCTACCGGTCACATTTCATAATTTGTTGCTCATAATAAGTTTGTTCTTAACTACTTTGTTAACAGCAATCTCC

ACCACCTGATGAGTATTCATAGGTAGCAGCCTGTCCCTCCGAAATCAAACTGCAGATACAACACTCATCTTTATACTTGA

GATGACTTACGATCTGTGAACATCCTGCTGAAGTATGAACCATCATATGCTTCAGTCTCAGTAAATGAAATAGATAAGTA

CTATTGTCTCTATTCTAAGGCAGGGTTCCAGCAATTATTTTCAATAATAGCAGCAATACAAACCTATGCCAGAAATTTCA

ATTTATAAAGCCTGTAGCTAACCTTACACAGTTTAACAACCCAACAACACTTTGTCATCTTCCCTGACATTCCCTCCAGT

GGGTTATCACCTTGCCTTAGTGGAGGGGACTTGGTAGTCTTAATGATGCTGTTGGCTATGCTGGGTAGTGTACGCTTGCA

AGACAGCAAGAAGGTGAAGGATGAAGCAGGGACCTGCAACAAGTTCGCAGGGGATCCATGGATACATTTCTGTAACGGCT

ACTTCAAATTTTACTTATTTTTTCAATGAAAGGAGTAATGTCTTGTTAAAAGTAATCGTAGAAGTTCTTTAATTGGCAGC

GTGTTGATTTTGTACATTCATTAGATTATCTAATTAAGAAAGCGTCAGTTAGTTTAATTAAGCTCAAGTCATGCAATGCA

TTGTTATGACTACATGTGCTAATGATCTGTATTCATAGCTATGTAACATCAGGTCTGAGATTTTACCAGTTTTGGTTACT

TACCATCCAGACACTCTGTAGTTACGTGAGCAAGGATGTAAGGATCTGATGTTTGTTTTTTGAAGCCAAAAGGGGTCTGT

GAGCAAAAAAGTTTGGGAAACACTGACATAATGCATTTCCTTTGTTTATTTGGATTATGAATGTTTTAACATTTAGTATG

CTTATTTTCAGTGATATTAAAATGGATCTTCAAGAAATTGGTTGGGTGGGCATGGACTGGATTGTTCTGGCTCATGACAG

GGTCAGTCGATGAACCTTCAGGTTGCGTAAGAGGCAGGGGATAGTTCGACTAGCTTGTAAAGAAGGGCTCTGCTCCATGG

AGTGTGTTCATTAGTTAGATGGTCAGTTACTTTCAATAAAGTCTAACACAGGATGCTGCAAATAAAGCAGTTTATTTATA

CTTTCAGGCATGTAAAGTGGCACCACTAGCCTCCATTTTGTAGCCAGGCTAAGAATATGTTTCCTGCCCCCTGTACATTT

TCATGGCACAGTACAAACCACATGGGTAGCTTTTCTTTAATTATCTGGTGCAGAAATACGAATGGAAAGTCCACATTATA

TATGTCCATAAATCTCTGTCGCTCTGATTCACTAAAAGTATGGTATCCTTTGCTTCTTTACTTTTGTGTAATGCAATTAA

TAGTAAACACACAAGAAAAATGACTGTACTGTCACTTCAAACCCAAATGAGAAATCAGAGTTTGAAAGAAAACTTCACAC

GTCTCAACCAAATGACAGGTCATGATTTGCAGAATGATAAAATAGATAAGGACTTAATGGACATCCTGTACTTGAACAAA

GTAAAAATAATTATTTTGGCTGCATGTCTGTGATGTGTGAAAGCTAGTACAGGTCTCATACTGCCAGCCTCTGTGCTTGC

AACAAAAGTCTTTGCATCCACATTTGTGCTGTTTCTGTACGCTGATGACAAGAACTTACTCTGCCCACTTGCGAAACAAT

CGTTCAGTAGCTGAGCCTTCTTCAGGATTGCCTTCTTTTGCGCTTCATCAAACCTATTGGTCTGTAGGTCGACACTGCCA

CTTGCCAGAGTGCTTTTCTGGAAGATGTTGCATCTCCTTATGTCGTGCTTCTATCATTTTCAGTTCCAGTTCATGATCAG

CGTCAGGGCTGTCCCCCTTGAAGTGAATGATCCACTTGACAAAATTGATGTCATTACAACTTCACAGCTACTGTTTCGCA

GTTTTGCCCCACTTGAAACAGAAATATACCACATGATTCCATTGTGTTTCTTTGTGATTCATTTCAGAGTGTCCTCTCCC

CTTTGTATCTTGGTACACCAAGTGGCCCCTTCCTATAGAAGTTACTAAATTTGTGTTGCTATTTTCTGCCCTTGATTTGA

CTTCCCGGAGAATAGAAAGCAACCAGTGCAAAACATGAGAGTTCCTTTGTAACATTTTGCCACCTCCTAGTTCATCTTAC

ATCAAAATAATCCTTGTTTTGACATATATGTGTTCCATTTCTTGATGTGAGAGATCAAATTTTTATCTCTTGAAGAACAA

ACTGTGCAGTGAATATGTAGTTATACAGAAATGCAGTTACATATTTTCATAATTGCTAACCAACAAAGGAGGGAGCTCAC

ATGCCTACAAAAGCTGCTTACCAGGAGCACAGTGATTAAACATTGGTCTTAATCATTCTAAAGCAAACCATCATTACTTT

GTACACCCCCCCCCCCCCAAGCAGAAAGATGCTTACAAATGATACAAATACTTTATATTTGAACACAGTAAAACCCATCT

CAAAAGTTAATCACATTTATTTTTAGTTTGCAGCAGATTAATTGCATCGTGCCTGTTTATAAGAAGGGTGATAAAACAGA

CTGTAGTAACTATCGAGGCATATCACTTTTGTCACCTGCATACAAAATTCTATCCGACATTCTTTTGTCAAGGTTAACTC

AATGCGCAGAGGAAATTATTGAGGATCATCAGTGTGGATTTTGATGTAACAAGTCAACTACTGATCATATATTCAGCATT

TGTCAAATACTTGAGAAAAATGGGAATACAATGAGGAGGTGCATCAGCAATTTATAGACTTCAAGAAAGCCTATGATTCA

GTTAGGAGGGAGGTCTTGTATAATATTCTCATATTTGGTATCCCCATGAAACTAGTAAAGTTTTATTAACCTTACTAGTT

TCATGGGGATGGCTTGAATGGCTTGAAACAAGGAGATGCTCTGTCACCATTGCTTTTCAACTTTGCTTTAGAATATGCCA

TCAGGAAGGAGGGTTCAGGCAAACCAGGAGGTCTTGAAATTGAGTGGTACACATCAGCTTCTGGTTTATGCTGATGATGT

TAATATATTGGGCGGAAACATACATAGCATAAAGAGAAACGCAGAAGATTTAGTGATTGCTAGTAAGGAGATTGGTCTAG

AAGTAAATTCTGAGAAAACTAAGTACATGGCCATGTCTCAAAATCAGAATGCAGGACACAATCACAACATAAAGATAGAT

AATAAATCCTTTGAGAGGGTGGAAGAATTCAAATATTAGGGAGCAACCCTAATGACTCGAAACTCCATTCATGAAGAAAT

TAAGAGCAGACTGAAGTCGGGGAATGCTTGCTATCATTCGGTGCAGAATCTTTTGTCTTCCGGGTTGCTATCCAAAAACA

TAAAGATTAGGGTTTACAGAACTATAATTCTACCTGTTGTTTTGTATGGGTGTGAAACTTGGTCTCTCACACTGAGGGAG

GTACAAAGACTGAGGGTGTTCGAGAATAGGGTGCTGAGGAGGATATTTGGGCCTAAGAGGGATGAGGCGACAGGGGAGTG

GAGAAGAGAACATAACAAGGAACTTAATGACCTGTACTCATCACCAAATATTATTCAGGTAATC

>contig_22029

CCTTATAAAACATTAAGAGACTGGTTTTTATAACCGAAGTGGAGAGTGTTTACAGCGCGGTACGCACTGAGTCCTTATAT

AAAACAGATACACTTCGTCGTTAAAGGGTTAAAGTACAGTAGCCTACAGCACAATATTATGCAGTAACTTACAGTACAAT

AAGGTCGCAAAATACTAGATTTTTTTTGTTTTTTCCTCCCCTGTGGCCCTGCGGCTCGTATGCGGGTCATGGCCTCCCCT

TGCGAGGTTTCACGATAACATTCATTTTAGACACACCACTCTGTGTAGGATTCCTCTGGACGAGTGATCAGCCAAACGTA

GAGACCTCTGCGTGACAACACACAACACTTACAAGAGACAGACATCCATACCGCCGGCGGGATTCGAACCCACAATCGCA

GCACGCGAGCGGCCGCAGACCCACGCTTTAGACGGCGTGGCCACTGGGACTCGGCTATTGGAGCTCTATTTGTCAGTGTT

TTGTATACTTTTAATGACTTTCCTTGGAATAATATTAATAGAATCCCTGTGCCTGTAGTTTGTATTTCCGCCTTATATCT

CTTCGATTGTAATTCAGAGAAAATGCGTGTAAGCACCTTTTCCTTGTGCTGTTGTTACCTGTTAGCACCATGTCGTCAGC

AAATTTTCCTGACGTAAAATGACTATTGGCCTTTTACTTGTATGCGATAAAAAGGTATCTGCATATCTGTCTGTGGCAGC

CATAATTTGTCTGGAAATAAAGGTAGGGGCTCGAAACTGTGGCAGCAGTTGTAGGATGGTTATGATATGTGCCGTGCTAC

ATACGCGTGTAATCAAAATCGGTGGAAGGCCACAAAATCTTGTCTGGTTACAGGACTTCTGTCCAATGGAATAATGGACA

CAAATTTAAGTTACACAAACAAAATATATCGAGTTGCGCCGTACCTTCGAGCAGCGCCGCACAACTCACAGTTCGCTGAA

GTTCCTAACAATAAAAGGCACAATTATGAAATACAATAATACGAAATGAATTGGCTTCCGCATTATTTCTGCTACTTCAT

GGCAGAGGCAAGACATTCTTTTGTCTCCAAAGGTTCCAGACCGGCTCCGGGTCCCACTCAACTTCCTGTTCAGTCGGTAT

CGGGGCGCTAATCTTGGGAGTAAAGCGACCGGGGCGTGAAGCTGACCACTCACCTTCTGAAAAGAGATAAGGAAAGAACG

GAGCTATTTCTCCAATCCTCCATACGCTTTTGTCGCATGTGCGTGGACAACGTGACTTTGTACGTGACGATAATGGACAA

GTAAAAGATACTGACGTGCGTAGCTTCTCACTAACTGTCGAAGGAAAAAGCCTCTGTGGGGCTTACATAGACCAGTTTCA

TTTGTTATCAACGTCAGGGCCCTCCTTTTGTGCCGCCCCAATCGTAGTGCACAGCAAGTGCTTGGCGTATCAGTGGTTCT

GACATGTTCGTGTACGTCTATTTCCCCCTCATAAATAGCGGATTACTACGTCTGGTTGACGTAGATTTCATTTGGCGGGA

TATCAGAATGGAGGTGGTTGATTGCCAGAATACTGACGCTTCTAGAGTTTGCTGTTGAACATTTGTGAAGACTAGCTGGT

AACTGGATTGGTTGACGAATTATAGAGGTATTTACCAGATGATGCTAATTTAAAAGTGTGTGTGTGTGTGTGTGTGTGTA

TGTGTGTTTCTTTCAGGTTTGATTGTACAGGTTCAAGCAATATGAATTAATTACGTGTTTGTTCTGTCTGTGACTTTACC

CTATATGGTTTTGTACGCGTGTGTCTTCTTGAAGAGGCATATTGAAATATTCAGCCAAGAAATTTGACTCTGGATATTTG

CGACTGTGTGTTTGTGCTCGCGATTAATTTCATTGTGGGCTACAGTTACACAAGAAGAGTTGACTGCTTATGCAGCGATA

GAGGTGTGCTCATGACAATTTAGCGATAGAGTTTCTGTGAAGGTTTGCAAACAGAATGAATAAAATTAAACAGTTAGTTC

TGTAAGAAATGACCCGAGCTTGTGTCAGTCTCTTAGTTGACAGATAAAGCAATACGCGCGATTCTGTTCGCTTGAAATTG

CTACCCTTATAATGCATTTCGAATGAACTAAATTTCTGCTTGCCATAGCATGTCGAAATTGACGTCTACGCAGATGCCAT

ACTAATCTTGACCATCCGTCGCTATACGCGCTTTCGGAGACCCCCTGAAAAATGTATATTTAGCATTGTCACTAGGTGGC

AGCACCAACAAACCACAACATACAGTACTTTTGAAGTCAGTTTATCCCGAGTCGAAGTAGCCTTCTACGGAGCGCTGACA

GTGCAGCGATTGCTCGTCACACACGTGGTAAATACAATCGTCCCTCTGTCTGGCATAGCAGATGTGCAGTCCAAGCAAAT

GGCTTACATTAACACATGCACTTATTTATTTTGTGTGCGCATTTCTTTCTTAGTTCCTTCTTCTTGCTTGTCAGCCTTTC

CTTTTGTTTATGTAATTTTATTTCTTAGTTCTTTTAATTTCTTTTCCATTTTCAGTACTCATTCACTTCCTGCTTTCATT

TTTTGTTCTCCATTCACTCTGTTTTCTCATGTGTGCACGTACATATGTATTTAATGTTCACTTATTTAGTGTGTACACAT

TTGCCTCTGATCCATTTTACAAATAAAAAGATCTTATTTGATGAATTTTTAATAAAGGGGTTTCAAACACAGCAATTTGT

TACGTCGTTTCCAAAATGAAAAAGAAACTTTATCTTTCGATGACTTATTATACGTTGAACAGCACTGTGTTCTAAACAGC

AATACAAGTTCATAACAAATTATATTCTGATGTTGAATTTTTGCCGTGCACAACCTTAATCAATAGAAAATTAAAACCAA

GCATGTACACTAATTTTATGATTCATAAAAGATTATCTGACTGCCATGGTCAAAGAGTAGCGATATCTTCTCCTGTCATC

GTGGTGGAATTTAGAGTCACCCTTGAATGGTCCTTCGGCCCGCGTGTCTGTGACTTCAGGTATGCAGTAGGCTACTGTTA

CTACTTAAAACACTCTCGTTGCTATCAGCCAGCATTCCAGTAACAGCTCACGGCTTAGACCAGAAGACTATCTTGCGAGT

CTTTGACATCCAGCCAGGTCAGTTGTGCATCATATTGTAGTTCCTTTGTTCTTGTAATTTAGTTTATGTGGATGAACCAA

TTTCACTCACCCCGAAGATGGTTGCAGTACAGTCAACTGAACGCTGGAACAAATCAAGTACACTACACGGTACCTAAAAC

CAAAAGATGACCTTCAGTTGAACAGGACACATTTTTACCAAGTGCATTCAGCAGCGATGGGGTGACTCTATTGCTCTTCG

TTACGCCTTGTCAGTTGACGTAGTAGTATAGGGTTTTGCCTTCTTTTCTCTACAGGCCGTTGCGGCGAAGCCCTTTGGAA

CAGTGGCAGTTCTGTTTTAAGTTTTCCTCGTCCTGACACATTCTGTAAAAAAATTTGTGTCGGAGGACTTAACTACTACA

GCGCGAGGAACTCTGTTCTCTGCCGCGGGTCACGTGGATGTGCGTGACCCGCCGCACTGCAAGACCGTTGGTGTAGCTAC

CAAGGAAGTCGTTTGGTTGTCGGAAAATCTCTGGGCACTGATGTTCCCATTTTTCTCGTTGGCTATGGTCGCGCCCCCCC

CCCCCCCCCGAGCTGTTAAATGGTCCGTGCAGGTAGGGCGGTCGTCCTCAACTCGCTCACTGGACCCGAGCGTCCCTCAA

CAGCAGCCAGAAGTCGACTGAAGGTTCTGCACAGCGTCACACAAACATACCCATGCACGTTGTTAACCGTTGTGCAACAC

TGTTTGTTTGCACAAAAAATGTGGAACGTGTTTGATTTTCTGCCACACACACACACACACACATACATATCAGAATTGTA

GTGTACAAACCCTCCAACCAGGCCACGCAGTGTCAACGTACTTGTACAACAGCCTCTGTTGGCGCAGATTGTTTGATCGT

GTATGGGCCATTTCAGGTGGGCTAGGTCTCACCATGCTATGTTTTGGGGCCTGTCGCATTCGCGTTGCACAAGGACTGCT

GTTCGGTCTGTTGAGAGCATACGCGGTACCTGTCGCTGCACACCGTAGGGCACTGTTTACCATTTTAGTACGTATTGTTG

CGAGTGCCCGTTCTAAGTCACCGAGCAGGATACTGGCTGTAATATTAATTATACTGACGCGAATTAAAGAGTCTTCTTCC

TTTGTTTTACAGCTGGTGCCTTCCGAGAACCACGGCGTATAAGATTCATTATATAAACAACAAATTGCACTCGCAAATGT

GTACAGAATGGAATATTCTGTCACACAAAGCGTGGAATCAACAGGACCTTTGGTCCAGGACAGTAAAAGATGGCCTCAGT

ATTTAGCGGCAAGTCTAGGTAAAGTATGAAATAGCTCGTATAGTATCTAACCTGCTGCACAGTTCCTGTACAGGCATTTG

ACCCAGCATATGCCTATTAAGTACTAGTTACACATCTGACTGTTACGTATACCTACTTCTGTTGAACATAAAATCAAGTG

TTAATTCCACACTTCCTTTATCTTCACAATGCATCCTCTTCCTATATTTTTATGTATGATAATCCGACAGTACTATTTAC

TTCAGATTCCTTCCTCCTCTGAACTGCAGTAGTGTTCCCAGTATTTGACTGTGTTGATTATCTTGACACATAAAAACATT

GCAATTTATTTATTTATTTATTTATTCATTTACCGTTCAATTGATGTTGACTGGTGTAATTTTACAGATGTTGTTATGAA

AAATAATAAGCCCTTAATATGAACGACAAACGAAGCTGTTAAATAGAACTGCTCTGTGTTACTGCGGTTATTTTATTTCC

ATGGAACCTATCGATTTTTCATCGGCCATTACCTGTCTCACTGGATATAATCAAACGCACGGAATTTGGTCGTGAATTTG

TGTATGAGTAGTGATACTACGAAAGTGGGCTTATCTCAGTTGGTATATTTATATCATCTGTGCTGTCATTTAAAACCACG

CTGCTGGTGCTAGAGAACTCAATTTTTTTGTTGCCACATTTGGCTACTCATTGCGTCAATTTCTTTGCGTTGTGCGCGTG

CGTTCCATTTGTTGTTGGTATCGAGTTACACAAACACCAACACGAATATTGACAACAGCTGACATTACAGCCCGCCACTG

GACTAATCCTGAACCTCGAACCTCACCACCTCACGACTTCTCTTCATAAAACACGTTAATGTTACCCTTCCATCTTCGTA

TTCTGAGTGGACCTTTTTCAAGAGATGTGCCCACCATTGTGTTACGTGCAGTCCCTTGCTGTCCTGTATTTGCCACAATC

CCAACTCATCGTCTTCTCAGTTTTCGCTGTTCTCGCGAACCTAGGCGACATGTACAGTATGCAGCAGTCGTGAAGCATTA

GGCTGCGCCAGGTATGAAAGATTTGTGATTAGTGAAAACAGTCGACGGTTCAGTGTATATGTGTGTGTGTGTGTGTGTTT

GTGTAATTTAAGCACTTAACGGCACTTCTTTATTTTACCCAGCCACACTGTTCGCCGTATGTGTCGGCACTGTGGATGGC

TGGACGGCACCAGCGCTACCTTTTCTGCAGCTGGCGCAGGGTTACAACTCTTCGAATAAATCTTTCCACCACGTAATAAC

TAACGATGAAGCCTCTTGGATCGGATCTCTGGCGCCCCTCGGAGGGCTAACTGGCGCCATCCCTGCAGGATATCTGGCAA

ATATCCTAGGCAGAAGACAGTTGTTGTTGTTGCTGACTGTGCCAATGTTGGTGGGTTGGATCATAATCATCATTGCTCAA

GACTCGGTACGTAAATCACTTCAGTTCAGTTTGCAGCCTTATGTAGGGCAGTTGTATACTCTTTATAAACCATATAAACC

CAAGATTTAGAGAGCATTATCTTACGAGATTTATTTTTCATCACTTTTGCAGCTCTGAACATTCCAGTACTTTCTCGGAA

TTTTCTCGTAGATATCTTTGTTTCTGTCAGCTTTTTCTACCTAATTTACAGTTTACAGATTCAGTTTCATTTGCACCTAC

CTTCTTACAACTTGTACCGAAGGTTGCAGATCAAATAATTTTATTACTGTAAACACACTTGTAAAAATGTAACTTTATTT

TTCTGGTAAATTCGGAAATACGCTTGGGAAATATTTCTGTGAAGATATATTGGGAGCACCCTATACCTAATTGAATATTT

TATTATTCCTTTATGGAAGAGACAATTGGACATAACACTTCTGTTTACTTTTCTGTCCCACTTACTTTTGAAGTACTTAG

CATTGATTATGAATTTATTTTTGATGCTAATTTTGTTTGCGTGTGTGTGTGTGTGTTTGTTTGTGTGTGTGTTAAAAAGA

GACCTCTACAAATATATGCTTATGTTCTCTGAAATTAGTGTGAGGTTTGACGGGTATCTGAACGAATGTGTTTGTGGAAC

ACATCACGGGCAATTCAACATTTTTTAACGGATTGCTAACAGCAAGTGCTTTGGTGCATTCCTTTTTCTGAAACTTACTG

GTTCAGAAAGTATTTTTGGTGAACACAGTTTCGAGGTAATTTTCTCCTCTGCCTAGCACTGAGGTAGGTGTAAAATACCT

TTTCCTCTAAATTTTTCTACCACAGCATCTTAGGCTAGTTCCTTTCGAACATATTCTTCACTTCTTTTGCGATCGAACCT

GCAATAATATTTTATTCTCCTCTAATTTTCCTTCTGATTTACGATTTATAAGATAATATTCTGTCACCTACATTTTTACA

ACCTTTCTGTAATGTGCAATGTCTAATACTCCTTTGCCTTTCCTAAAACACGCCGATCGAAAACAAATATATATTTCCTT

TATCATTTCTCCTTCCGACTTACTGCTTATGGATGTTACAAAGGAACATCCAAAGAACTGCCTTGGGATAGGATCCCACG

CAGCTTTTCTGATATTTGCCATTTTGTGGGCAGTAATTCCATCTGTAATTGCGTAAAGTAACGTGATGAACGGCAAATGG

ATTGTTGAAGCTGGAAGCAGGCCCTTGGGCGAATGAGGGTCATCACCAGTTACTGTCTTACGGAATAGGCAAGGTCAAAA

TGCGATTTGCCATTGTGGTATCGTGCCGTAGCGACAAGGGCGACTAGAGGTGCATACCAGATCCTTAAACTTGGCGGTTC

GTTGAGATTATTGATAAGCTTCACGCTTCGACAGCTTTACTCCAGGAGAATAAGCTTTCTGTTATTGAACCCCTGAATCC

ATATGCCGCCTGCATGCTATATTACCTATTGGCTGTGTTTGTGAAGTCAAGCAAGAAATCGTTGTCGTATGGTCGGTATA

TGGATTTGATGAGTGAACTGGCCTTTATATTGTGTCAAAGTTTAATGCGTCGTACATTTAAGAAATTTAGACCCCTGATT

TTAGTGACGGTTAGTGATTTTAGGCTTAATCAAAGTAGCTTTGTCAGGTATTTCGTTTCCGAACCCGGTAAAGTATGCGA

CGTATGATGAAGAGTGATGTAATTATCCTGTTACAAAACAAGGTGTCATTAACTAAGGAAAACACACGTTTGTGTGCAGG

TGTGCGCAATTATTGACAGCAAATCATCAGAAATCATTTGGTGACGTGTGCTTGTTTGTATGATGCTTCCATTGTAACAA

TATGACTACGTTAGCACAGCAGGTCGCGATGTTTGAGAATGATATGCACGCATCAAAAGTTAATTTATTACGAACTTCCG

AACAGAGCACCTTGCAACCCTGACGGCAGTAATTGTTGAAAATCTACTGGCGCTTTTCGAACTTAATTCGTTATGCCGCT

TGGTTGATAGGGTATCAATAAAAAAATCGGAAAATACGCCGAAAAGAGCAAATGTGGCTTACTTAAAGTATTGTTTCGGT

ATTTACCGGGTGGGACTGAGGAATACCATTAAAGTGCTCAGCCACGCGGTCAGACTGAAGGCCAAGAATAGAAGACGGAA

CATCCCAAGTATGAGGGTGGGCGCTAACCGCTAGGAGGTGCGGTTAGGCTGTTTAATTTCGTTAACGGTACACTGCGCTT

GTGTTCTTCTATAAAGAAGCGAATGCGAATAGACAGCCTGTTTTTCTGTCTGTGCTGCTGTTGAAAACTGATGTCTTCAC

CGCATGTTGCTCTGCACATTCTGGGCGCTGCGACTGTGGCCCCCGCGGTCTACACCGCTTGTCTCGTTTGGGGTGCTGTT

TGCCGGGCTACCAGTGTGGCCACCGCATGTAGTAGTTTCTGGTTGTGCTGTTCGTTTTGTCTGTAGGAACCTTCTTTGTA

CACTGCTGATTGCATCTTGGGTGTTGCTTATGGAGCTGTACGGTGACCACCGTGCTGTACATTTTTCGTTTCGTCTTAGG

TGCTGTCTACGGAGCTGGCACGTGGCTGCCCCACTTTACACTTTCTAACATGCTTCTTCTTATTTTTAACAGGTGCCTTT

GCTGTACATTGCTCGGTTCATCTTGGGTGTTGCTTGCGGGGCTGCTACAGTGGCCACACCTCTCTACAACGAGGAGATCG

CCGAGGTTAGAATCCGAGGCGCTTTGGGTGTCAATATGGACATTATGTTTAATGCCGGTATCCTCTACACATACGTCATC

GGCGCCCTGGACTCGTACTTGTGGCTGTCCCTTTCTGCTTGCATCATCCCTGCCATCTTTGCTGTAACTTTCTTTTGGAT

GCCAGAGTCTCCCATGTTTCTGGTTTCGAAAGGCGAAATTGGGAAAGCGGAAACATCTCTGCGTTGGCTTCGTGGCTTGG

ATAAAGAGAAAAACATTGATATTGAACGTGAACTAAATCAGATGAAGGAATTCGTGAATGAATCGCTTTCAAAGAAAACT

ATTCAGGTCACCAAAAACAGTAAGTGCAGAGTTCTTAATGCACGCATTGCAAACGTTGATGTTAGAAGTCCAACAGCTAA

AGCTGTCAGAATTATACTGGGCCTAATGACCTTCCAACAGTTAAGTGGAATAGAAGCTGTGATTTACTACACTGTGGACA

TATTTGACGAAGCTGGTAGTAGTTTATCTTCATCTATATGCACTATTATAGTTGGAATACTGCAGCTTATATCCACTTAT

ATACCGTCTTTAATAGTTGATCGAGCAGGTAGGCGTATTTTACTCATTTTGTCTGAATTTGGAATGGCCGTGAGTTTATT

CGTTTTGTCTCTCCATTTCACTATGCAAACTAATCATGTGGAAGTGCTGTGGGTAGGGTGGATTCCATTGGTAGCTGTGA

ATATGTACATCATCGCCTTTTCCGTCGGTTTTGGACCATTACCTTGGCTGATAATGGCCGAGTTGTTATCAACGGAAGCA

AAGGTGTGGGTGAGTTCTATGGCTGTGTGTTTCAACTGGAGCCTTACGTTCGCGGTCACCAAACTGTTTCCAGTAGTGAA

TCGTGATCTTGGACCGGCGATAACGTATGGTGGCTTCTGCTTTATTTGCGTATTGGGTATTGCGTTTGTAGTGTTTTTTG

TACCAGAAACTCAGGGGAAGTCAAGGGAAGACATCCAGCGTGAGCTGATGGCAATCTGATATAACACCGAATTTTATTGT

GATAAGTAATATTGTTGATTTATCTCTGGTTAGAATCCTTTTTATGATACTCGATAGAAAATGTGGCGTTGTTTTTACAG

TTTTTATATGTTTTTGTGAAATCTTACAATAATCAACATGGATCGGTCCCAGAACCCCACAAAATCCTATTGTATCATGA

AACGCAAATAATTATAAAAATAATTGTATATATAAATATCATGAACACAGTTTGTACAGCATTGCAGTGTTTTCAGGTTC

GCTGTTGCGGTTTCTAAGAATCAGAGATTCAGAGGTAATTTTTCCGTAACGTGTTAATTGGTAACGTATATGCTCGTAAT

TTTTAGTGACAAATTCTTATAAACAGTAGAACTCAAGACACAGCATAAATTTCTATAAGAAATGTTAACTCAAAGTATCA

AACCTTTTGATTTGTTACGAGAGAGAAGCACTAATGCTTGTCTTCTTGCGCCAAAATTTCTTGTCACAAAAATATATGGT

GTGCGACATTAATATAATAACACAGTGTGTGGCCCATTTCTGAATTTTCATGTAATAGAATTGCATTTCCCAAAGACGCT

CTTCTTTCATGAGGTACTCCCACTGCACCACAGCTATACTTATTGTGGTAATCATCACTCAGTTATTTTTTCCAGTTCTC

CAAATAACAGATACGAATATATCAATGTTTGCTCGTTGTATTTCTTTGACTAGTTATGATACTTTATTTCGCCTACCTGT

AATATTCCAGGCAGTAGTACGCGGTTTTTATCTGCAGGGTTTCTTTGTCGTGTTGTGTGTATTCCGACCTAAATCTTCCA

AAAATGTGGAGTCATGCAGTTTTTGTGAGGCCACTCGTTAAGCCCTGAACTTAGCATACAACTTGCAAGTCGAAGGCCCT

TACAACAAGAGCAGTCCATCCTTAGAAAGAGCGAGTGAATGAGTTCTGCACTTGTGTTGCGCGTTCTTTCGCCCCACTTA

CTAACCCTGTACAGTAATCTGAACCATTGCATTCTAGCCGTCCTACTCTTTCAGGATGCAGACTCCTGTAGCAACTTTCT

TTTGCTGCGTGATTCACTTTTTTAAGCCTGACGTTCGTGTAAATAATATTTAAAAACTATTTCTTAATCAAAGCAAACAT

TACGTCTTTATGACAAAGACAAACTTGTCATCGCTGCTCAGTTGCACAGTCCTTGTCTATTCTGAGAAGAGTACAAAACA

TAAACTGTGCGGACAGTATTGCGTGTCGTACTGGTAAAACAGGTGATGCGTACACAGTAGTTAGCATCGTGCTCTGTAGG

GTTAAATGAAACTCAAGGCAACTCACATACTGGAAGAGCCTCTGTGGGGCAGTGCATTGGACTGGCTGTCGAAGACGTAA

CAAACATTTGTTTACACCGTATAAGAAAAATAAGTGTCCGAAATACCAGGTTATCTGGCAGCTGTCCATGTGCCAATTGT

TCTACAGCATTTGTTTACAGACGTCAACCCTGCACACTGCAGTGTTTTGAATAAGCCTTTTACTAGTCCCTTGCTTGCAT

TTCCTAGCATTCGAACTGTGTTATCATTTTCAGGTCCATTTATCTGTATATGAGAGCCTGCCACCTTAGCCAGATGAATC

CTATTTCTTAAGCTGCACTGTAATTGTGTCATGTCATGCAGCCCAGGTCTCTCACATTGTCTATCCCTTCCAGGTCTCTC

CACCAAAGGGCGCATACATCTCTCAGCTTACCCATATTTCGGCTTCTCTCAAACGTCTGGGTTTGGTCACTCCAGCAGTA

TCTGGTAAAGCCCCCAAATTATCAAGTTCCTTTTGCAGATGTATTTATCCTATCAGCAAGTTCCGTGCAAACTTCCGTGC

ATCACCTTTTCTGAATATTTATGTAAGAAAGTCACTAAAGCAAAGATTTTTTTATTCGTCACAAAAAGTGCGATTTTGCG

AAATTTTAAGTCTCTTTCTGCGTTTTTATTGCCAATTAAAATTTGCTGCACCTTCAAGGTGAACTAAGATTAGCAGAGCT

AACTTTAGCAAAGCCGACATCAGCGGGGCAACATATGCGAAGCGCATATGTCACTTGCTTCGTGTGTACGTGGAAAAGTG

ATCGAGGATCTCTTTCAGGAATGTTGGGGTTAAAACACAAATTTGTCTTGGATTTATTTGTAGTTTTATGCTGAATCTAA

ATACACAGGCGGTTTATTCTATTATTATTATTATTATTATTATTAAAATCGGTATCTGTAAGTGGTAAGTGATGTAACGT

ATAATAAATTGTCAGTTTCTATTCTAGTGTTGTTTTGAGTGCTGTGTTGTATTGTATGAAGGTTTCTAATTTTATCAAGT

GCAGGCTGTTTAGTGTTAACCTGGAGGAGACAGCGCCTATGCGCAACAGCCATTCTGGCGCCCGTATCTTGCAAACGTCA

CGCACACAAGACATTATAGTGACAGTACAGCTGCCTTCACGGGCCCTGAAGATAAAAATCCAGTGTTTGCACCGATCACC

CTTAGGATATAGCCTTACGGATCTCTTTGGTGATTTGCCTTATACGATATTCTGTGTTCGGAGAGGAGGATGTTTTACGT

TTAATGGATTAGGCTAAATTGCATACATTAGGTTTAATTTTGTAATTATTTTCAGGGGGGTTACTTTGTGAGCGCTTCTT

CTGGGCGAGTAACAATGCACATGTAATTTTGTTTGCTGTTTTTATTAAGGACAATATGAAGGGCTATTCCTGACATTTGA

CGTGAAATATAAAGTAATACTTCTAAAATAAATGATTACATTTTGAGATTCAGATGAAAAATGGTAAAAAGTATGTGGCT

GTAAAATTTGGGGTACTAAGAACAAATACTAAAGACAAACTCTAATGCGATGAATTAAATTTTTAAGGCCTGTATGAAAT

TTACAAAATTAACAAATTAAAAATATGTAACATCAGAGCTTATTCTTTACAAGGACAAAATATGAAGTAATAGATAAATG

GATCAAATGCTTATAAACATTTATAGTGAAGAGTCACAAAACAACTAAATAAATATTCGTAAGGAGAAATGTTGGAAGAC

CACGAGTCAGACGGTGTGAGCTCGGCATATATTACGATCCGAAATTGTATCGAATGGGAAAGAAACAAACAGCTATTTCT

GTTCTGATCGAAATAAGAATAAGTCCAGTGTACATTTATCAGTAGTCATGCACGGACACGTACTTGCAGTGATGCTAGAG

GGGGGGAAACACTAAATTATAAAACTATTTAAAAACAAGTAACGCCGTGCAGCGCGCCATTCACTCAAGGTAACCGTATA

CTGTGTTTATGTTTTGAACTGCAGTGACCGCATGCCAGTGTAACCGTTACAACTGCCTTGCATAAGGCATTGCCACCGTG

TACAGTACGTTGCAGTCCCGACTTGTCCGCTCTGTTGATAGTGGCCAGTATACGTCGCAGTTTAAGTCGACTGCTCTCCA

GTCTGTCTGCCAGTTACATGTCATTTTGTGCTCCATTTTCGAGGCGTTCCCCAAATTGTGAGGAATTTCCTGGCTAGCTG

AGGATATGTTAATTTCTCAAGAAGGACTTTGCTCCATGGAGCTAGATAGTTTGTTCGTTTGTTTGTTTGTTTCCCCAACA

GTTACACTGCATCAGCTTTCCCTGTTACACGAGTGTATAGCATACTAGCGTGTCAGTGGTGGCTGTATGATGCGCTGCTT

CTGTTCAGAACAGTTGAATTCACGTGAGTGGAATCTGCAGTACGTGTAGGTATGTAGCACTGTGGGGCACATCAGTCTGG

ATACTGACTAAGTTTGCTTATAAGCTAAATATTAAACAAGAGTAGCTGTACTCTGTACCAATGCATGTATTTTCCATTCG

CCTTCTTTACAACCTTGCATACCTTGGCTTTGAAGTACGTTTAGATTACACTGAAAGAAAACAGGATAATGCCGTAAAAT

AAACTAACTCCATAGACTAGCGTTTTCGGTGGAATACGAAGCTTTGTTATCGAGTTTACAAGGGTCGCAATCGGACCCAG

TCCTCAACCAGCTGTGTTAGTTCACACCTTCGCATTGTATTTCTTTTAGACCCACATTATTATCATTCCTTCTATTCTTA

CGTCTCACGAGTTTTTTCGTCCCATCGGGTTTTCCGACTAAAGTTTGTGTGCATTTTTTCTCTTCCTCTTTCCTAATGTA

AACGTCTGTACGATACAGAAGGTGAAATTAACGCTTCTGCATACAAACCTTACAGTCGGATTATGTTAATAGGACTGCTC

TGTGAAGTAGATGATATGCTGGAGTTTGAAATGCTGAGAGAAGGCCTAGTCTGTCGTTTCCAGGCTGTAAACATCTTATC

GTCTGTTCGTAATGACACCTCTTAAGTGACTGTGATTCTGGGGACCAATGATAGTGCAGGAGCGCATTTGTCACAGTCGT

AGAGCGATAACTAAGTTCTATCGTATTAAAGGCCAGAGGCACATCAGTTCCTACAATTGTTGGTTCTTTGTTTCTCACTA

CTCGTTATGTTTGTGAAGTTATTAGCGGGGTTTAATATTATGTAGATTATACTTCAGCCAACAAAAGGTGAGTATGTGTG

TTTATGTCTTCCGTCGTGCCCTTATGCCTTGTAAGGGAAGGGCAGGGCACATTTTTCGGGTTAAATGTTAATGTGATGTA

ATGAACTCCATGCAGCGCAGTTCTTCTTGAGAAGGTAAGGTCTATTCATAGCTTATGGGCCACGAAGTTTCATTACCGCC

GTCAGAAGACTTGACCACTGAGCCCTATGCTGACCGTGTTACTACAGTCCGCGTCCTGACACGTTTATTTAAGTATTATT

ATCAGTATTATTATTATTGTTGTTATTGTTCCAGTCCACGTTTATTGCAAGGAACTCTCGGCTGGCGTTTAGACTTGAAA

GGCTGAGGAAACCCACTACAATTTTTGGTTAGCATATCCGACTTGCTGCCAACACTACAAACTAGGCGTATTCTGAATGC

TGATTGCATTAGACATGCCCTGCCGCAGCTTATGCATGGCGGCGTGCCATCACCTGACTGTCACAGGAGGAGCTTTATAA

TTTATATTTTGAAACGTGTTTGTAAAATCCGTATCGATTACAGGGTTTGAGAAACACAACGCTGTGAGTGTCTATAATCG

ATATAGTGAAATGTTCATAGGGTTAACTGTTTATGGTGTGTGATGTAGATAAGGCATTAGGACGCGAGAGGGAGATGCTA

AATACATACAGAATTTTGGTATGCAAACTTCAAAGCAATAAGTCAGATGGGGGACCTAATCATAGATGGATGGATAATAT

TAAAATTGTTATTGATGTCATGTGGCGTGAATGTGTGGGCTCGATCCATCTGGCTAGGGATAGGGTCTAGGAGACTTTTG

CGAAGACGCTAATGAACCTTTGAATTACGTAAAATCATCCAATTTTTTGGCTCAGCTGTAGTCAGCATCTTTAAGAGGAC

CTTTTGCTCGGCCGCAATTGCCGTATAAACCCAAAGATGTGACGACATTTGTGGGTTCTTCCTACGAGAGGCCTGGCTTC

AGTCCCACCCCAAGTGGACATTCCTTGTAGGAAGGGAGGGGTGGGTTGTATCCACAGGAGCCAAAGCTGTCTTCCGTTCC

TGTCTGTTCTTACGTCATTTTGATGTTTACCACAGCAAAGAACAGCGCCAACGCTCCCTACATTAGTACTTGAGTTGTCA

TACGTCCAACAAAGAATGGATCGATGGGTGGGTGATGGAAGGAAGGACGGATGGTTGGTTGGTTGAGTTGGTCTTGAAAT

ACGAGTATCATTGACTGGTCGGATGGGTAATTGTTTGTTAGAATTACTCAATATTGGTAAATGCCTAAGACTTTTATTTT

CTTACGTTGACGCTAAAGACTTACCTGCTGTCAACTGGTACAACAGTCGACGTACGTGAATCTTGAAAACATGTCGATGG

AACAACTGGAAGTTTCTGAGGCGGAGCCAATAAGAAAGACTGACAGCAGATGGAGGAAGACATGGCCCCAGCACTTGGCT

GCAAGCCTAGGTAAAGTTCCTTACCATAATTATTTATTGCGTTGTTACGGACGTTATGATAGGAAGTCGCTCATTTGTAG

CTTCTACGTGTCATGCATTTGTTTCAGTGATCTAAGAAACAGCACTGAAATCTGAGGGTATTATGGCGCCAGGTTTTTAG

TGCTCAAATCTTTGTAGTGAGACAGCCACGACCATCAATGACTAATTATACTATTCGAAGCTCCATTTATGTCAATAACT

CGTATTCGTACCGATTCCGACTCGAAGTTTGTAATTTTTACCAGAAACAATATAGCATACAAAGTGCAGTTGTGTTTACG

TTCGATTTTCCTGCAGCATTTGAATTTAACTTTTAACAAAACCTAGTTCTTTATGCTATGTTTGTTTATTATGGTTTATT

CTCCTTCATGTTTTGAACTTCAGGGAGATTATTCACTGACATGAACAGTGGGAAAACGGAAGGCTTTTTGATATAAATGT

TACAGAGTAGTTGTAGTTAACTGATAATGTGGGTAGTACTATATTCAGATGCGTTAAAAATCTTAAAATGTGAACTTATC

TTAAAAGTGGTAAAGACTGTAATTAATGTGTTGAAGTTCTGTAATTAGTGCCTTTCGTTTATTACACACACTGCCCCTCT

AGAACAGAGTTTAACGATATGTAATAGAACTTGTTTTCCTCTTAGATTTATTTTGGGATTTCCCCAGTATATTGTATTTA

AAGTTATTCAGTTTTGTTGATAATTTTATCTCTTTTGTAGTTTTTGTGTATTGGTCATAATCACGTATGTGTTGACATCC

CTAGTGTGCAATGTGTTGTGCAGTAACTGTACGTGTAAGTGTCACGTCTGGTGGGCTCCTGTGGGTCTTGGTTTGCTATT

ACATTACGCTGCCGCTGTTTGTGCTCGGACTGTGCTGAATCTGCTGCAGCGAGTGGGCTTTCTGCTGTTGGTCTTGTCGA

GTTCGTGGAACGGTGTTCTTCCCTTCTGCTCGTGACAGTAGTGGATAATGTCCATGACGCTGATCTCGAAGACAGGAAAC

ACTGCAAACATATAATGGGTTAAATATAGCATGCAAAACTAGGAGTAATGTGATGCAGGTGCGCACCCAGAATTTTTCCT

CGGCGTGAGGGGGGGGGGGGAAGCTGACCTTTAGGCTATACCGTATATAATTGATGTTTGGTTGTAAAAATTATATTATA

AACATCATGTCATCTTAAACATAACGCTGTTTGCAACTGCATTTATGTACATGCGAACATAACTACCTGTTCCATGACTC

AGTCACAGTGTCCAGTCTTCTTGTTTTTTTTAATTTTATTAATTTATTTTTCAGAATTCCAGTGTACTGGTCATCAGCCG

ATTTCAGTGGCTGATTTCGGCTGAAACCTAAATCACGTAAAACTTTTGATATCATGGTGTCTACAAAATATGTGTTTTTT

AAATTTCGGTTTTGGGGGCTTTGGGCGGCTGCAGCCCTGCAGCCCCCCTCCCCCGCCGGGTGCGCCCTTGATGTGATGCA

ATAATGTTAACATATCATTTGTCTGACTCGTACAATTCATTGCTTATCGTTACACGACCACTACATATGTACAGCAGTTA

CTACTACGTACTTTGACACCATCGCTGTGAGAGTAATGCCATCTCATGGTGTCGTCGGATACTACGCTTTTGTAAGTAAG

GTACGGGGGTGTCGACCGGGTTTATCTGCCCCAGTGTTGACTGTGATCCATCGAGTCAGATTTTTATACGCCTGTCAGTG

TACCGGTTCAACAAATTTGACGAGCTCCAGTCGCACATATTTTATGACCTTTAAATCTTGTTAACAACTTCCGTTTGCTG

TGTCAGTCTTCTGTATAGACAATCATGTTTCATCCAGTCTTAAGTACATGGATCTCAACTACACATTTATGAAGAGCATA

AAAAACCTGGCTTCAAAATATTCCTACCAAGGTTGCTATAATCTGATCGATGGACTTGGGGAAGTCATTTTACTCCTTAT

TTCGCTTATTAAAATCTTGTTTCTGTCGCCTGAGAGGTGTTTAAATCAGAGGTTATTAGGTCATTTGGCGAATTAAGCAA

TTTAGTTAGACGGATGTCCCACCAAACGTTGTAAAGTTGTAATTTTGTATTATGACCTAGTGTTTTCGTTATCCTGTACG

CCTTCAGCTTCCCTGTCGGTTGTTAATTTTCTGAGTGTGATTGATCCTAGCCGCGTTGTCTGCTGTGTGTGCCGGGGCAG

TAGACGGATGGACAGCAGCCGGTATCCCCTACCTGGAGCAGCCCTACAACCTAACTGACAACGCCACCGTCCCTGGCATC

ACATATGATGAGGGGTCCTGGATAGGGTCCCTGTCTCCACTGGGTTCGCTTGTGGGAGCCATCCCAGCCGGATACCTAGC

CAGTCTGCTGGGCAGGCGACTCCTGATGCTCACTATGGCCGCGCCGATGTTTTTGGGCTGGGTTATGATAGTGTTTGCTC

ACAATTCTGTAAGTGCAGTAAACTAGATATACCTAGCATTCACGATTTCCCTCCACTTTGTATTTCCATGAACTTCAAAA

GTGTACGTACCTTGTTCTGTTCTTTACATAAAGGGCACTTTTCTCTTTCTGTTCTTACCCTCAGGGCCCTTGATTTCCAA

ATTACTAACCTAAACCATGCGGTTTCACTCCTGTCATTGTTGTCATTCCTATATATATACATGTATATACTATATATGAT

ACGTAAATCTGTCCCCTTCTCCTTCTTCGAATATCAATATGCAACTAAAAGCCTCTTCCCCATTTTCTCTATACTCGTCT

GTCTTTGCGTATCAGAACATCTAGTTTTATTAATTTGATCTGCTGCCCTTCCACTGCCTCCTGTCTGTCTTGCGAAATAT

ATCCCACCCCAATTTCATGTAATTCCTCCCTCAAATTTGCTGCCCAACCTCCCATTGTCAAATTTCCTACCTGCACCTCA

TAACAATTTTCAAAATTCTTCATTTTCCTGCAATAAAATGGTACTAGCCTCCATAACAGTCTGCCGCTCCTACTTTCTGC

ACCAAGTTCACAATCTGCTGTTCCATTTACTGCACTGCTGGAGATTCTTAGGACTGCCTTAAAAAATTTCCCCTGAATTC

CATCATAGGGCTATGTTGTTTCTTTATTATGTGTACAGAGGTAAGCAAATTCTGTGGACCTATACGTGACTCTTTAAATG

TTCAAACACAATAATTTGGACCTTGCTGGGGTGTCACCTCCACGTTTGAACAATCAGTCTCCTCTGTACATTATCCTGAC

TCATGACTATTTTTCTGTCACAGATTTTGGTGCTATACGCAGCTCGGTTTATTCTGGGTTTCACTTGTGGTATTATCACA

GTGGCTTCACCCCTGTACAGCGAAGAAATAGCTGAAGTCAGGATCCGAGGTGCTGTGGGCGTTTATCTGGACCTTATGTT

CAATGTGGGGATTCTTTATGTCTACGTTGTTGGTGCCATACTCCCATACATGTGGATGTCAATTGCATGCACCATACTGC

CGGTTTTGTTCGCTGTCACCTTCTTCTGGATACCTGAGTCACCCATCTATCTTCTTTCCAAAGGCCAGACTGATAAAGCT

GAGAAATCTCTGTGTTGGCTTCGAGGTGTCAGTGTTGGACACAGTGCTGAGATTGAAGATGAGCTGAAACAAATGCAGAG

CTTCATAAAGGGGTGCAAAGTGAGTACATCTGTCTCACGTGACCAAACATCTCTACCTTCCAAGGTTATTAATTTCTTCC

GGAGCATTTATGTGACATCTGCAACAATGAAAGCCATGAACATCATATTTGGTCTGATGGCTTTTCGGCAGTTGTGTGGA

ATGAATGCTGTACTGGCTTACACTGTTGAAATTTTTCAAGCAGCTGGCAGTTCCTTGGACCCTCACTTATGCACAGTTAT

TGTTGGTGTAATTCAACTTGTGTCAACTTGCATCCCATCATTTATAGTTGATTGTGCAGGAAGAAGAATATTGCTTATAA

TTTCTGGTGCAGGAATGGGGGTGTGTTTGCTGGCAATGGTAATCCGTTTCTTTCTGCTTGATCAAGGGATAGAAGTAAAA

TACATAGGGTGGCTTCCTTTGATTGCTGTAAATTTATATATTGTAGCTTGCTCAGTGGGGTTTGGTCCTTTACCTTGGTT

CATGATGCCAGAGCTTCTAAGCAATGAAGCTAAAAGTTGGGTCAGTTCAATTGCTGTTTGCCTTAATTGGGCATTGGCAT

TTCTTGTAACAAAATTCTTCCCCATTATGATGAATGACATGGGTGCAGAAGCTACTTATGGGACATTCTTTGTCATATGT

TTGGTTGGTACCGTATTTATTGTGGTTTTTGTACCAGAAACGAAAGGGAAAACCCGAGAAGAGATACATAGGCAGTTATC

AAGGAAATGACTGTAGTTGTTATTCTACTGTTTGTTCACTGGCCTGGATATGTATACAAGAAATTCTGAAAAGGACTTAG

AGTGTTTATTTTTCTATGATGCTTCCGTCTACTAAGCAAGATGAACATAAATTGTATATTAATAATTAGCCAACACATTG

GTCAATTTCTGTGCACAGTGTTTACTAGTACAAACTCCCACAACCTAACAGTTGCATGACATGACCTAAACGTTTTGTTT

TGGCACCATTTTTTTAATTTGTATTTTATGAAAAACTGCTCATCTAAAACACTAATGATTTTTGTGCTCTCTCACCATAC

CAAACATGAATCAGTATGAAATTCCATATTACCAGTGCACTTGTGTGCCTGTTATTGCTATCAAACATAATGCTAAATGC

AGGTATCGTACTGGCATGTTGTTTTATGTTATACAGAGAAATGAACCCTAATAGCAGTTACGTATTTTGTAAAAGGTCTT

TTGTCGTGCAAAATATCAAACATCTGCATTAAGTAGCGCTGCTATCAGTCCACTTAAGTAATTCTCATCTGCGATATTGA

CATAATTGGCAGTAGGAAATTAGGAGCAGTAGAAAGAACTGTGCACTGATGGCATCATACCTGCCCTAATGTTCGTGTGA

GTCTCTTGGGTAGTCAAAAGTTTTAGACACACACACACACACACAAGTGGACTGTGTAATGTTTTAGGTGTCGTATAATT

GTAACTTTTCTCATAAATTAAAATAGAAGAAAATTTGTTGTCACAGGTTGGAACACAAATTGAGTAAACTGAAGCAGAAT

TTTAGATTTATAAAAATACTTCCATCCCCTGTTGTCAAGACTTAAACACACTTTAAAAATAACACTTGGGGTCAATTCCT

TCAAATTTGCCAGGAACTTACACATAATTTTATGATGCAATTGGTGCAAAAATGGCACAGCCTGAATCTTTTATTGTACG

TATTGTAATTGGCTTGTTACATGGTCTGTAGTGTTCATTGAAAAGTGATAATAACTGCCTGTGTTGATGGAGCATGAAAC

TTTATCATCATGTCATAAAAGCCCACCATTGTTCCTTGTCCTGAATCAGTTCCACCCAGTCTTCATGAGTTTTTTAAAAT

CCAATTCACATCTTTTATCTACATCTCACCCATCAATTTCCCTTGAGATTTTTTTTATTTAGCATGTTATATTTACTTCT

CATTTTGCTGTGGATTTTTCTTGTTCCATCCATCTCATACTATATTTAACTGCCTAATTACTGTCAGGCTAAATGTATAT

ATTACAAAGATTGTCACATAATACTCCTGCAGTCCTTCCTTAGTTCATCTCTCTTAGGTCCTACTGGCACTTTTTTCTCA

AACACCCTCAAAATGCTTTCCCTTAGGGTGAAATCCCAAGTTTAGTACACATAAAAATAGCTGGGTAAAAATTATAGTTT

TTATATTTCAATCCCTAGGTTCTTAAATAGGAGGTGGAAGGATAAAAGATTCCTAACTGAAATAAATCAAACATTCCCTG

AATTAATCTGCTCTTAATATAAATATATACGTGATAGTATTACAGTGTTGCTCCCAAATACCTCATACATTTAGCTAATG

AGAGATTGTATTATAAGTGTACAGCAGTAGCCATTTGAAATTTATAAAGTGTGAACAGAAAACAGCGAACGCTCTTCTTC

TCACCTCAGGATAGTAGGTTTTGTTGGGAAAGATCATATCATTCAAGGCATCACAAACAATTATCTTCTTTTATTACCAC

TTTTATTACAAAACTGCAGGGGTCTGTCTGACCGTCTCAATGAACAGGTGCTACCCCACTGTTTTTCACCTGATGATGTA

AACTGAACTGGAATCCGAAATATTGTCTTCAATCTTTGTATTTTTAAATGTTGGACATCGGACAAAGTGCAACACACTGA

GTTTAGTAAATGTGATATACCACTGTCAGAACACTATAGAAATGAACAGTTTCCTTATTTGCATTTTGCATGTAGGAATA

AAATAAGCCTGTCCATTGTAATCTGCCTAATAAGGATTGCAGATGACTCTTGGCAATTTTGCAGCTTGATAAGTACTGAG

TTATTTTTATTTACAAATGAGCTTTTTTATTGCACTCTTTGTAATTCATTCTGAAGAAAATAGTATTTCAGTGCATGATT

CAACTAAATCCTTAATTTTTATGTTCTTCATCATGCATATTGTACAGGCATCAGTGGGACAGCAGACACAAACAATACAC

ATAGCGTTAATTAAAGATTGTTCTGCCTTGATATTCTCTGAATTGTCTTCTCTCTGTCAAAGCCATTACATACATACTGA

AGTTGCCTCGAAGGTATTGCCAAATGTTCTCAAAGTTAATAAATTCAAATCTTCTACTCCAATGACAAATATATTACCAG

TAATAAACTATTGTAACTGTTCACATCAGTTCCATGACAAATATATATGAGTGACGTAGAAACCCAACCTTTTGGGTTAA

CACTGATTATTTGCTGAACGCAAAAAGGTCATCCAGGCTCGGAACTGAATGTCGAAACATTAGCCTACTGATCATGTCCA

CTAAAATGAATTTCTCAGTTACTCTCATTCAGATGACCATTAGGAGCACCTGTGCCACAGACTTCATAAACCTAATCGCC

TTTGCCAGGTATATATCAGGCCTGTGTGTGAAAAATCTGATAATTTGTGCCTAGTGCCACCACTGCCTTCACTGCAGTCT

TCTTGGGCTATAAGCTGCATTGAATTTTTAAAAGAACTCAGAGACAATGAAGGTCTTCCTTACCAGGGTTCTGATATACA

TATTACAATTTACTGGGCCCAGTCACCACGAAAGATTCAGATCATATGTATATGATTCCTTACTTGTTTGAAAATAATTG

GCCTATTGCACAGGCTATAAAAGTAGAACTGAATTAGGAAAAATAAAATGGTTATGAATGAAGAATTGAGGATGTGGATG

AAAGAGGTCATGACCTATTTTAATGCATTATTTAGCACTTACCTGGAAAGACTGAAGAAAATCGGAAGTAAAGCTAAATC

AGGTTAGCTGATGTCTGACCTGAATATCTGGCCTGGAACTGCCTGAATGCATGTAGATATGCTAAGCAGTATATTATGAA

GTTCAGTAGTAAGTTAGTGAAAATGTTCTTTTTTTGTGTTAATTTTAAAGAAATTACAACTAGAGGCAGTAACTGATGTT

TATTAGCATACAATTACAGATTTAAATACTACAGCGACAGTTCGTTTATACAGTATTCATTTTATAGTTTAAGTATTCCA

GTATTAGTTGTAATTTCTCTTTGACTGTGATTTATCTCATGTATATGCTTGTTCTTTCATGCTGAATTATGATACATGTA

GACTTTATTTGAAATTTGTATCAGCTAATTAATTTTACTGCTGTTTGATAGTTGCAATGAATGAAATGCACTATATACTA

CATTAGAGATACATAACACTTGCATTACAAAATATATTTTCCAGTATTCTTATGAGACTGTAAAATGGAATTTCATGTAC

TTTATGAAGGTACACAGAAAAGGCAGTTTTAAGGCTCAACTGAGATGCACAAGAGGAACAGAGTTTATTCATATTAAATA

GTGACGTGTATCAACATAGAAAAATTTACTGGGTTGCAGACTGGCATGCTGCATGGGAACTTGTTTCCTGTATCCAAAGC

AACAGCCTTTGGTAGTTACTTACAAGAATTTTATAAGTAAGGAGAGCACAGTGATGTGTGAAATATGGATATTTATCCGA

AGCAAATGTAAAATGGATTCACCAATCCATCCTGTAATGTGCATCTTTGATTATAACACAGTGGTCGTTGTAATGATGTT

ATTTACCAGCAAAATACTTACTTCATAATCATCTTCCAAAAGCTGCAATAACATGAAAGGAAATGCAAGGGTGTGTCATG

TTCTAATTGTGTGGTGATTACTGAGTAGTTAAATCTGTGTGTATTAAAGTTGTTAAATATCAGCCTAGTGTTTGAATAAA

AGACTGACTGTGAGATCTCAGGAACATATTTGCAATTATCCTTTAATTGCAAATGTAGTGGTATATTTAATATTGACTGG

AAAATATAAACCTTATGGAAGAATTCCATATTTTGTTTAAGAATGTTATACAGAACCCCATATTTATTTAATCATTGATG

GCATATTTATTGCTATCTTGTGTTGTAATATTGGATGTTTCTAACTTGAAAAAATTAATATAAATGTGTGCTTAAACTAC

AATGGGATGATGTAAAGTTAGAACCAACATGAGATATAAATGGAGGTGGTAAACTGTACTTGAATTCGTACTTCATTTAT

CTGACAGACAGTTCTTATGTTTCATGTAACTCCTTCACAGTTCAGTATTTATAACTTTTACTGTATGTCGTAGAGTGTGA

ATGTTTCAGTGTCTCTAGTCTGTGTGCCATCCCAAAGAAGGAGCAATGGTGTGAGTATGTAGGCTAATCCCATAACCAGT

TGAAGCAAGCCCAGATAACTGAGTTTCCATACCAGTAGTTCTCAACCTGTTGGTGCTTGCAGCACAATTGAAATAATCAT

TCTTTTCTGACTCACTGAAATTAGAATAGAGAACTAACATTTCTAGCAAGGGTACCTGTGTTTTTAATAATGTTATTTAT

TGTATAAAAACCTAGAATTATGATCATATAGTGTTGTTTAGTGTGACGTACACAAAATTGTCTTGAAACATATAGATGAC

AGCCTTGAAAGGAATATCAATTGAAGAAGCTTAAGTAATTGCAGTATTTCCTCATTCATCACAAAATCACTTTTGTTTGT

GTTGTTCATATGTTTTCTTCCTGGTGAGGTCAGAGTTCCCATGGGTCCAGTCCAGGGGGGCTCTAGACAGGTTTAAACAG

GCTATCATAATTGCTAGCTTCATCATTATTACTGATTAAAATTTTTGTGCCAACTAATAGAAATCCAGTGATCTATTACT

GGAGAATATGTGATGATCCAGTACTGGAGAATATGTCACCTACTTTGACTGTAGAGGCTGCTAGACTTTTGGTAGCAATT

AATTATGGACAGCCTACGGGCAGGGGTTGCTGTTTCAAACAGAATTTTTTTCTGTCTATCCTTAAATGTCGCTCAACGCC

CTGTGTCTTTCTCTGGAAAATACTTCCCCTCCCATTTAAATTAACATGGAAATTTTACTCAGTGTGGTTCACTATAGGAC

AGGCATCAGTGCTCATGTTTTCTGCAGCAAATGTCACTGCTGTTGTATTTAATGTTCAATGAAATGTTAGTCAGAGGGAA

ATGTGTTGTACCCAAAGTTTGACAACATGATGATTTTGAAAATCTTGGAAGTTTGGCAGGAGACAGCATTGTAAATCATG

GTCTGTGGTCTGCATCAAATGTGGAAACAGCTAGCTGGCTTGCTACATCTATCATAATAGCACCAAGCCTAAGCATATTG

TACAATTCTGCACCTGTGTAAAAATGTTACTAATTAATTTCTGAAACATTTCAGCATTTTTTATATTGTACACTGAAGTT

ACTGTGTAACTATTGTCTGATCTTTGGAATGTAATCTAATATACTCAGTTAATATTTTGAAAACAGGTTACACTATGGGC

TTCCACTATATGGTTCAGTAATGAGAGGCAGTTTCTTTTCTGATATTATTGTTTGGCGTGAGCCAGCTGGTGTCATCTAA

TCATCATGTAGGTATATATTTTGGCTTTATAAAGCTCAATATGTTGGCCAGTGTTTTTACTGGCAGCATTACTTTTTTTT

CCATAAAACTATTTAGGTTAATTAAAATGTAAGTGAAACCAACAGTGAAGTTTTGATAGGGAAACATTTTTATGATACAT

TTTATGCTCACAGTAGTCTCAAAAATGAGATGCTTTCTCATTGTTACTTTCAAACTTTGCTCTAGAATATACCATTAGAA

AGGCCCAACAAAACAAGGAGTGGTCAAAATTAAGTGGGGTTTGTCAGCTTCTTTGCTATGCAAATTGTGTTAATTTATTG

GGTGAAAATGTAAATACTGTAAAGAAAGAAACAGAATCGTTGTTAGTCACTGGAATGGAGGTTGATCTAGAAATAAATGC

AGAGGAAATCAAGAATGTGATTTTGTCTTGTAACTGGGTTGCAGGACAGTCCATTCTTTGAAAGTGTGGCAAAGTTTTAA

TTTTTGGTATTGACACTAATCAAAATTATGTTCATGAAAAAAAAAAACAGTTTGCCTATTTCCCTACCATGAAGGCATAG

AAAAGGCTTAGATATATATTTCCAGCCATTCTTAACCTTGGGACTGGATGAAGGCCAGTCATCAGCTTCATGTCCTGGCT

GCTGTACCCTCAGGGAGAGAACCACCAGCACCCATTCAATAGGAGGATAGGCTTCAGAGCTGCTCTGCTCATTTTGGAGA

ACAGGAAAATCTTTTATCTCTGCCACGAATAGAACCTTGGATCATTCTGCCAGTAGCCTAGTCCTTATACGGACTGTGCA

ACCATTTCATGAGAAAATTAACAGCAAATAAATTCCTGGGAATGCTTGCTGCTGTTCAGTTTAGAGTCTTTTGTCCTCTC

ATTTTTTACTTAAAAAATTTAAAGACTTGAAATGTAGTAAAATATATTTGCCTGTTTTTCTGTACAGGTGTGAATCTTGG

TCTGTCACATTTAGAGAGAAACAGTGACTGAGGAAGTAAAGGAGGCTAGATTGAACTGCAGAATGAGGCGCTCTATGATT

TGTACTTATTGCCAAATATATAAGGATGATCAAATTAACATAGATGAGATGGGTGTTGCATGTGGCATGCATGGGAGAGG

AAAGAAATCCGTAAATAGTTTTGATAGGAATGTGTGAAGAAAAGGACTGCATGAAGACCTGGAAATAGATGGAAGGATCT

TTTGACTAAGTAAAAAACTGTTAGGTTCTCAAGAGGGACTCTGATCAATGGAGTTGGGTAGTTTCATTTGGCATAGGGTG

TTTCTTTCACAAAGAACAATCTGTATGTAGTGTGAATGTGGAGTTCCTGAGATCATGGTAGTATTGTGGGATTTGTTTAG

CGTTGTAATTGCAGTCTGGTTTCAAGAATTAATATTTTATAGTTTAAGCTGCTGTTAGTTTTTATTTGTTGCTTGGATAC

ATTTTGTATGTTTTATAAATGCTTGTTATGCAAGGCACTGTCATTTCTTGTGTGAAGGTAGAAAATACCTGAGTAAATAT

CGGAACTTTTTAGTGCTCTTGTATGCCACATATACGAGTACATTAAAGTTACTGCAAAACATTGTTTTATACAGGTTTGA

AGTCTAAGCATTTAACAATAAGGTTAAATACACATACACATATATATGACCTCTCATTTAAGTTTTCCTTACTTGACCAG

AACAACCACTCACAGGTTGTTAAACTGTTCTGCCTATATAGTAGTTTCGAATCATCCAGTACTATTCAGTAAGCAAATTT

TGAATTGAAAAGGATGGAAACATGTTTGTCCTTTTGACAATTATTTGGTACTGCAGAGGAACGACATTTTGCCCTCAGAT

GGTTGTGTCAGTAGTGAGACTGTTTAATCCATTTAAAATGTACCATAAAGCACAAAAAGGTCTTGTGGAATACTGTCGTT

TCAAATTACTGCTATGTGTTGGATATGGTGCAGCGGTGTGGTGGGGAAAAAAGACGGAATTCTGTTGGATAGATAGGAGT

TAACCTTTTTACCGACCGGGCCTCGCTGCTACCATTACTCCGTTATGTTTTCAAGATCTCTGCGACAGGTGTCAGCACAC

TCTGCAAATTACTTTCATGCTTGGTAAGATGCGTGTAGAATAGAGGACCTTCAATGATGGGAATGCAAAGAAACCTGCTG

AGGATATGAAATGTCGACGTAAAATACAGTGACATGGAATGGACATGGACTGGAATCCATGTTTGTAGGTTAGAATAAGT

TACGCCATTGCTGAGCGGTATTCATGCGTTTTGCTTAGTGTTTATGTGTTTGTATGAAGTTTAGAATGAGCAGCGAATAT

TCCCGTGTTTGTAATGAGGCCGTAGTGTTTTGTGTAAGGTAGGAAATAGCTTTATAAATGTTATTTGGGCGAATTTCAGG

CTGCAAAGTGTTAAGTACTATCCCGTCGTTTGCCTGGTAAGAGTGGGGAAAATGACACAAACCCGCCATGATAGTCCGTT

TGTTTGTAAAGTTTGCGCCGTGTAGCTCCGGAATACACGTCAGAAGCGTAATTTACGGTCCGTGCAGATTTGTTTCCATA

ACAAATCCGCACATCTGTGTCTGTTAGAATGTGTCTGATTAACTGTTAATTTTCTTGGCATAAGTTGGGTATCCCTACGC

AGTTCGTACGTAGGTTTGTTGGTTGTTAGATAAGTGTATTGTGTGGATTTGGAATCGTTCACTATTTGGACTCGTATCTA

TCGTTCATTGTTTAAAACAAATTCAAACACAACGTTTCTGAGACAGGAACGGTCCTTGTCTTCAGGCAGAACGTAAGGGA

AGACGTATTCTGGTTTTTAGGTTAGGTAGATGTTTGCATACATTAATGGCATGGGAGAGCTGATAATGCATGACAATTTC

TACTTGGAAAACCAAAAGGGAAATGGGTCACTTGGTGTATATATGTATATATGTGGGAATGATATTAAAATGGAGAGTCA

AGAATGAGGCATGACGATGTGTCAGGACATTCTCGAGTAAGGGTGGCTGTGAATGCAGCACTGAACATTCCAACTCTAAA

TAAAGATGCACAGAATATATTAACCAGATAAGCGACCATCAGTTTTTATACAGGACCCTAATCCCGGAGTTGGATAATTT

CTTACGTAGTGTTTGTCCGACGATCGTGTGAACAGGCTGTGTGTGTTAAAATCCAGAAATGGGAACTGATTCAATAGAAC

TGGTGTAAATCAACATGAAGAAATCGTCGGCTGTGTTGTCTTCCACGGTGAGTTTGTTTTCAATGTAGTTTTGTATATAA

TGCATGGCTGTGGCTACACTGGAGACATTCCATTTTGTAGATTCATTTGTATTGAAGTTTATTTATTGTCGGTTGTTCTC

ACAGGGAGTTTGGCCGGTTAAATCCACCATAAAGAATTACACACGCATTCTGTATGCTTGTGTATATTGTAAGTTACTCC

ATCACCAAACTATAGTTGTATACAGCAATTTATGTGCATTTTAAAACGTGCCATTTCGTGCTAACATAGTGGTATACTAG

GTTTCATTAATTCGAATATACTTAACACAAATTCGAATTTACAGTATTTGTGGTCTCAAATGCACAAATCCTGGTCTGGC

CACCTAAATTCAGTAAAAACCAACAAATATACAAGCAGAATACAGTATAAGTGGAATATTTCCATTTAAAAGTATGTTCA

GCGAGATGGTTCCGTAAGTTCTCCGAACCACGCTCGAATTTCGTACCACACTGTTCCTCGTTACAGACTTCCTGTGGCTG

TTGCAAAACCCATACGCTTGTTGTCCGAGTCGAATATCGGCAAGATGTGATCGATGAAGAAATCGCCTATCTCGAAAACA

GTTGCATGTGTAGACGGAGAGAAGCCGGAGTAGCACACGTCACCCTGAAACACAATAGACGTGATGAGCATAACCCTGAA

ATTAGACTGTGTGTTCGTTTGCTTATTCGTTTATTGCAAAAGTGAGATACCCCATTTTGCAGATGTAGTGTGGAGAACGA

AAGTCTCACTTAATCGACTCGGCGCATGCCAACGCAGATACGTTAGAAAGAAAGAAAAAACAGTCCCGTTCTCAGTTTGG

CAAAATAATTGCACAGTAGCTACGCAATTTTTTTTCTACGTAATCGCCATGTTTTTCAATGTGTTAAGTCCAGTCATCCA

TAGTACCTGATTCCATCACAGTGCAGCGGTTTCGGTTGGGAACCAAGTCTTTTCGTATTGATACTTAGCATTCATTCTCA

AACTCAGCACTAGCTAAGTCGGTATAATGACTAGGCTGCGGCCTGCGGTCTTAACGACCAAGTAATTGAGGTTGGATTCG

CAGCAGGGGAAGAGGTTTTTCGCTTCTTCACGGCATCCAGATTGGTTCTGGGCCCACCCAGCTTCCTTTCGAAAGAGAAT

CCGGGTCTCTTTCCCGGGAATAAAGCATCTGGGGGGTTTGTAGAAAATTTGTTAGTCTAGTAATTGTTCTGATATCCTTC

GAAAACGTCTATGCTTGCATCCCTGTTTATATATCGCTACTTCAGAAAGGTACAAATATTGTTAAAACACGGAAGAAAAC

AACCCAGCGTGCCGGCTGTCTTATGGCGCATGTAAGAAACAAGTCGAGACTATTGAAAGACAACAGAGTGACAAAAGCAG

CAGCATGAGTTGCCAAGGAAACTGTATGGCAAGAAACTGATGTTTTCAAGACGATGCGAATTAAATTCTGGATATGGCAA

GCTGCACATCTTAGTCGGCGTGCAGCAAGAATTTTGCTGTTGTTTATCTGGGAGGGAAACTGTGTTCAGGGTTTGATACA

GTGGATTGCATGTAAGAAGTTGTGCTGGCGTTGTCAAGAAACACGTGAACGTCGTCCGTAAGTTGTTTCTTCGCACTTCT

ATCTTTAAACTGAGTTGCTCATACAGTATGATTTTTTTTTTTTTTACATTTAACATATTTTTTCCCGTGTCTTATTTATC

TAATTTTAACCAAATTGTGTGGTTTTGAAATTGCACATACGATTTCAGAGAGCGGTTGTGCAGATGATACCTTTCCGATA

AACTCAGAAAACTGATTAATAACCCACTGAAGTTAATTTGTAAACAAGTTAAATAGTGCAAGTTACAACTGAATGTACAC

AACATATGGTGATTTTACGCGTGCAGAAATGACGATACCTCTCCCGTTAAAAATCGTCACAGATACGATTTTTGCAACTC

TTCGCTGTATGCCCTGTAGGGTCTACACTGTTGTAGTAGATAAGAGATCGCGCCTCTAAAATTCTTATAAATTATTTTGA

CATGATAAATTGTAATTGGTCTTCTAATTACATTGTAATAGGTCGTTTGTGTATTATAGTACAGATCAAGTAATCCAGGT

ATTTGACATAAACGCTACTCACATTCCTTTGGACGTGGTATTCTGCACTGATGTTAAAATTTCTCCCTCCAATCACAAAG

GTCACGGCTGGCAGGTTAGCAGTCTCGTCGCAGTTAAACTGAAAGGCAAAGAGAACCCCATTAGACTGACAGCGCGGTGA

TGCCATTCGGAACGTCAGCGCAACGTTCGCGCTTCAAGCCCATGATGAAACCAGTCGTCTTTGGAGGGTGTATTTTACTG

TTTTGAAGGTTTACGTCTTGTGCAAGTGTCAGAAGCGTGTTGCGAAAGGCATTTTTTTTTCTGCGAGTAAACGAACTGTA

TTGTTCTCTCACTCCATACGGCTTGTGACATAATATTTTCGTTTAACTCAATTAGTTTTAAGTCTCATGTTGGTTTGTGG

ACGTGAATTTAGATATGCTATGGTACCGGAGAGGAAATTCTTAAGAACAGCTGTAGGTGATATGCATAATATACATGCGT

GTCTGTGTCTTGCTAAGTGATGTCGAGGCAATGGCTGATGCATACCACAACGTTGCGCGCTATCGCGCAGTCAGGTGACG

CTTGTCCACTCAGTTACGGCACATTGCATATGCCTTATCAAGCAGTCGTGAGTGGAGAAAACGCCCAGTTGTAGCCGCGT

CAGCAGTTGCATTTGCTCTTGCACGTGTTAGTGCGGGAGTCCTTTTGGTGTAGGACTATGTTGACGTGCTTGCGAGTGCA

AAACACACACACACACACAGGACTGTGCAGTGTAGACAAATCACATAATGCAACAAAGGAGGCTTATTAACCTTGTAAAG

CCTAGCGGTAAATTTACGTACCACCAGGTTTAATATTAAAAAATTCTACGTGGTGCCCACATTGCGTTTATGTGTTTTGT

ACGGATCTCAGAACAAACAGCAACTTTTGCCTTATAAAACATTAAGAGACTGGTTTTTATAACCGAAGTGGAGAGTGTTT

ACAGCGCGGTACGCACTGAGTCCTTATATAAAACAGATACACTCCGTCTTTAAAGGGTTAAGTGCAAAGTGGTTAAACAA

ATTATGGCTGTACTGTTCCTAAAGTTAGAAGTAGGGTTTAACCGAGGTCGCTTCATCAGGAACTTTGTTTGTTGTTCAAC

TATATAAAGCCCTTTAATCGGGGAATTCTTTACGATGTGTTCACAGCCTTGCGTATTTACTAGCAATTGATGCTGCAAAG

AAAGCGCCAAGTTTCTTCTTGATGTCTGTACCGGCTTGTCCGTCAAACTGCCTGTTTATCAATTGTTTAACTGTGATACG

TCGTGAAATTTAGAACGCAAACTAGGATTGTCTTAAGAACTGAAACTCAAATGGTGTTCGTAACCTTAGTCAGCCAGAGT

TACCACTACGTGTTAATGCTATTATTACAAACCGAAGACAGACCTTGTAAGAAGTGACACATTTCTCCGTCCTGCTCAGC

CGGATAACTGATAACTAAGATACGAACTTAATGTACGTACATCAGATCCGACCAAAGCAATGCGCTGAAATATTCGATTA

CCCGCCTTCCGGACATTTAATATTGAATAAGGACTTGTTATTTCCGAAGCAGTTTTTTTTTTTCTTTTTCTAGGGTTCGC

TTCAAGAAGTAACATTGATAACGTTCATCTTATTCAACACAAACTAACTCAATTACGTCGCGCAAACTAACTCAGTTACC

TCGCATATGTCTGAGGATCCCCTGCGTGTATTCGTACATCCCAGCCGTCTGTTTATCTTGTTAATTTCTTTAGTTGGTCC

AACGACGACGTACTTACTGGTGTCAACGAAACCCGTCACACCGACGACGACTGCTTCATTTCCGACTTTGATGCTGTGTG

AGAGATGACATTAATAAGAAATTAGTTAACATGCCCGTCCTTCTTTACTGAAATAATGAAATTGCTTCACAAAGTAATCT

GATATCACGCATTATTAATAAGCTCTTAAGTAGTGCTTGTAACCACCTGCATATTAGAGTAATCTTCTGGGTATGTCTTT

AATCAACAATAAAGCAATGTTGTTAACTTATCAAACACAACATGGAATTTAGCACACTGTTAGGAAAAAACATTGATTAT

TTTATGTCCAAATTGTACTTCATGTAATTGGATTACCAGAAATATTCATAATAAAATTATTGTAGGTCACATTTGGTTGG

TTGCGTTTTGATGAAAAGCACTATGTAGCATTAAAGAAGGACTCTAAAAACGAAGGGCTCTGACAGCACATAATAGGACC

GTGGGGCCGCACAGAGATAAGCCTGCAAATACTAGACGGATGGCTTTATGCATTCGGTCCTGTACGGTACACGTAACGAC

GGAACGGCAGGAGACATGCTGTGTTATACTAGCAGCCACCAGGCTGACAGAAAACTAGCGACTGACCGCATTAATTCTCC

ATTTCATGACTGACAGAAATCAAATGTGAGAGATGGGCAACCGAAGGGAATCAACACGTCTTCTGATTCTGTTCTGTAAT

TATTAAGTAATGTAAACAGGTCACCATTAAGACCACGGTAAGCGCAGACGTATGAGCAAACGTCGTGGTGTGGTGTACGT

GTTTGGTGTATGCGCTTTTACATTGTTGAGTAGCTTGCTCGAATAGCCTGTCTGCCGGGATGCCGACTGTTCTGACTCCG

CCTCCCTGCTTTTATTTAATCTCTCAAGCTTAACGATATGATAATTAAAGCATGCCACGACAGTTTTCTTCTGTGTCTGT

CCTACGTTATTATTGCTGGTATGATACCGTAATTATGCATTCTGGTGAAGAGACGTGAACCTATAGATTAAGTCGTCTTT

GTTTGCATCTCCAGAACACTTTTCTTACTGGCGTTGAGTAGACTTTCTGGTTTCCTCTGTTGTATTTCGCCTCTGTGCAA

TTAATCTCAGGCATAGACCAGGAGCTGATGTGTTACGATTTGTTGCACAAATGGACGGCGCGCATACGCACTTATTCTTT

GTGTCTGTCGTAAGGGGGTTATTCTACATCACAGCCTAAGTATAACATAAATTTAGTAAGGGCTGTCAGTTGTTTTTATG

ATGTACCTCGTTTTCGGCCTCATAAGGCGCATTTCTTTCCTGAAGAGTGGCTGAGTCTTAACACCGGAAGGCCAAATTGC

GGAGAGGGGTTTATCAACTCATGCTAATTATAGGGCGCGTAGTATGAGGCGCGAAATGCTGTAATTGTTGCGTCTTTCTC

GTCGGTTTTGTTAGCAAAGAGAGGCAAGGAACATTTTTCTGAAGCAGTAATTCGTAAGTTACTTCTTTCGCCACACGGTT

TTGCTTTGCAGTAACGTTGAGTGAGTATGGGTTGACTTACGCATCCAGTTTAAAGTTCCATGTTGTTAAATCAATGAGGG

GAGCATAGGTGATCCTTCCTGTATAATATGACTCGATGACGCCTCCAAAGATGATTTCTCCATAATGCTCTCCGTCAGGG

AACCTGCAGACACACATACATACATAATACATACATACGTACATACATATATACATACATATATACATACATATATACAT

ACACACACATACATACATACATAATACATACACACATACATACATATATACATACATATATACATACATACATATATACA

TACACACATACACACACATACATACGTACATACATACATACATACATACATACATAAATACGTACATACGTACATACATA

CATACATACATACATACATACATACATACATACA

>contig_22030

TACATACATACATACATACATACATACATACATACATACATACATACATACATACATACATACATACATACATACATACA

TACATACATACATGCACACGACGTCTGTTGCAGATGCTTTTAGAAGAAGATTCACAACTTAACCTTTTAAAGCCTAGCGG

TAACCTTGCTTACCACCAGGTTTAACATTCAAAAATACTAAATGTTGCTCACATTGCGTTTATGTGTTTTGTGCGAATCT

CAGAACAAACAGCAACTTTTGCCTTATAAAACGTTAAGAGATTGGTTTTATATAACTGAGGTGGAAAATGTTTAGAGCGC

GGTACGCACTGAGTCCTTATATAAAACAGGGTACATCCCGTCTTTAAAGGGTTAATGTGCCTGTGTTTAGGCATGTGTAT

ATATGCATGCGTGCATATATACACATGCACATATATATATATATATATATATATATATATATAGCTGGTATGCGTACGAG

TACGCTTGTGTTCCTATAAATGTCAGTATGTATTTTTCGTATTACCACGGGCAGAACGGAGGCTGTCGCGTCATTAAAAT

TCCTAGACAGAATTAGTGAGTGACGTGAAGTATCCACTCACCTGCCGTGATGAAATGAAAAGACGCCGTTAATTTCTCCT

TCATCGATGAATGTTTCGAACACTGTGGGCAGATTAAATGCAGCTTTTCCTGATGTGGGGCGTCCCATTCCCGCAACACC

ATCAGCTCCCAGGAGACAAAGCTGTACTGAGACGTCGGTAGCGAGTAAAAACCCTTGGTCCGGTGCAATCAGAGTGCGGA

TCTGACAAAAAAGTAACAAACAAAATACTGCCGGTTTCTGAAGAATGGACTGTGTTTACGAGTGTTACCTAATGTACCAC

GCTGCTCTGCTTTCATATCATCTTAATTTAACTTGCTGAAAGTGAAAATTTAATTCAGGGAATTGTAAGTCTGTAATTTT

TATTTGAAAGTTAGCATATAAACTGAAATATAACACACTGAGTAGTGATTCCGCGTAACTTCCGTATTGAGAGGACATCT

AGAGGTCTTAAGCTTTTGTAGTTTATTTTTCGCACTTATGTTTGCAATTTATAAAGCCTCCGGCGGTACGCACGTCTTGT

AACGTAAATGATTGTCTTCCAGTTTCCTTCCTTGGACCTGCTGTCGGTCGTGCGGTTGCAAGGTGCCATACGGTGTGCTG

CATGCGCGTTCTTTCTTTGTACTCACAAATGATAAAATCATGCCGTACCGTAGCAATAAATAATAGCAAATCTGCGTCAT

ATCATATTCTTAGGTATTTTTTACAATTAGAATTACTGAAAGGAAAGAATCCAGGAATTGGTTTTTGAAATGCACTATAT

TTTAATGTGTCGTTACGTTTTCAATTATTTGTGTACAGATGATTTAGCAATTTTCGGCACAAAAAGCCGTTACATTAGTA

CATTTTGATGATACTGGTTAAGGCTATGTACAGTGTTATGAATACTGAGGGCTGACAGACACAAATTTTGGACTTGTGGC

TGTGCACCTGTACAGATATAGTACGCCAGCATGGTTATGTTTGTCTCAAAAAGATTTCTTCTGTGAAAATTTGTGATTTT

GTCTCATCAATATGCATTGCCTACCAAACTACATGGCGTCACCTCTATTATAAAGGAGGAACTGCACTTTTATGTCCAGA

AGATTGAGACAGCAAGTTGGTACATACAGTATATATCAAATTACATGGCTTTACTTCCTCCATTCTCAGGACGGAGGAGT

GCTGTTCTGTCCTAAAGGTGGAGTCAGCAAGGTGTTCCGAACTTCTGGTACATATCTATCAAATTACAGGACGTTACATT

TAACAAGATACTGCCAATATTTGTCTTTTGATTTTGTGCGTCCTCTTTGAGAGGTGATTCCTGAAACAAGATGTGACCTA

CTGCTTGCCCAAGAGCTGTCTTGGCTGAAGTGGACTATAAATATTAGCCAGGTGAAGCCATTATATGCATTTAGGCTGCG

GGTTTCACTGGGGAGTAACACAACATTAGGAATTATGTCTGTTGCTCGTATATTGGAGAAATTACTTACTGTGACATTTT

CGTGGGCTTCATTGCCGCTGGCGTATGCAGTATCGAGTATAGACACATTGGCCACTCTGTTTGAGGGTCCGAGTCTGGTG

GAACGGCTGGTCTGGTACTTGACGATGCCCGCACATTCTGCAATCAAAGTGACAGCAGCGCGTACGAATACCTGCCTGCG

TTCGGTGACAGACTTGCAGGAGCGTTTTCGAATAAACAAACAACATATCGAATTCTTAGAATTAAATTGGCGCCTATATA

AGCCACTCGAAATGTAACACACATGGCAGTACTAGGTGTGCCTTTGGTAGGCTTGTTGGTAGCGGGACCTGTGCGACGAT

TCGAGAGCGGACGGAATAAGCAATAGTCTATATGTAAATAAGTTATTTTTATATACTAAAAACGTAAATACTTAAATAAG

ATAAACGTGTACATTATTTTTATATACGAACTATAATGTAGACTATATTGAGATTGTATTTAATATTTATTTACTGAATC

TTGGTTGATGTATTCATATTTAACTAGACAAATAACTAAATGAATTAATTGATATTAATTAGTATTTATTATTTCCATGT

TTACTTGTGGATCATTTTCCTTGCTTTTCCGTTGGCAATGCCAGTAATATTTTGTGTTTTTATCTTCAGCGAGACTCCCT

TCCGTTACAAAAATGGGAATTAGTGAATTATTGATTGTACAGAAAAATCCCTTCCCAGGCTTGGAAACACATGAAGTTCG

TAATTTCACAAAATTGTACACATACCTGTCACCAAGACGTACACTATTTTTTTTTGCATGGTACCGTGAACTGGAAATAT

TTTGAGAGAAATACTGATGTTTCATTGAATGAACGATTTAACCTGGCACATCTTCCACTTAACAGTTGGACTGTTTCTGT

CTTGCCCTCTTTATTTTATGAAAGACTAAGCGGTTTTAAAATTTAGACATGACAACAAAACGGACTTCCCAGGTTAGAGC

GACACTAGCGGAATTGAGCTTGGAGTTACGACTGTATTATGAACAGAGATAGGGCTGAGTGGTCGGTATAAAACGGAAAA

TAATGTCAAGCGCGGGTCAAGTAAATATATTTCTTTAGAATTTATGAGTTACGCTTTACTTTGCTAGGAGTGTAACCTGC

TCCTAGCTGAACATTCGACTTAAGGACATGGAAGGCTCTATCATCAGGTGTGCTGCAACCTACAGAGGGCGACGGCTTGA

TGAGTTAGCGTTACTGTACACGACTCTTCCCAGAAAGGGTTTAATGGGTCAGTCGTCAAAAAAACCGACAGAGACATCTG

CTTTGATGTTTAACCTTTTAAAGCCTAGCGGTAACTTTTCATACGACCAGGTTTAACATTAAAAAATTCTACGTGGTGCC

CACATTGCGTTTATGTGTTTTGTACGGATCTCAGAACAAACAGCAACTTTTGCCTTATAAAACATTAAGAGACTGGTTTT

TATAACCGAAGTGGAGAGTGTTTACAGCGCGGTACGCACTGAGTCCTTATATAAAACAGATACGCTTCGTATTTAAAGGG

TTAATAAACAAGTGTAACTTACGGCACCCTTCGCTTATGCAGGATTCGCTGACAACAACCAGGTTATAAGATGAGGTGTC

GAACACAACTTCGAATTTTTGGTCGCCAATCCACAAGTCTCCGACGTACTGGGTCTGGGTGAAAATTAATTGCTTATTAA

TTACAAGGAAACAGAGTAACTTACGATATGAATTAAATATTACATTGTGGTTTAGTTTAATTGCGAAAGGTACTATTCCC

TGTACTAGGAATGGCACTTGTTCGGTTCTCCCTGTGTGACGGGTGCTTTTCTTAGAGAAAGCACACTGCATTTAGGATTT

TTCTGAGCCAGTGTTCGTACCGCACTGAAAAGTGGGGTTTCTCCATATCTAATTAAATATAGGTACCAACCGCACAGGAA

GAATGAAGTTCCTCCGCGTCTAAGGAATGGCGCTAGATTGTGGTTTCCCAGAGAAATGCTTGGATGATCGAGTTCTGGTT

TTGGGTGCCCAGTACCTAATTTTCGAAATATAAACGCTGCTGGTTTTCCTGGTATTATCTCTTAAAGCGGAACGTGTGCA

TTGACTGCCTTTTCTACACCGAGCCGTTTTGAAATAACTGGTTGTGACGGACAGACAGGTGACGGACACTTGGCTATTAT

GTATATGTAATACTGCTTTATGTCTTATATATAGGCTATAATCCCCTAAGAATAGACGACAGTGTGACTGGGTGAAGCAT

TTTATCTGCCAGCGCATCATACTATTGAAGTGCCCCCCCCCCCCCCCATGCCCGAAATTTGTGTAAAGGGACTAAAGATC

ACGAAAGGCTTGTCAGGATAGGCCGTGGGCTGCCGAAATTCGAAAGGGACATTTCCAGAATACAGTCTAGTACGCTACTA

CTACACACCTTTGCAGGCTGAGGGGCTCAGACTTTTAATTTACACCAAGGGAACAGCGAATTGAACGAGTTCGGGAACAG

TTACCCTCCACCCTCGCTGGAGTGTTACTCGCCAGCATATGTATGGGAACTATGGTGAAATGTTTGATTTTGAGTTCGTT

TTATGACTTCGTAAATGTTTTACATTTAGTAAGCTTTATTTTAACTGCTAGCTAGAAGCATTGCTGTCTTTTGTTCGAGT

GCTGCTGATATTGGGGTATATCTTACCAGTATTACTGCTAAAGGTATTTATCGTAGCAGTTACTAGTCACGTTATGGTAT

GCATTGTTACTAAAAACAATGCCCTTCAAGCACCGTGCATGGCTTTAAGATTTCTGTCAAGTTGAATGTCGCACAGACAT

TTCCTTGTGTTCGTAAGGGTGACAAATTCCCCTCTGTCACCGCACAGTTGTTGCCGATAAAGTTGCTAGGAAAACACCTG

CTGTTTTCAACCAACCAGCTTACTTGTACTGAGGTGGTACTGGCTCGAATCTCGGTCGGTACAGCTACTGGCCTGACAAA

TGCCTCTCGTGGTCTTTTTCAGTCCCTATAGTCAGTGTTGTGATAATATTTTATGTTACTACCACTTCCTTTCATGTCAG

TTCCGTTTCACTGTTGACTGACATTGTCACTTGACAGTGTATAGCCCAGTTAGATGTCTGTAAATAAACTGTGAATAAAC

AGGTAAATAACACTGCCAGTACGCAGCGTGTCTTAAATAAGTCGTTCTGAACTGCAGTTCGGTCAATATAGGGACTGGAT

GACTGGAGTTCGACACCCGGTGGGGAAGTAATTTTTCTCTTCTCCATGTCAGAAGGACCCTTGGGACTTATTCAGCCCCC

TATTCAAAGCATATAGAGGCTAAAGCAGACAAGGTGTGAAGCTGATGCTGAGGCTAAGAATGTGTGGAGCTGTAGCTCCA

CTTCTTCGTACGGCTCGTGGCGTGTTTTTACTTGTTTGAAAGAGGTGTACGTGATTTGAATGTAATGCCATTCGTAACGA

AGAACACCGTTCAGACCTGTTGTTATTCAATCCAGGGCATCCCGTAGAGCCCAGTTTTTATGTCACTGTCCCATCACTTG

TATAGGACACGAATATATGCTAGCTGTTGTCAACTTACGTTTATGTATTCGGATACAATTTGTTTCCTCCTCAAAGGAAT

CCTGTAACAATACCATGACATTAGAATAGGTTTAAATGTGTGTGTCATGAAGCTAGAAATTGCTACTGCGTGATTCACCG

TGTATGTTTATTATTTTAATTACATATCGTATAGTACTCATTATACTAATTCCCCTGTCACTGAATAGACACACGCACGC

ACCATTTTGGATTGCGTTGGTACTGTGGTTATGTACACTGTTGTCGTCCTCCTCTATGCACAATGACTAATATTCAGACT

GGAAAGTCTTACGGGGTTTATTTCCAATGGCGTGAAAACGTTTAAAAAAATAAATGACCTTACTGGATATTGTAAATTGT

CGGCGCATTTATTGTTATTGAAGGATAAAATCTCATTAACGCATTCCTTACATTTTCAATAAGGAAAATGGAATGAAGGA

ATAAATGAATACGGACTTATGTGTTCTGTGAGCAAAATTAAGTATCACAAACTTGTGAAAATGCAATTGGGTAGCAGGAT

TGGGTAGGCGAAGTTATCGCTCTGAAGCACATGTAAATTAAATGAGCATTAACGTGACTCGCGGCTGGCGTGCTTTAATT

TAGAGTTCCGAATAATCAGGTCTTAAAAGTTTTAATAGAACTGTTCATGTCTACGTCGTAACGTAGCAATAACTTTATCT

GTAATATCACAATGCATCGTTTAAATTTCGGTTCACAAGGAAATTATAAATAAACTGATGTTATCTGAAGATCTCTCGCC

TACGGATTTTAAGAATCTGAAATGAATAGCTATAATGTTTTTCCGCTCCGCAATTTTGTACGGACACCGTTTTGGTTTTG

TTAATTTGGTGATGAAAATAGCAAAGTTAAACGCTGTTCCGTAGCACTGTGCTGATAAGCTTCATGAAACTGTACACAGA

ATTCGTCCTACTGAGAGGACGATATTGTTATAGGTCTTATAATTACTGTTTGTGGCTGAGTGTGAGACTGGTCTAGACAG

ACAGACAGACAGCCAAGCAGACAGACATACAGACAGACAGCCAAGCAGACAGACATACAGACAGACAGCCAAGCAGACAT

ACATACATACAGACAGACAGACAGACAGCCAAACAGACAGACATACAGACAGACAGCCAAGCAGACATACATACAGACAG

ACAGACAGACAGCCAAGCAGACAGACATACAGACAGACAGCCAAGCAGACAGACATACATACAGACAGACAGACAGACAG

CCAAGCAGACAGACATACAGACAGACAGCCAAGCAGACATACACACAGACAGACAGACAGACAGACAGACAGACAGACAG

ACAGACAAACTTAAGCTGGATGTTAACAGGTTTCAGATGAGACACGCTGTTCCTTTGGCGAGACTTACGTATGTTGGGCG

CAGACAACACAGAATAAAGCCGCAGCTAAAATGGAACGCACTGTCAACTGCATGACGACAGGCGTACGATTCAATCAAGA

CGTGGTGAACTGCTCTGGGTTGACTAAATGGGGCCTAGCGTGCGTTTAAATACGTACCTTATCTCTGAACCGTAAAAACC

AGCTTGTGATTAGTTTCAAAGGGGAAGCCTCTGGTTGTAGGAAACAAACACTTGAAGGTGAACAAGGCGTCATGCGGCTG

TTGTTTTAGTGTGGTAATTCCAAACACAGACCGGAACGTTGAGAGGAAATTCGATCACATAAAGAGTACATGTAGAGGAG

ACCAGCTGAACCGATATGAGTACGTGTATTCTGTATAAGAGTCCTTTCGAGCATAAACTATCGGTATACCAAAATCGGTT

GCTTACGTTGAATTATTGGCTCCATAACCGAAAGAAAAACTAAAGTAAATAAGAGTCCATTAAATTTATCACCAAAGTAT

TGGTCCTAATGTAGGCCAGGCATTAGATCTAGTCACAAAGTAATCGTGAGCCAGCACGGTCAAATTAAAGGAAAAGAACG

GTGACAAAACATTTAGGTACAGTGGCCCGTTTCAAAACTGTTCAAAAAGGCAATTATAGAGTTTTTATTCAGAACCAGTA

CGTAATAATAGCAGTCATATAATGTCTAATAATAATCAGACGACATCGGAATATAGCAACACCACTGTGACAGACTCGCA

TTTAGTTATTGCCGGAAGTCGTGCTTAGGCCAAACAGGTGGTAAGCAGACTTTGCATGTAAGCAGTGCGTAAGAAGTTTA

AGGCAAAATGAAAGGCATTTTAGTTTATGAGCAGCCGTACAAGGAAACTTATATTATACTTTTTCGACGTAGCTTATAAT

ATATTCAAAAACAGACATGACTAGGCAGACAGCTTACATTATGAAGGATGTACCTTCAGTTGTTCAGGGGACGCTTGCTT

GTCCAAGAGCAGGCACTGGTTTCGTTTCATAGTCCTTACCTCTTCTACCTACTCACAGTAGGTGTCGCGGCTGTTTGTTA

TCACTTGATCGCACTCAGACACACCACAGTTGGTAGGACTTCTCTGGACGAGGGATCGGACCGTCGCAGAGACCTCTACC

TGACAACACAAACACTGTACAAGACAAACATCCATGCCCTCGGTGGGATTCGAACCCACGATCCCAGCAAGCGCTAGGCC

GCAGACCTACGCGCTAGACCGCGCAGCCACTGGGATCGGCGCAGGCACTGATACTATCCGAAATATCCGAACTGGTTACT

CAACTGTCAGCGACGTGTACCTTACACATCATTATGTCGAACTGAATGATTTAAACTTAAATTTTCTACGAAATTAAAGC

ACAGTAACATTTGTATTAATTAAATTAGTACTTGAGGACATTATCCACGTAGACCTGCATTGATCAGAATAAGTAGACAA

GGAATGAACGTAGGTTTACAAGTATGTGATAGAACTAGGATGTAGGAGAAATAGTAATAATAACAATAATAATGTGATTT

TTAAGACAAGTACGTACATCAGTTACCAGTTACGCGAATCTAGATTTTACATGCTCGTTTAGAAGAAGAAGTTTGGTGAT

GAAACGGTACGGGATCGCAGGTGAGTTACCATCTAGACTAAGAATATCGGGCGGAAAGGAAAATGGTGAGCACACACTTA

AACACGAAGAGCACAAGAATGTGTGCATTAGTATAAAACACAACGGTGTGATTGCCAAATAGCCTCCAGACCAAAGAAGT

CCGATATACGGTATTTTGGAACAGTTCGCAGGTGTTCATGAGTCGTGTTTGGGCAGCGCGATGTAACACTTCAACAGCAG

TGGGCATTACGGCCTTCAATGTGTACAGAAGACTGGCAACAATGATACAGAATTGTATGATAATACAGCCTGTGAACATG

TAAGGCAGTTTGTAGTATTATCGTCTGAAAGAAACGACGTGGAGAACACGGACAGGGTTCTGGATGTCATAATTTTAATG

GTAAACCCATATGGCTGTGCACATGAAATTTGGATCAGAATACAGAGAAAAGTAGCAATTTGTTTTGACATTGTGCTTAA

TATATTTATTGACCAAACGTGTGTAATTTTGTGTGATGTGTGCTGTAAAATACCTGTGTCTAAATGCTATGACTTTTCCT

TTTTGCTGAAGTGCATCAATATACTTGCTGCAGGATGTCTTAATTTTCGTTTTCGAAATGAACCTGCTGCGATTAGCGAA

CACCGATAAAGACACGGATGTGGCGTGGTAGCTTTCTTTCCATGAATGCGTAATTTTTGCAGATGTCAGTCTTATCGTGC

TTTGTGACGAGGCGACGTCCGTCTCAACCTTCACTCGACTTTTCCTCATTGTGTGTTGGGACTCGCCATGAAAAACCGTA

TTCGATAAGGCAGAGTGTGCAAAACAGCCGTCTAGTTTGGATAATTTATTATGTGGTCGACCGTGACACAAATTATTCTC

AGAGTGAAGCTCGTGTGTACCTTGAAAAATGTAGTAAGGCAGCCAGGATACAGAAATAAACGCCAAAGAAATTAAGAACA

TGTTTATGTCCAGTCACCAGAATGGAAGAAAGAATGATATGAGTATCGTCATAGTCAATATAACCTTCGTAAAGGCGGAA

ATTTCAAATGCTTGTTTACGAAAGACATTAGCACATCAAAACTGTACCGGCAAATGAATTGGAGATTAAACTGTGCAAGT

GATCACTGCCCTTCAGCGTCGAACCGTTTATCTGCACTGCTCGTATCCACTACATTAAAACGTAAAGGAATATTTTTCCT

GTTCTTTTAAACTTCCGGTGGTTGATGAAATGTGGAAAGTTTCTTGGCTAACTGAGGAACTGTTAGTTTTTCTGTAAGGA

CTCTGCTCCATGGAGTAATTAGCTTTAAATGCATGTAAAACTTTGTCATCCTAAATTCTCGGTCGCATTAGGACACATAA

GTGGAAGCAGCCGGTACACCACAGTGCACTTGCCACTCACTGTCCTTGTGTGATCTAAAATTTTCGAATTATTTTTAAGT

AGAGAAGGGTGTCAGTAGATTTCAAGCAGGGACACACTCCTTTAGTAGCCCCTTGCAGTTCTTTTGGCGAGTTGAAACAG

CGCGTGTCCCTGCGCGACCCAGCAGTGCCGTTCGTTTCCCAGCCGCTTGGAATGTCAAATATCAAAATGTCCAAGCCAAA

CGGTGAAAATGTAACTAATTCCTGGATATTTACGATAAGTTCGCGTTGTTGATGGGCATGCGAATTAAAAACAGGGAGAA

TTTGCTTCCAAGAAATTACTTTTTATTATCTTGGAACATGGTGCCCATCGTTTCACATATGATGTCTTAAAAAATAATTG

CACAGAGAACAAATCTGACGAAAGTGAAGCAGCAGCAGAAGTAGTTAACAAAACAAAACTGGCTTAGTTTACAAAAGCCG

ACGCTTTTTTAAAAGACGTTACCACGACTAAGATATAAATGTAATTACGTGTCTCATTGAATGTTTGTAATTTTGAAATT

AATTGACTACCTTAGTACAAAGACTGTTAAATACGATTTTTCTAAATATGAACTTTTGTATCCCCTGAAACGAAATGTAT

TTTAACTGTTTACGTTAAGTCGTGCTACCATGATCTTTAACTCAAACTTTTCACTCGACATTTTAAAGGTATGCTGTCTC

AGCAACGGTCTTAATATGTGCATTCGTGCAATGTGTCGTAGTAATTGTTCGCCTGTTGATTTGGTTGCTGTGGCAAAGCG

TGTTGTTCAGACGGAATCAGCACATGTGTCCCGAAACTGCCTACCGTCCATGCACTGCTCATCAGCACGAACAACAGCTT

TCTGGGCCATTTTCTTCTAAGTGTCAGGTCAGGCTTCATTCCACAAACGTCCAACTTTGACCAAAAAGGAAGAAAAACTT

AGTAGCATCAGGCCAAACACAACTACAGTCGAGCTCGTTTATAACGTCATGAAACGGACCGAATATCTTGTGTTTTATGA

ACAAGTGTCGTTCTAAACCGATGAGTGTAATGCTATGGTTAACAGTGAGGAACTAATTGGTACCACAGAATGTCTGACGC

TATAGACGAGGTGTCGCGTGAACCGGTGTCGTTATAAGCGGCGTAGACTGTATTTATTTCTCAAGGAAATCAGCTACATG

TTTCGGCTAAAATATGTACATAGCATAAATATATATGCAGCCTCGGAATGGAATTTTATAGAGATTATGAAATTGTATGC

TTGTGTAAGTGTTAAAACTATTGATAAATTATATTAATATATAGTGATGTTAAGATGTAGTGTTTACAAGATAAAATTAG

GGGGAAGCGTCAGCAATTATTTGGAGAGACAGGCATATCTGCCCAATACGCAAAATGATTCAGTTCCCCTTAAGGGCAGA

AATATTTTTTCGGTCCTTCAGAAGTACCCTCACTACCGGTTAAGTGACGTAGGTCAGCAGGTCTAGTGTTAATTCTTTTA

AGAATGAAGTTGACCCTGTTTGGTGAAATAACTGTTGTTTATTGTGAAAATCATGAAATACAAACACGCCGTACGGGAAC

AATGCCGAGTATTCGTGAATGTTAAACGTAGTGTGTGCAGTAACCACGTATTTATAGGGTTAAGCGACTATTTTGCCTTC

CGTCTGGCTGAGACCAAAATAACTGTGTCGCTGATAATTCATTTCGGTGCTGTTCAATATACTTGCACTTCATGATTTAA

ATATAGGGTGTACAAAACTGACAAAAGCCCGTACACTCATAACTTACCAGCCTTTTCCTTCTGACTCGCAGTAGGGTGAC

GCAAGGTCCAGAGTGCATAAGCCAGTGTTACAGCCATCGTTAATCATGCGTAACGTAGGGTACGAATCCACCCATGTACA

CTAATTAAGTCCCTTGTTGCAACGTTACTTAGCAGGACCGGAGGTCGTCTGACCATGAGCTGACAGTGTAGGTTTGACCG

AACTGTCCAGTACGTGAGGAGGTCTGACCTCTTGTCAATATTACCCAAGAACTCAGTAGCTCGTGGGATTAACTGCGCCA

GTAACAGGGACAACAGAAATTTCAGGCGGAGTTAGGACCTCAGATGATGTAACACACATACTAGGTGCACCAGCTTGAGA

ATAAAGTGCACAGGAAGGTATCTGGGTACAAGATGCAACAGCTTAACTGTTTATTGAGCAGTTTAGGGCGTTAAATAAGG

AGGTTTTTCAAGCGTTTTACAGGTAGCAGTACTTTTAGGGGCGAAATCCAAGCCGCTTAAATTTGCAGGACCTTTTGGTA

TGGCGGGGCACTCAAGGACTCTGTGCAATATTGTGGTAGGAAAATCTCTTGGCAAATGTGCACTTACAGCTTGAGGAAAA

TATTGGCGTTATATGTAACATTGGTCAGTGCTGCTTCGCGTTCTTTAATTGCTGATGGAAGATTCTGCAGTCATATATCA

AAAACAGATCTTAAGTGACGAAGTGCAACAGCAGATAAATGGTCTACCGTCGGGATCGTACAAGTTTTAATATTAATTAA

AATATTCCAAAAGTATTCTTTACACGCCTGTGTTAATAAACATGTCCTGTGTCAGAAGAAAGCGATGTAACAATAACATT

TGTGGGCCTAATAAATATCTTGTTACAGAACAGGTTTACGCAGAAACGCTTGTAGGCGTTCATGTTAAATGTCCGGTACT

CGGCGCGTTTGCGTAGTCGCGAAAAGCGCCCGTTACCCTCGTAAGGTCCGTCCGTCCGTCCGCTTGTATCATCAAGGCTG

CTACTGTACGGATTTCCGTGAAATTTAATACTGGGAACTTTAATGAAAATCTGTCGAACAATTCCAAAATTTAATGAAAA

TCGGGCAATAAATTCGGGTACTTCACACTAAGACGTCAGTACGTTGTTTTGTTGTTGGCGTTATTATATCACCATACAAG

CGCTCTGTTCCAGTGAAATGGCACCAGGCTGTTAGAACAGCCGAAGAAGTCTAAATATTAGCAAATGCGCCACAATGTTA

CGTTATACCGACTCTGCCTATCTTCTTATTAAGTCATCTAACAAGAAGGAGATGAGAGGATAATATTAAGATACGATTTT

GGAATGTAGGTTATGAAGATTGCAGTAGAAGCAACTGGCTGTGGATACTGCCCGGGTTTAGGATCTGATGCTAGATGTCT

TGGCTGCGGGTTTGCTACCCCAGGGATATTTATTTAGGTATAGGTTCTGTGATATAAACAACCTAGATCACACTACAGAT

TTTTATTTCCACGTTTTTTCATACATAAACATTGCTAAAACCTTAACTCCAGTAAGAGAAATCCTACAAAATTTATCGAA

CCTTTATTATACGTCATCAAACCCTACGTACTATATTTGTGTTAGGTTTTATAGTATTATTAAAACAGTTATGCTGCTAA

ATCTACATACTTAGTAATGCAGTGGCTTTCTAAGCGCATTTTTTTTAGCTTTCATTGTATGGCGTGGTGTTCTGGAGGGG

GGTGTATTACGCCGTCACAGTGTTACTTCACTTCCACTTTGCTTACAACATAAGGATTACACACATAACGAACTGAGTTA

CATGATATGCAATAATACGAAACGCACGACGCCCGAGGAACTGTGGTCCACAGGTCCGTCTTCATGGTGAAAATTCTAGA

ACTGCGTCACCGTAGTAACATACGACGATGTAGTCTGTTGCTCTAATCTATAGCTAAAACCATGCAACAATATACGAGTA

TTTTACAAATGTTTAAATGATATAAGAGTCGGTAAATAAAATGTTAATATCTAGAACTTAAGTGGAATACGTTTCGTTAA

GCATTTTATAATTAGGGCCATTATATTCCATATCGTACTTTACGGTCTTAATGCTTGTCATAACCTGTAACCTGTTTGTT

TGTACATGATAGGGTCTGTGTTCTAAACTTTGGTTTGTTACTTGTGAAATAATTTAGCTTATTATAAATACTGTAGACAA

CTGTAACTTTATTTTAACGATGTAATTATTCGTAGGTGGAAGTTTCATTTTTCCTAGTGGTAAAGTGGTCATCTTACCTT

AAGATATAACTAGGCTAATTTGATATAATTTTGTATATAATTTCATTAAGTGGATATGAAATGTCTATTATAATAGCATG

TCTACTTTTTTTTGTACTGTGCATGCCCAGACATGTTTGCTGTAGAGCATTATAATATTATTAAACAAAGCAAACTACAA

CGTAATAATCTAACTGTTGTTTGTGAAGATATTTGTTAGTGTGATATAGGTGTATATATATTTTTTTAGTAAGTTTACTA

GAACTAATAAACTCAGTTTGCTGCCCTATAAAGCGATGAACTAAATAATTTATGTAGGTGCTAAATGCATGATTACACAT

GTGATGGTGAGAAGTTTAACTGTCTCACAAAGTTGTGTATCTTTTGAGCCGATGCACTCTTTTGAATAAACCTGCTAAGT

GATGCAAGTTCCAAGAGTTTGCTGTATGCAAATTGATAAACATTTTTTCGAAAATAGTATAATATCACTATGCTTACATA

AAATGTTATGTTAGTGAATACATAAAACATCGGGTAAAAGTTTATTTTGCCTTTAGAAAAAAATTATGGTAGTTGCTGCT

AATTCCTTGGCATTTCCACTCTTTAACTGTCACACCTTCGACAGTGTGTGTTTTCCTACAGTAAATGTAACATGCTGATG

CAGATGCAGATGCAGTTTCCCGTCATGACAATGGACACAGTTTAGTAGACTGATGTACCGAGGCTGGTTGCTTGTCATAT

CTGTGTCTCTGAGCTGCCTGCAGGTGTCGGTCGAATAATACAGTCTGCTGCTTCAGATGCTCTGACACTGGCCAGTAGGC

CGCTCTCTGCAGCATCCGCGCCGCGTTCTGCGTCCATGCGGTCGAGCTCGTCTTTCGATAAGTGGTACACAATTCCGGCC

CCATACGCGTCTCCGAGTACGTTTATGGAAGTTCTGAGGCGATCGCTAAAAAACAGCATCAGCAAAACATTTGAAATACA

AATAACAATGGCAAGGGTAAGAATTTACCTCTGTCCTTGAAGGCGCATTTTCACTAGTGCAGTAGGTGACAGTCAATGTA

CGTACAAACGTAACATTACTGGGCGTTCGCGTGACGATTGTTGCCGTTGAAAAGCAATAAGTATTACATATTCTGAGTGT

ATGTTTAGGGCCTTAGTTATCCAGCATACCAAGCGTATGCGTCATACTATTTTGTCATCTGTTGCCTGTCTGGTTCTACC

ATATTTTTCCACATTGGCTTATAAACGGCACGATTTTCGAGAAAAAGTTACTGAACATAAAATGTGTGCTTGGATTTTCT

CCACAACTTTTGTATAAAACATTTCTCATTCAAAGAAGAATTGAGCGAGGTATCATAAATGCGCGT
