# Supplementary figures and images for: Cross-reaction between Formosan termite (Coptotermes formosanus) proteins and cockroach allergens
Source: PLoS One. 2017 Aug 2;12(8):e0182260. doi: 10.1371/journal.pone.0182260 (PMC5540505; doi:10.1371/journal.pone.0182260)

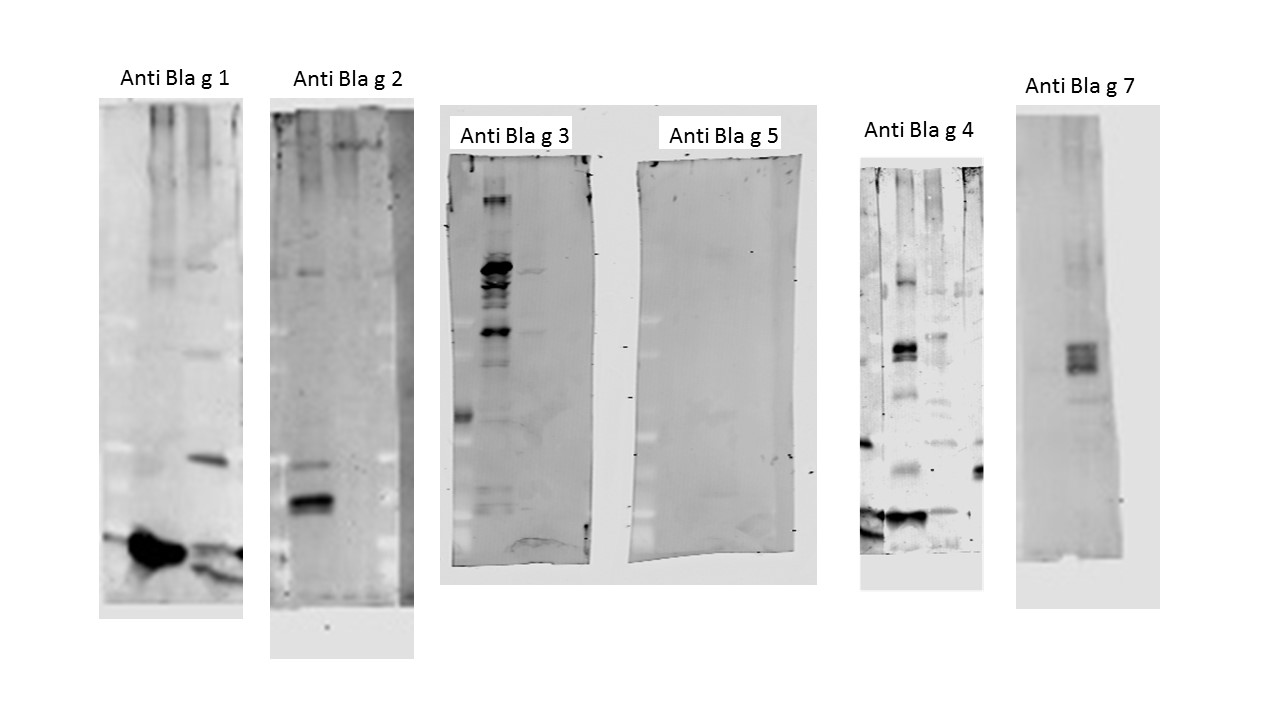

Supplement: S2 Fig — (JPG) [file pone.0182260.s002.JPG]

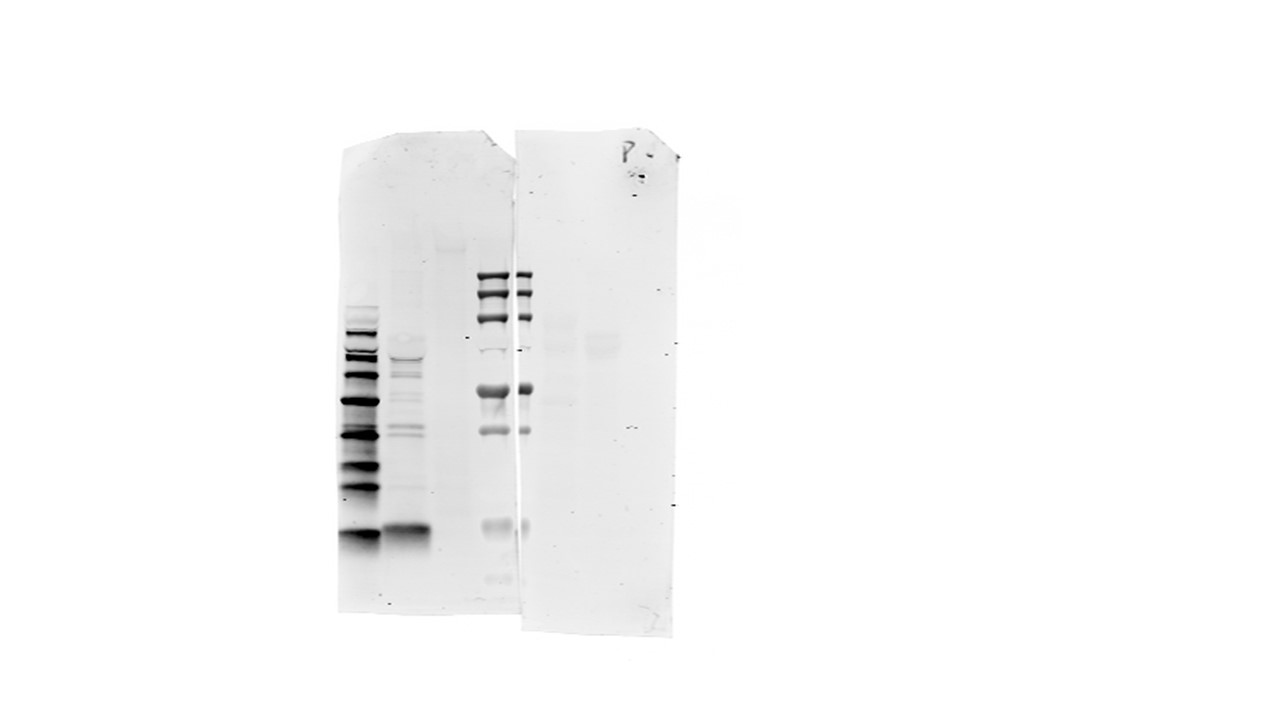

Supplement: S3 Fig — (JPG) [file pone.0182260.s003.JPG]

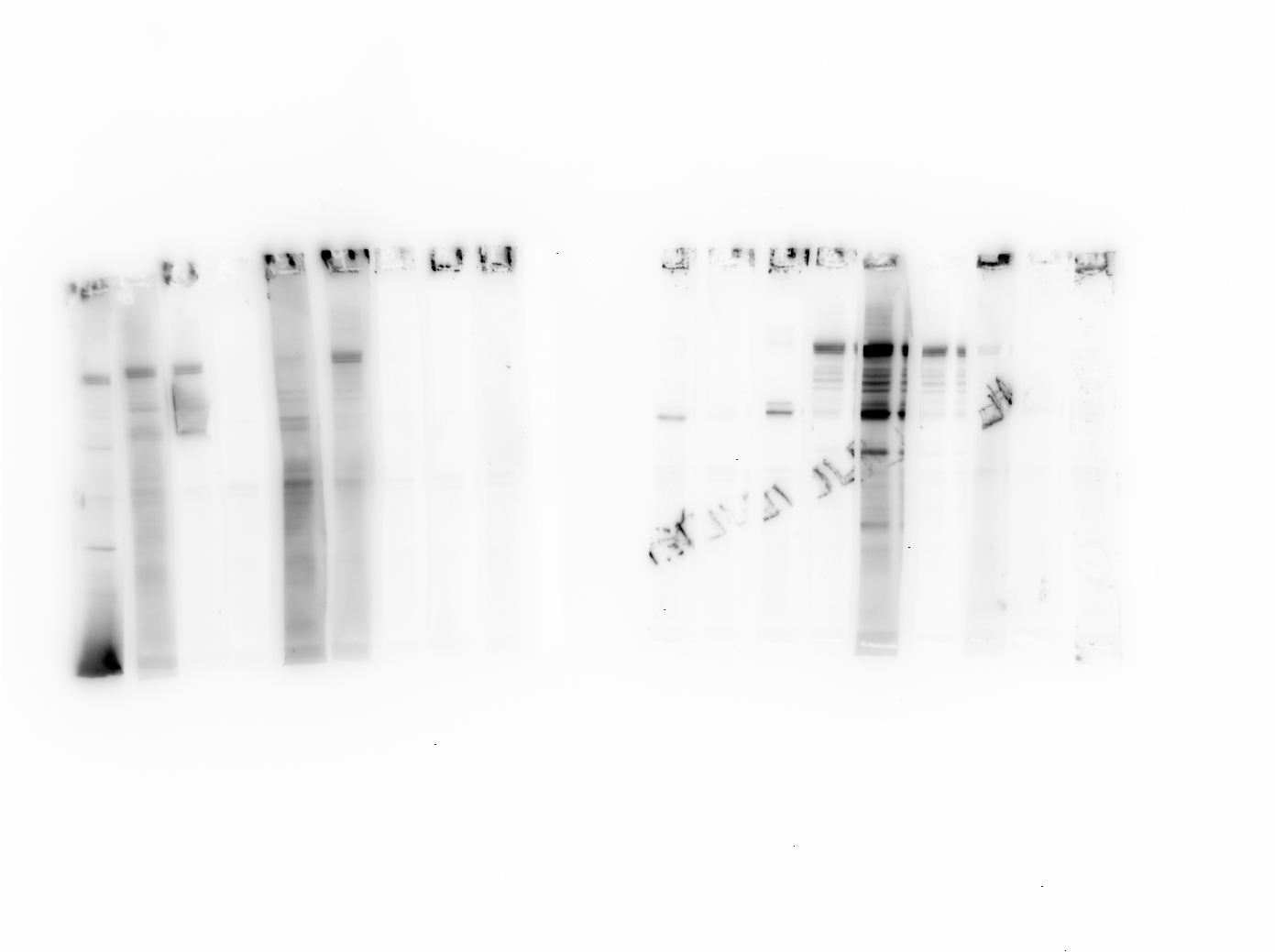

Supplement: S4 Fig — (JPG) [file pone.0182260.s004.jpg]

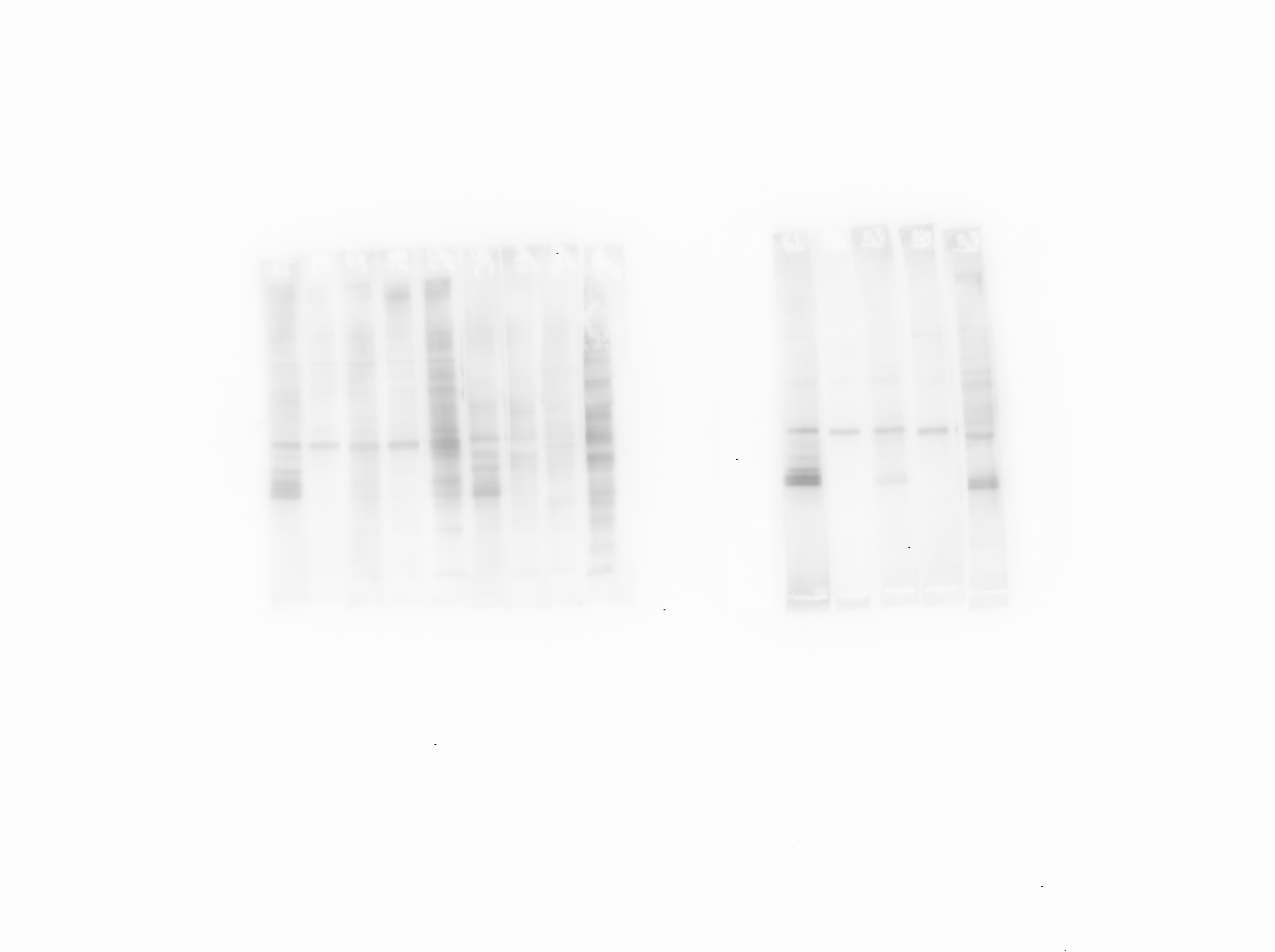

Supplement: S5 Fig — (JPG) [file pone.0182260.s005.jpg]

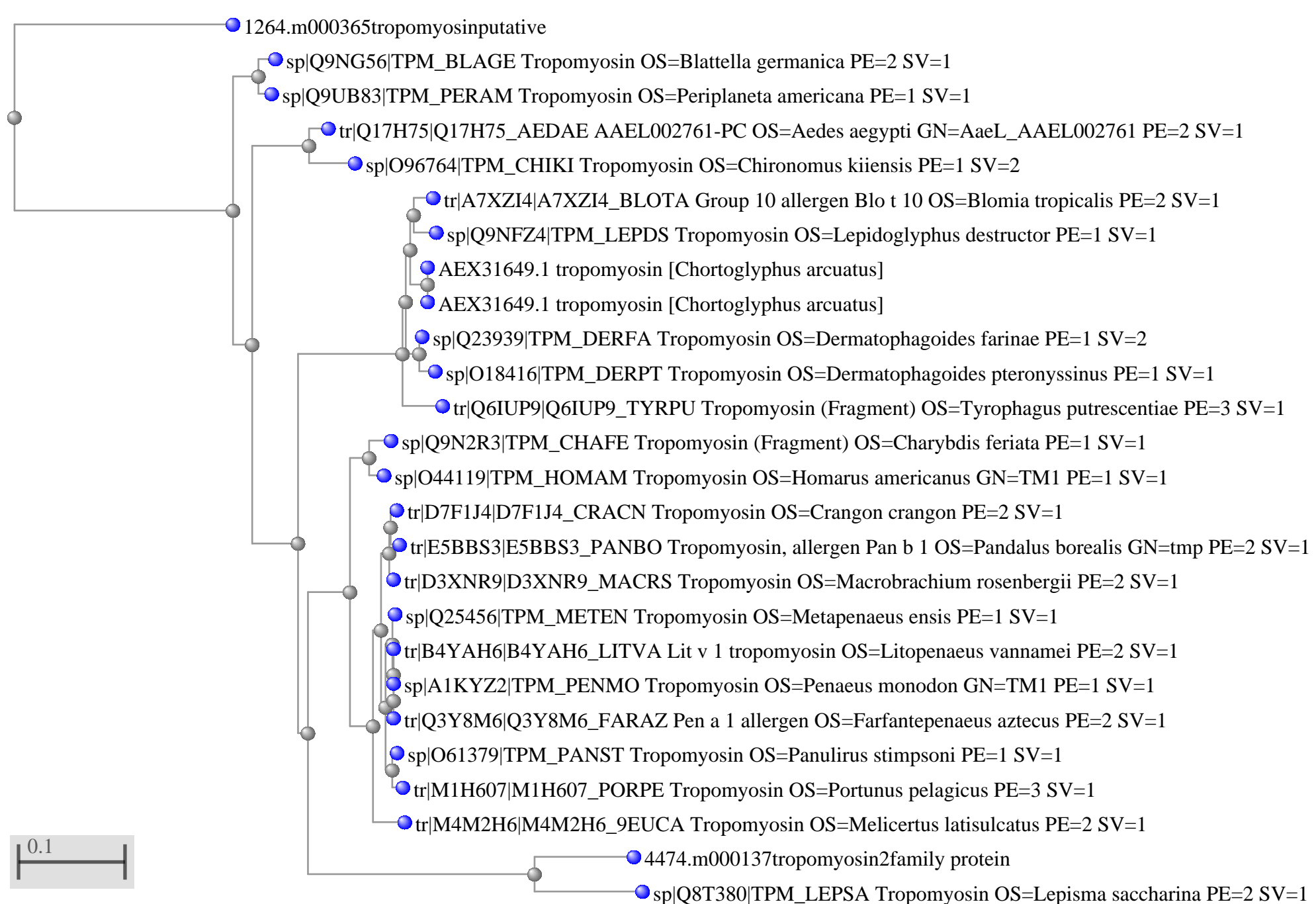

Supplement: S6 Fig — German and American cockroach tropomyosin proteins (Bla g 7 and Per a 7) along with 22 other allergenic tropomyosin proteins included in the IUIS website listed under Animalia Arthropoda were compared with 2 putative C. formosanus tropomyosin proteins (1264.m000365 and 4474.m000137) using the multiple alignment search with the BLASTP suite at ncbi.nlm.nih.gov. The data are represented using the phylogenetic distance tree option and the bar represents the number of amino acid changes per residue. (PDF) [file pone.0182260.s006.pdf]

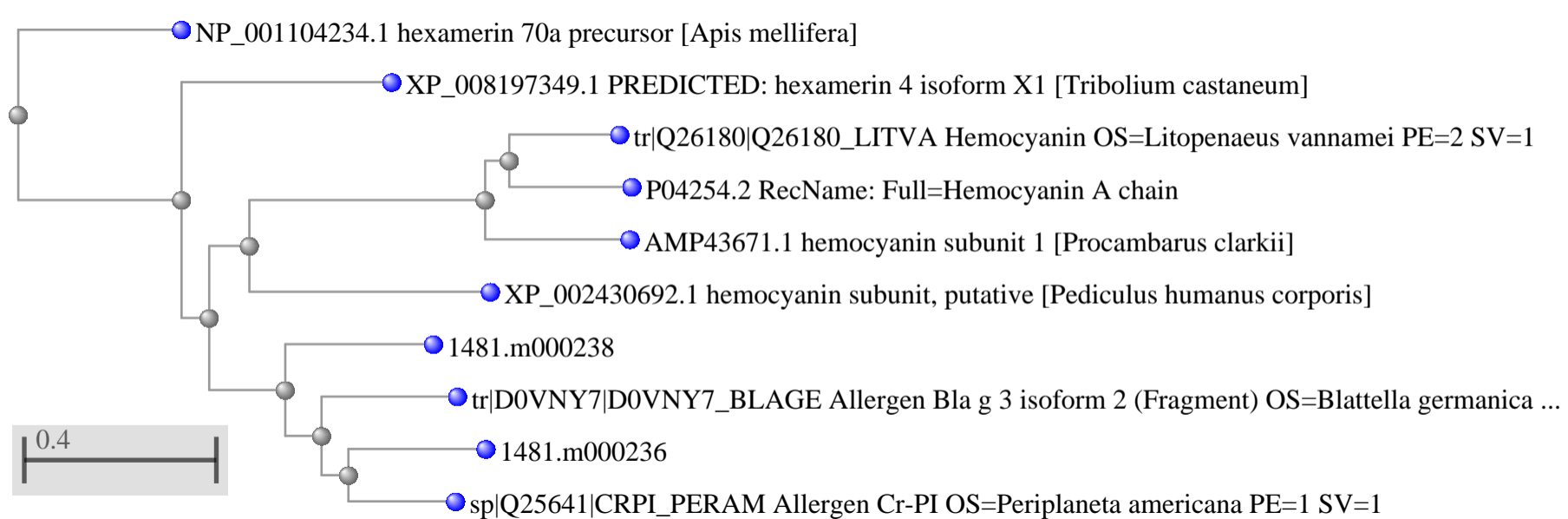

Supplement: S7 Fig — Hexamerin and hemocyanin proteins from 8 species, including the German cockroach Bla g 3 and the American cockroach Per a 3 allergens, were compared with 2 putative C. formosanus hemocyanin proteins (1481.m000238 and 1481.m000236) using the multiple alignment search with the BLASTP suite at ncbi.nlm.nih.gov. The data are represented using the phylogenetic distance tree option and the bar represents the number of amino acid changes per residue. (PDF) [file pone.0182260.s007.pdf]

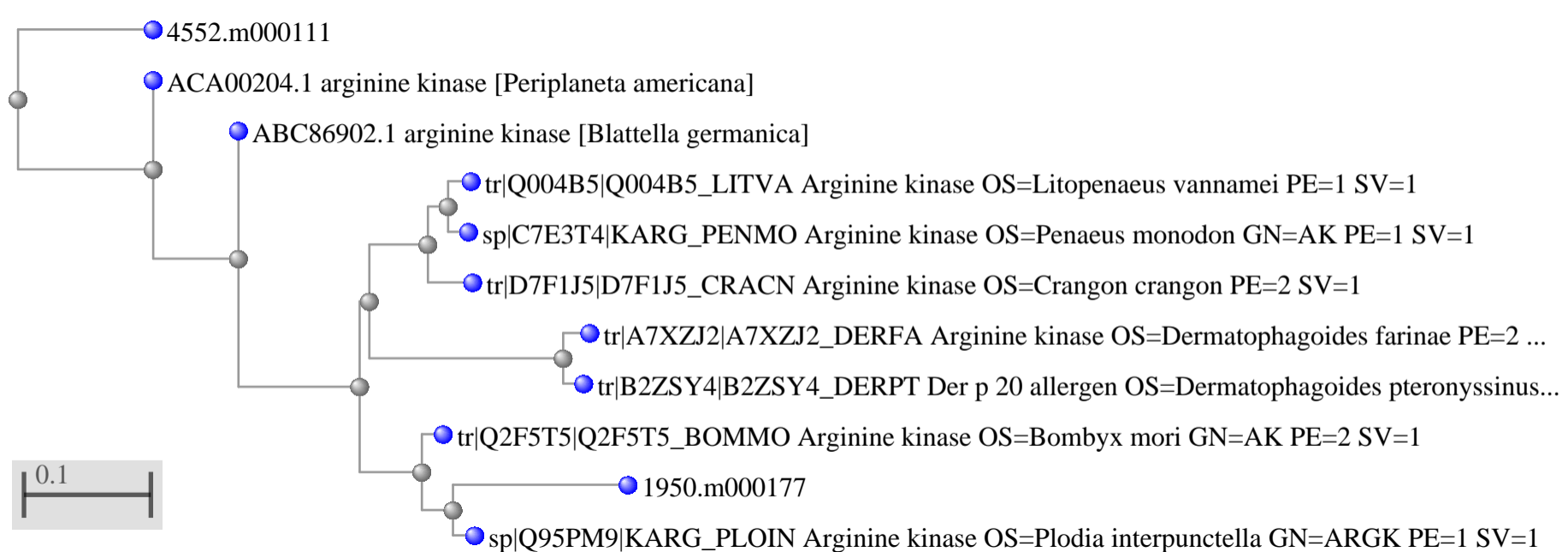

Supplement: S8 Fig — German and American cockroach arginine kinase proteins (Bla g 9 and Per a 9) along with 7 other allergenic arginine kinase proteins included in the IUIS website listed under Animalia Arthropoda were compared with 2 putative C. formosanus tropomyosin proteins (4552.m000111 and 1950.m000177) using the multiple alignment search with the BLASTP suite at ncbi.nlm.nih.gov. The data are represented using the phylogenetic distance tree option and the bar represents the number of amino acid changes per residue. (PDF) [file pone.0182260.s008.pdf]
